# Review of per- and polyfluoroalkyl substances (PFAS) bioaccumulation in earthworms

**DOI:** 10.1016/j.envadv.2022.100335

**Published:** 2022-12-26

**Authors:** Lawrence P. Burkhard, Lauren K. Votava

**Affiliations:** a Great Lakes Toxicology and Ecology Division, Center for Computational Toxicology and Exposure (CCTE), Office of Research and Development, U.S. Environmental Protection Agency, 6201 Congdon Blvd, Duluth, MN 55804 USA.; b Oak Ridge Associated Universities Student Services Contractor to U.S. Environmental Protection Agency, 6201 Congdon Blvd, Duluth, MN 55804 USA

**Keywords:** PFAS, Earthworms, Oligochaetes, Bioaccumulation

## Abstract

Per- and polyfluoroalkyl substances (PFAS) are widely used across the globe in commercial products such textiles, firefighting foams, and surface coatings. Some PFAS, such as perfluorooctane sulfonic acid (PFOS) and perfluorooctanoic acid (PFOA), are known to be bioaccumulative. Numerous terrestrial ecosystems including sites near PFAS manufacturing facilities, facilities using PFAS in their manufacturing processes, firefighting training areas, landfills, and agricultural fields treated with some pesticide formulations, have been contaminated with PFAS. Earthworms reside at the base of the terrestrial food chain and to perform risk assessments at terrestrial sites contaminated with PFAS, information on the bioaccumulation of PFAS is needed. To understand the bioaccumulation of PFAS by earthworms, a literature search was performed, and biota-soil accumulation factors (BSAFs), measured in laboratory tests and at field sites contaminated with PFAS, were assembled and evaluated in this review. Based on this review, we conclude that there is enough data available for carboxylic and sulfonic acid PFAS classes to derive useful BSAFs for terrestrial risk assessments. Laboratory tests with PFOS and PFOA will be close to or at steady-state conditions with standardized testing protocols, and for the longer chain carboxylic and sulfonic acids, it is unlikely they will reach steady-state with the completion of the uptake exposure. For PFAS classes beyond the carboxylic and sulfonic acids, data are limited and performing terrestrial risk assessments with these PFAS will be difficult. Lastly, additional measurements are needed for non-acid PFAS classes as well as from field settings for all PFAS classes. Across all studies, PFOS and PFOA had average (standard deviation, count) BSAFs (kg-OC/kg-ww) of 0.167 (0.311, 60) and 0.0413 (0.175, 47), respectively.

## 1. Introduction

Per- and polyfluorinated alkyl substances (PFAS) present an ongoing concern for environmental and human health (US-EPA, 2021b) and PFAS are a diverse group of >10,776 substances that have multiple carbon-fluorine (Williams et al., 2017). These chemicals have unique characteristics that make them sought after in the production of numerous industrial, commercial, and consumer products, and with their continued production since the 1940s (Banks et al., 2013; Buck et al., 2011), these compounds can be found world-wide in all environmental niches (Giesy and Kannan, 2002).

Based upon chemical structure, OECD/UNEP Global PFC Group divided PFAS universe of chemicals into eight structural categories: carbonyl (100), sulfonyl (200), phosphate (300), fluorotelomer (400), ether (500), PFAA (per- and poly-fluoroalkyl acids) precursors – perfluoroalkyl (600), PFAA precursors – semifluorinated (700), and fluoropolymers (800) based structures (OECD, 2018). In the environment, PFAS in the carbonyl and sulfonyl categories predominant with perfluorooctanoic acid (PFOA) and perfluorooctane sulfonic acid (PFOS) often being the largest portion of the total PFAS in their respective categories (Meng et al., 2021; Zhou et al., 2021; Dasu et al., 2022). The perfluoro-alkyl acids predominate, in part, due to their large historical usage relative to the other PFAS but also due to breakdown/biotransformation of the other PFAS to carboxylic and sulfonyl acids as the end products (Tal and Vogs, 2021; Zhang et al., 2022). PFAS are known be persistent, bioaccumulative and toxic to humans and wildlife, and exposures to PFAS chemicals comes through various media including food, water, air, soil, and sediment (DeWitt, 2015; Brooke et al., 2004; Buck et al., 2011; Suja et al., 2009; Giesy and Kannan, 2002). For the carboxylic acids, bioaccumulation with aquatic species increases with increasing aliphatic chain length for seven carbon and longer carboxylic acids, and for the sulfonic acids, bioaccumulation increases up to aliphatic chain length of eight carbons and then plateaus with increasing chain length (Burkhard 2021). Similar behavior has been observed with earthworms by Rich et al. (2015) and Zhao et al. (2016, 2013).

Areas with notably high concentrations of PFAS are locations where chemical plants make PFAS (Davis et al., 2007), manufacturing facilities using PFAS products in some manner (Barton et al., 2006), and airports, military bases, and fire departments where aqueous film-forming foam (AFFF) have been used in firefighting training. At these training sites, AFFF has contaminated ground and surface waters, sediment, soil, and biota (Hatton et al., 2018; Høisæter et al., 2019). In the United States, numerous military and industrial sites have PFAS contamination, i.e., >1350 sites with PFOA contamination and >1250 sites with PFOS contamination (SSEHRI, 2021). Given the extent of contamination, US-Environmental Protection Agency has developed a national PFAS testing strategy to fill data gaps on these substances (US-EPA, 2021a).

In terrestrial ecosystems, earthworms reside at the base of the food chain. Understanding and quantifying the extent of uptake by earthworms is important because of numerous species that feed upon them, e. g., birds, foxes, moles, shrews, snakes, frogs, snails, salamanders, skunks, and toads. In this report, the bioaccumulation data for PFAS by earthworms are reviewed to derive consensus values for the carboxylic and sulfonic acids for use in risk assessments by regulatory agencies as well as to provide a compilation of current data for subsequent evaluation and interpretation by others. Additionally, difficulties in testing, and gaps and limitations in existing data will be evaluated.

## 2. Materials and methods

Biota-soil accumulation factors (BSAFs) can be measured in field and laboratory settings. In the laboratory setting, standardized protocols are available for measuring the bioaccumulation by oligochaetes (OECD, 2010; ASTM, 2021), and test soils may be dosed in the laboratory with contaminants or may be naturally contaminated soils from field locations. The OECD 317 protocol requires an uptake phase of 21-days or demonstration of steady-state prior to day-21, and an elimination phase starting at the end of the uptake phase (typically day-21) (OECD, 2010). The ASTM protocol requires an uptake phase of 28-days and an elimination phase is not required. The OECD 317 protocol is designed to measure uptake and elimination kinetics via the collection of oligochaete samples over time. The ASTM method is designed to measure uptake after 28 days of exposure with no collection of oligochaete samples over time and no demonstration of steady-state conditions (ASTM, 2021). For the OECD 317 protocol, “steady-state occurs when a plot of the concentration in worms against time is parallel to the time axis, and three successive concentration analyzes made on samples taken at intervals of at least two days do not vary more than ± 20% of each other based on statistical comparisons” (OECD, 2010). The BSAF at steady-state (${\text{BSAF}}_{\text{SS}}$) is determined using the equation:

1 $$BSAF_{SS}=C_{earthworm}/\left(C_{soil}/f_{oc}\right)$$

where ${\text{C}}_{\text{earthworm}}$ is the chemical concentration in the organism (ug/kg-ww) at steady-state, ${\text{C}}_{\text{soil}}$ is the chemical concentration in the soil phase (ug/kg-dw), $f_{\text{OC}}$ is the fraction of organic carbon in the soil, and ${\text{BSAF}}_{\text{SS}}$ has units of kg-OC/kg-ww. The BSAF at day 21 or 28 (${\text{BSAF}}_{\text{d}21}$ or ${\text{BSAF}}_{\text{d}28}$) may or may not be equal to the ${\text{BSAF}}_{\text{SS}}$ depending upon the uptake and elimination kinetics for the chemical. In cases where a chemical does not attain steady-state conditions in the uptake phase, a kinetic BSAF (${\text{BSAF}}_{\text{K}}$) can be determined from the first order uptake and elimination rate constants, and kinetic ${\text{BSAF}}_{\text{K}}$ (kg-OC/kg-ww) is determined using the equation (OECD, 2010):

2 $$BSAF_{K}=k_{s}/k_{e}$$

where ${\text{K}}_{\text{s}}$ is the first order uptake rate constant from soil and ${\text{K}}_{\text{e}}$ is the first order elimination rate constant (OECD, 2010). The ${\text{BSAF}}_{\text{K}}$ equals ${\text{BSAF}}_{\text{SS}}$. The first order uptake and elimination rate constants can be determined using the equations:

3 $$C_{earthworm}=\left(k_{s}/k_{e}\right)\times C_{soil}\times \left(1-e^{-k_{e}t}\right)\;\mathrm{for}\;<t<t_{c}$$

4 $$C_{earthworm}=\left(k_{s}/k_{e}\right)\times C_{soil}\times \left(e^{-k_{e}\left(t-t_{c}\right)}-e^{-k_{e}t}\right)\;\mathrm{for}\;t<t_{c}$$

where ${\text{t}}_{\text{C}}$ is time at the end of the uptake phase and t is time.

Biota soil accumation factors (BSAFs) (kg-OC/kg-ww) can be determined from field measurements and are determined using the equation:

5 $$BSAF=C_{biota}/\left(C_{soil}/f_{oc}\right)$$

BSAFs measured in the field may or may not be at steady-state conditions.

For aquatic oligochaetes, protocols similar to those for terrestrial oligochaetes are available for laboratory testing of sediments (ASTM, 2010; OECD, 2008; US-EPA/US-ACE, 1998; US-EPA, 2000). These protocols have 28-day uptake phase, and some require an elimination phase, i.e., OECD 315 (OECD, 2008). The data analysis is identical to methods described above with the change of soil to sediment in equations 1 through 5.

### 2.1. Literature searching and database structure

A literature search was performed on the bioaccumulation of PFAS in earthworms. The search was implemented by developing a series of chemical-based search terms, e.g., chemical names and Chemical Abstracts Service registry numbers (CASRN or CAS), synonyms, tradenames, and other relevant forms, i.e., metabolites, degradants, parent compounds and related chemicals) (Buck et al., 2011; OECD, 2018, OECD, 2006; US-EPA, 2018). Databases searched were Current Contents, ProQuest CSA, Dissertation Abstracts, Science Direct, Agricola, TOXNET, and UNIFY (database internal to US-EPA’s ECOTOX database (Olker et al., 2022). Table S2 provides the search terms for the initial searches and these searches yielded more than 29,000 citations. After removal of duplicate citations and non-relevant papers, e.g., citations on analytical methods, human health, terrestrial studies with inhalation route of exposure, bacteria, and citations where PFAS was not the chemical of study, the final number of accepted papers for review was approximately 8,200 citations based upon years up through 2018. This searching process was repeated for years 2019, 2020 and 2021 yielding accepted papers for review of approximately 7100, 140,000, and 47,800 citations, respectively. A few reports from 2022 were included in this review and these were found via table of content alerts from various journals. The citations were saved in a RIS file format. Using Swift-Review (Howard et al., 2016), relevant references were identified using search function and the terms (i.e., tags) BAF (bioaccumulation factor), BSAF (biota-soil accumulation factor), worm, oligochaete, bioaccumulation, bioconcentration, uptake, depuration, and accumulation. Studies relevant to bioaccumulation of PFAS in earthworms were selected and coded into a database file. For clarity, a complete listing of all PFAS in the database is provide in Table S1 with their name, abbreviation, CAS number and molecular formula.

In coding the BSAF data, BSAFs were included in the database when no toxicity effects were observed with the earthworms (ASTM, 2021; OECD, 2010, 2008, 2012; US-EPA, 2000). Further, BSAFs placed in the database were required to have complete detection and quantification in all earthworm and soil samples. When concentrations in earthworms and/or soil were below the minimum level of detection (MDL), BSAFs for these data were not included in the database. Therefore, no BSAFs were included in the database based upon ½ MDL or some other estimate of the concentration when concentrations were less than the method MDL.

In the dataset, all the BSAFs were converted to units of kg-organic carbon (OC)/kg-ww by correcting organic matter measurements to OC, assuming a moisture content of 85% in tissue for tissues reported on a dry weight basis, using a default $f_{\text{OC}}$ of 1.55% when no $f_{\text{OC}}$ information was available (Smith et al., 2014), and using a 30% moisture content for measurements reported in soil on a wet weight basis (ASTM, 2021). Organic matter is determined by the loss of ignition (LOI) technique (Heiri et al., 2001) and OC can be estimated from LOI by dividing by 2 (Pribyl, 2010).

The database file has a general information section containing chemical name, chemical abbreviation, CAS, citation, and earthworm species name. For laboratory BSAF measurements, the endpoint and when available, kinetic information and concentration data for tissue and water, were extracted from the primary literature. For field BSAF measurements, the endpoint and when available, sampling dates, number of samples and concentration data for tissue and soil, were extracted.

Included in the database file is a generalized ranking system for study quality of high, medium, and low. For laboratory BSAFs, tests given a high ranking were performed according to OECD 317 protocol (OECD, 2010), where uptake and depuration phases were performed. Tests given a medium ranking demonstrated steady-state conditions and only the uptake phase of OECD 317 was performed. Tests given a low ranking did not demonstrate steady-state conditions and/or used nominal exposure concentrations.

For field measurements of BSAFs, four criteria were evaluated, i.e., number of soil samples; number of organism samples; temporal coordination of soil and organism samples; and spatial coordination of soil and organism samples. For numbers of samples, when greater than 3 samples were collected, this criterion is assigned a value of 1; a value of 2 is assigned when only 2 or 3 samples were collected; and a value of 3 is assigned when only 1 sample was collected. The temporal coordination criterion is assigned a value of 1 when samples were collected simultaneously; assigned a value of 2 when samples were collected within a one-year time window; and assigned a value of 3 when samples were collected over a time period of greater than one year. The spatial coordination criterion was assigned a value of 1 when soil and organisms were collected from the same location; and assigned a value of 3 when soil and organisms were collected from different sampling locations. Studies are ranked high-, medium-, and low-based off the cumulative result from the four criteria above. Studies with a high quality ranking have sums of 4 or 5; medium quality rankings have sums of 6 or 7; and low quality rankings have sums ranging from 8 to 10. In general, studies with high-, medium-, and low-quality ranking are somewhat equivalent to the Klimisch et al. (1997) data quality system, i.e., Klimisch scores of 1 (“Reliable”), 2 (“Reliable with Restrictions”) and 3 (“Not Reliable”).

### 2.2. Data Analysis

Statistical comparisons were performed using R-Studio software (RStudio Team, 2020) using anova, t.test, and TukeyHSD (R Core Team, 2022). Statistical evaluations were performed on log transformed BSAFs because the BSAFs were log-normally distributed. In comparisons where there was only one measurement for a group where anova algorithm could not be used, the t.test algorithm was used. With the t.test algorithm, the confidence of the single measurement residing within the distribution of the other measurements was determined.

## 3. Results and discussion

The bioaccumulation data set for terrestrial oligochaetes (earthworms) was assembled from 25 papers and reports. In the data set, BSAFs are first provided using the units from their citation, and secondly, after conversion to units of kg-OC/kg-ww. BSAFs discussed henceforth in this report use units of kg-OC/kg-ww. One of the difficulties in evaluating BSAFs from laboratory studies was determining if steady-state conditions were obtained. If steady-state conditions could not be ascertained for a study, the measurements were given a low study quality ranking. The data set contains 424 BSAFs (whole body tissues) for earthworms with 23%, 26% and 51% of the BSAFs having high, medium, and low study quality rankings, respectively.

BSAFs measurements have been done with terrestrial oligochaete species *Eisenia fetida* (number of reports=21)*, Eisenia andrei* (2)*, Lumbriculus terrestris* (2), and *Metaphire guillelmi* (1), and in one field study, a mixture of *Lumbricus rebellus, Aporrectodea rosea, Dendrobaena octaedra*, and *Dendrodrilus rubidus* oligochaetes were analyzed. For the *M. guillelmi*, BSAF data are available only for 6:2 diPAP (Bis (3,3,4,4,5,5,6,6,7,7,8,8,8-tridecafluorooctyl) hydrogen phosphate) (Zhu et al., 2021) and no other species have measurements for this chemical. For the *E. fetida, E. andrei,* and *L. terrestris* species, their BSAFs were not significantly different for the carboxylic and sulfonic acids (α=0.05, see Table S3). The field samples with a mixture of species were significantly different from the *E. fetida, E. andrei,* and *L. terrestris* species for PFHxA (perfluorohexanoic acid), PFOA, PFNA (perfluorononanoic acid), PFDA (perfluorodecanoic acid), and PFBS (perfluorobutane sulfonic acid) and not significantly different for the other five carboxylic and sulfonic acids (see Table S3). Comparisons of BSAFs based upon study quality rankings of high, medium, and low revealed a few significantly different BSAFs among the ranking categories (3 of 26 comparisons) (α=0.05, see Table S4). Comparisons of BSAFs by study location (laboratory vs field) revealed 9 of 25 comparisons were significantly different (α=0.05, see Table S5). In the latter two comparisons, the PFAS that were significantly different had low sample numbers, e.g., 1 or 2 data points in a group, and high variances in the data. Low sample numbers decrease the confidence of significant differences in the BSAFs among study quality groups and locations. Consensus BSAFs are provided in Table 1 for the carboxylic and sulfonic acids derived from all data. The distributions of their BSAFs are provided in Supporting Information (Fig. S1).

**Fig. 1.**
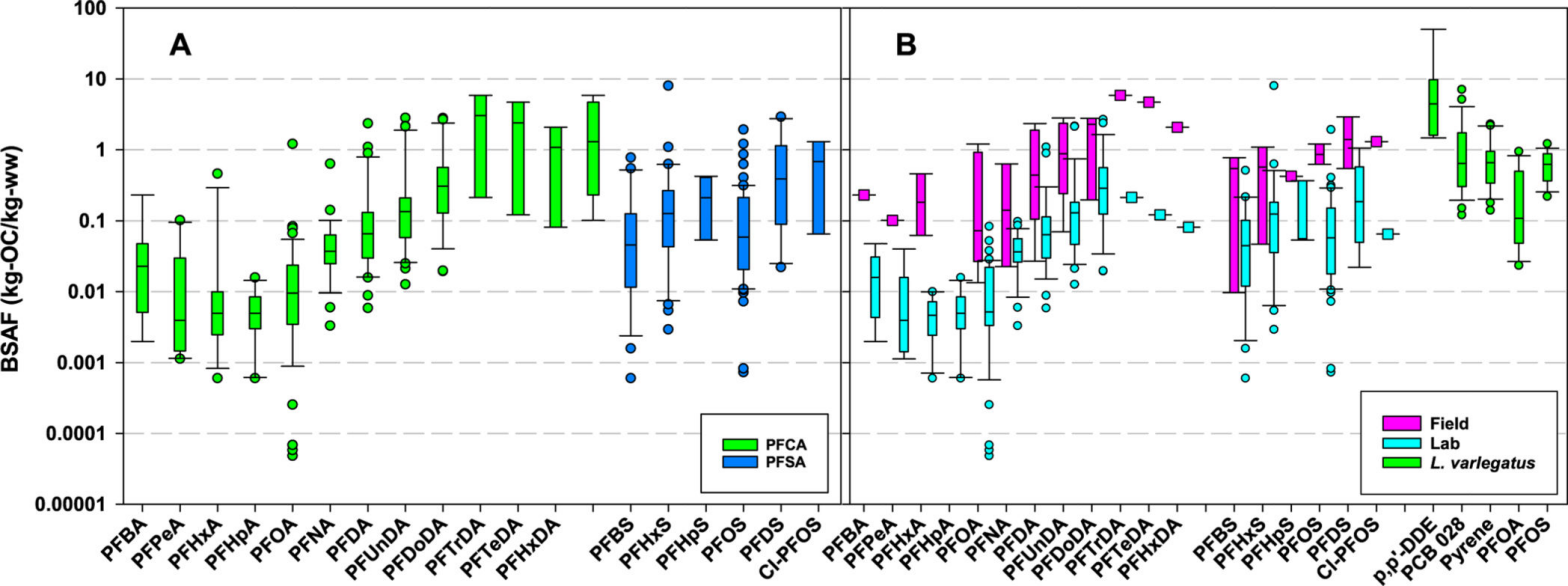
(A) Boxplots of BSAFs (Biota-Soil Accumulation Factors) for all perfluoro-carboxylic (PFCA) and -sulfonic (PFSA) acids, (B) Boxplots of BSAFs from laboratory and field sampling locations for carboxylic and sulfonic acids, and boxplots of BSAFs from laboratory measurements with *Lumbriculus variegatus*. The carboxylic and sulfonic acids are ordered from left to right by increasing number of carbons in the aliphatic chain. The lines in the boxes are median, ends of the boxes the 25 and 75th percentiles, whiskers are 10 and 90th percentiles; and dots are individual values outside the whiskers. In plot B, when only one measurement exists for a chemical, a box symbol is plotted. PFBA = Perfluorobutanoic acid, PFPA = perfluoropentanoic acid; PFHxA = perfluorohexanoic acid; PFHpA = perfluoroheptanoic acid; PFOA = perfluorooctanoic acid; PFNA = perfluorononanoic acid; PFDA = perfluorodecanoic acid; PFUnDA = perfluoroundecanoic acid; PFDoDA = perfluorododecanoic acid; PFTrDA = perfluorotridecanoic acid; PFTeDA = perfluorotetradecanoic acid; PFHxDA = perfluorohexadecanoic acid, PFBS = perfluorobutane sulfonic acid; PFHxS = perfluorohexane sulfonic acid; PFHpS = perfluoroheptane sulfonic acid; PFOS = perfluorooctane sulfonic acid; PFDS = perfluorodecane sulfonic acid; Cl-PFOS = 1-chloroperfluorooctanesulfonic acid; p,p’-DDE = 1,1-Dichloro-2,2-bis(4-chlorophenyl)ethene; PCB 028 = 2,4,4′-Trichlorobiphenyl.

The trends in BSAF data are shown in Fig. 1A. The data mimic the trends observed by others for the carboxylic and sulfonic acids for bioaccumulation factors (BAFs) with aquatic species (Chen et al., 2018; Koch et al., 2019; Liu et al., 2018; Martin et al., 2003). For the carboxylic acids, PFBA (perfluorobutanoic acid) up through PFOA in carbon chain length have low and similar values, and then, with increasing carbon chain length beyond PFOA, BSAFs increase. The BSAFs for the carboxylic acids range from median value of 0.00954 for PFOA to 3.04 for PFTrDA (perfluorotridecanoic acid). For carboxylic acids larger than PFTrDA, the BSAFs decline with increasing carbon chain length from the value of PFTrDA. However, the limited numbers of measurements for these larger acids makes interpretation of the trend a bit tenuous. For the sulfonic acids, BSAFs for PFBS (perfluorobutane sulfonic acid) are low (median of 0.0459) and with increasing carbon chain length, the BSAFs rise to a median value 0.39 for PFDS (perfluorodecane sulfonic acid). Cl-PFOS (chloro-perfluorooctanesulfonic acid) has a slightly higher BSAF of 0.688 in comparison to PFOS (0.0587) and this might be due to the presence of the chlorine on the aliphatic chain.

Although there are some statistically significant differences in BSAFs between laboratory and field study locations, these comparisons are limited because earthworms collected from contaminated sites are from three studies (Munoz et al., 2020; Zhu and Kannan, 2019; Amundsen et al., 2008) and many of the PFAS were only measured in one of the studies. Plotting of the laboratory and field BSAFs reveals patterns like that of the data combined (Fig. 1B). Included in Fig. 1B are laboratory measured BSAFs for aquatic oligochaete *Lumbriculus variegatus* for three legacy contaminants (US-ACE, 2022) and PFOS and PFOA. The ranges in the laboratory measured BSAFs for earthworms and *L. variegatus* are similar and indicates no major differences in BSAF variability between legacy contaminants and PFAS, and between species. The BSAFs measured in the field are, in general, larger and have a much wider range in comparison to the BSAFs measured in the laboratory, and the limited number of studies performed in the field makes it difficult to determine if this behavior is typical or atypical. If this behavior is genuine, causes of the differences in accumulation are unknown. Additionally, the large range in field BSAFs for earthworms is difficult to explain and this range might be related to bioavailability and/or sampling issues in the field studies.

**Fig. 2.**
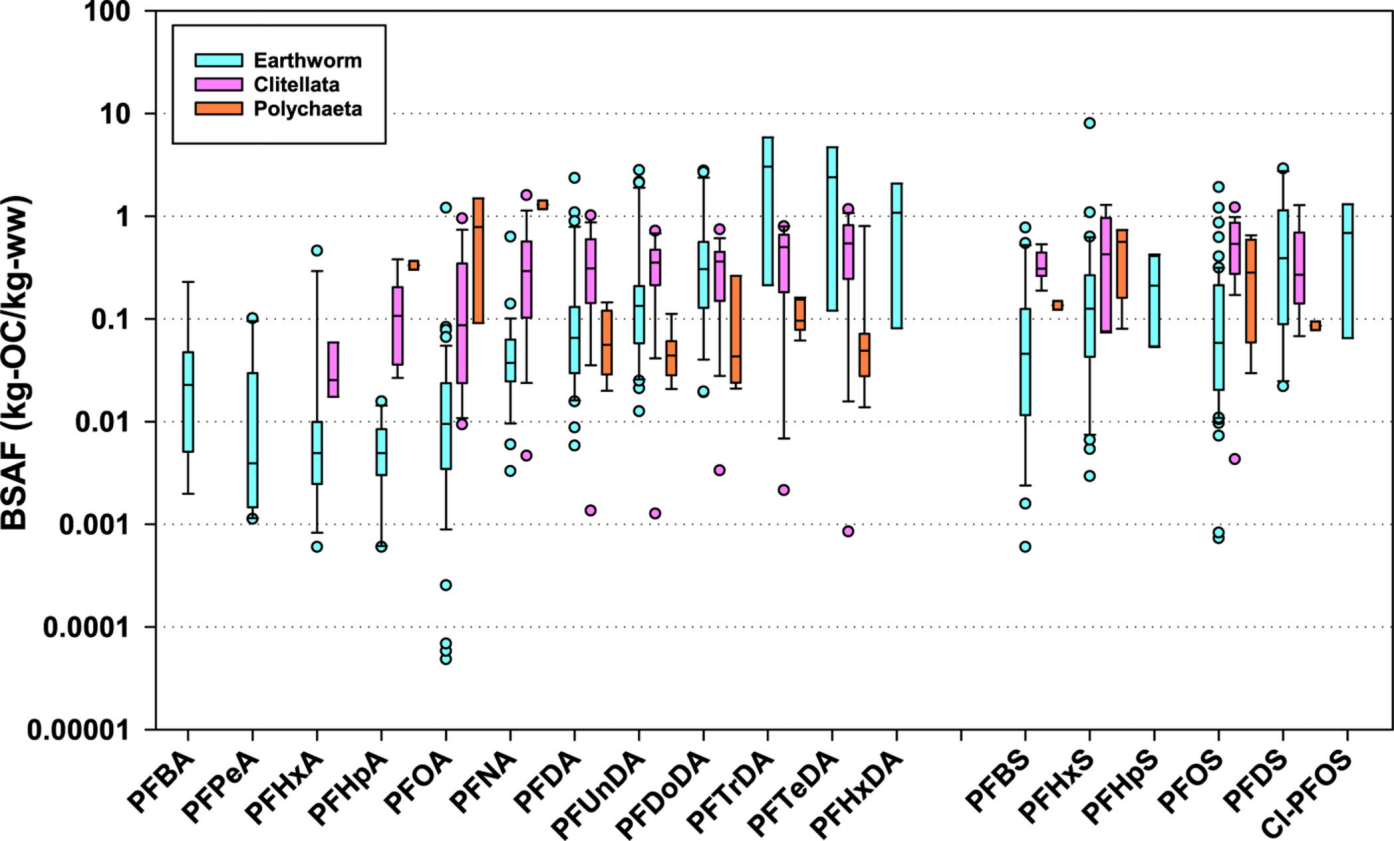
Boxplots of BSAFs (Biota-Soil Accumulation Factors) for carboxylic and sulfonic acids for earthworms (terrestrial oligochaetes), aquatic oligochaetes (Clitellata), and Polychaetes. The carboxylic and sulfonic acids are ordered from left to right by increasing number of carbons in the aliphatic chain. The lines in the boxes are median, ends of the boxes the 25 and 75th percentiles, whiskers are 10th and 90th percentiles; and dots are individual values outside the whiskers. . PFBA = Perfluorobutanoic acid, PFPA = perfluoropentanoic acid; PFHxA = perfluorohexanoic acid; PFHpA = perfluoroheptanoic acid; PFOA = perfluorooctanoic acid; PFNA = perfluorononanoic acid; PFDA = perfluorodecanoic acid; PFUnDA = perfluoroundecanoic acid; PFDoDA = perfluorododecanoic acid; PFTrDA = perfluorotridecanoic acid; PFTeDA = perfluorotetradecanoic acid; PFHxDA = perfluorohexadecanoic acid, PFBS = perfluorobutane sulfonic acid; PFHxS = perfluorohexane sulfonic acid; PFHpS = perfluoroheptane sulfonic acid; PFOS = perfluorooctane sulfonic acid; PFDS = perfluorodecane sulfonic acid; Cl-PFOS = 1-chloroperfluorooctanesulfonic acid.

For further comparison purposes, BSAFs for aquatic oligochaetes (*L. variegatus* and *Tubifex tubifex)* and aquatic polychaetes (*Capitellidae, Nereidae, Sabellidae, Nereis diversicolor, and Arenicola marina*) are shown in Fig. 2 along with the earthworm BSAFs. The BSAFs among earthworms (terrestrial oligochaetes), aquatic oligochaetes, and aquatic polychaetes are in reasonable agreement. Given the biological similarities among the three groups (all are Annelids), reasonable agreement among three groups was somewhat expected. This comparison is a bit difficult because polychaete data are limited and from field measurements only, and measurements with earthworms and aquatic oligochaetes are primarily from laboratory studies. Further, the laboratory measurements include BSAFs from steady-state and unknown/non-steady-state conditions, and field measurements are, in general, from pseudo-steady-state conditions. The BSAFs across the three species groups (Fig. 2) are, for the most part, less than 1 for the sulfonic acids (Fig. 2) and less than 5 for carboxylic acids. For earthworms, average BSAFs for PFOS and PFOA were 0.167 (sd=0.311, *n*=60) and 0.0413 (0.175,47), respectively, and resulting in 4-fold difference in bioaccumulation between the two chemicals.

**Fig. 3.**
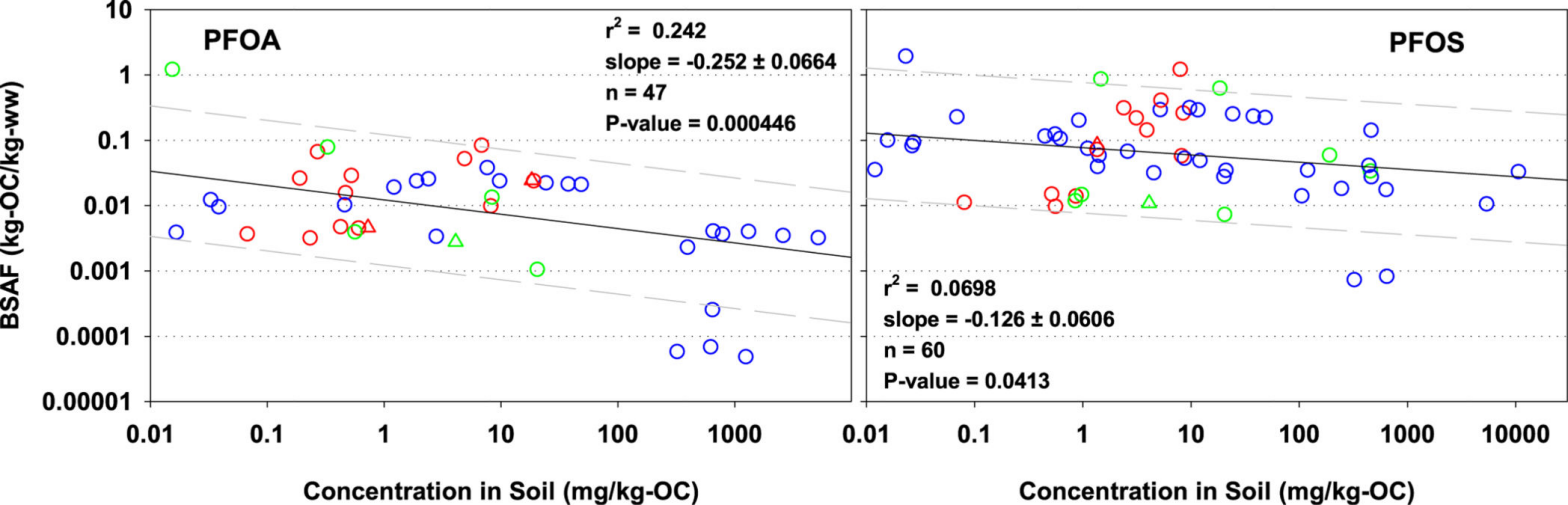
Plots of BSAFs (kg-OC/kg-ww) for PFOS and PFOA against concentration in soil (mg/kg-OC). Regression line (solid) ($\log\text{BSAF}=\text{A}+\text{Blog}{\text{C}}_{\text{SOC}}$) and statistics (slope ± standard error) along with lines 10-fold higher and lower (dash) are shown in BSAF vs concentration soil plots. CSOC = Concentration of PFOS or PFOA in soil on an organic carbon basis. BSAF = Biota-Soil Accumulation Factor, PFOS = perfluorooctane sulfonic acid; PFOA = perfluorooctanoic acid.

In performing laboratory bioconcentration tests, numerous investigators have reported bioconcentration factors (BCFs) declining with increasing exposure concentrations for aquatic organisms (Chen et al., 2019; Dai et al., 2013; Fernández-Sanjuan et al., 2013; Hoke et al., 2015; Inoue et al., 2012; Liu et al., 2011). Similarly, Zhao et al. (2013, 2014) and Wen et al. (2015) have observed BSAFs declining with increasing concentrations in the soil with *E. fetida* in laboratory studies. In the assembled earthworm BSAF data set, the BSAF data have this behavior (Fig. 3 for PFOA and PFOS, Fig. S1 for other PFAS). However, there is a fair amount of variability in the data even though the regressions are statistically significant. The trend line suggests a factor of 1.34 and 1.56 decline in BSAF value with each order of magnitude increase in concentration in the soil for PFOS and PFOA, respectively. Other PFAS chemicals behave similarly (Fig.S1).

**Fig. 4.**
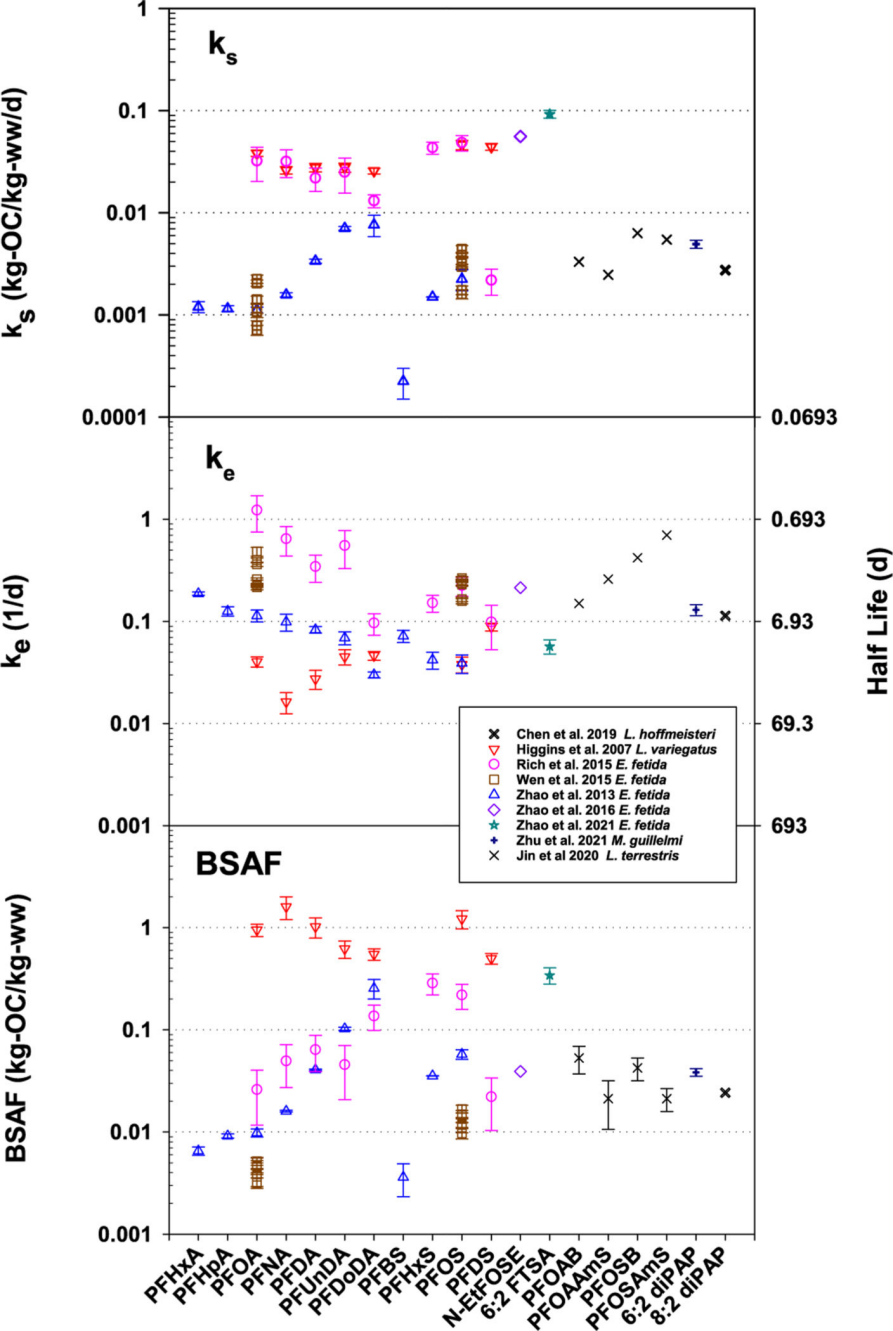
Plots of uptake rate (${\text{k}}_{\text{s}}$), elimination rate (${\text{k}}_{\text{e}}$), and ${\text{BSAF}}_{\text{K}}\text{s}$ for PFAS with earthworms. BSAF = Biota-Soil Accumulation Factor; PFAS = Per- and Polyfluoroalkyl Substances; PFHxA = perfluorohexanoic acid; PFHpA = perfluoroheptanoic acid; PFOA = perfluorooctanoic acid; PFNA = perfluorononanoic acid; PFDA = perfluorodecanoic acid; PFUnDA = perfluoroundecanoic acid; PFBS = perfluorobutane sulfonic acid; PFHxS = perfluorohexane sulfonic acid; PFOS = perfluorooctane sulfonic acid; PFDS = perfluorodecane sulfonic acid; N-EtFOSE = 2-Perfluorooctylsulfonyl-N-ethylaminoethyl alcohol; 6:2 FTS = 6:2 Fluorotelomer sulfonic acid, PFOAB = (Dimethyl{3-[(perfluorooctanoyl)amino]propyl}ammonio)acetate; PFOAAmS = Perfluorooctaneamido ammonium; PFOSB = {[(Perfluorooctyl)sulfonyl] amino}−3-betaine;6:2 diPAP = Bis(3,3,4,4,5,5,6,6,7,7,8,8,8-tridecafluorooctyl) hydrogen phosphate, 8:2-diPAP = Bis(3,3,4,4,5,5,6,6,7,7,8,8,9,9,10,10,10-heptadecafluorodecyl) hydrogen phosphate.

Limited uptake and elimination rate data are available for earthworms, and these data and their ${\text{BSAF}}_{\text{K}}\text{s}$ are shown in Fig. 4 and Table S6 along with data for aquatic oligochaetes. For the carboxylic and sulfonic acids, uptake rates are comparable between Wen et al. (2015) and Zhao et al. (2013) for PFOS (0.0030 and 0.0023 kg-OC/kg-ww/d, respectively) and PFOA (0.0014 and 0.0011, respectively) while the rates measured by Rich et al. (2015) are larger by more than order of magnitude (0.049 and 0.032, respectively). For the other acids, uptake rates measured by Zhao et al. (2013) are slower than the rates measured by Rich et al. (2015), i.e., slower by factors of 29, 22, 6.5, 3.5, and 1.7 for PFHxS (perfluorohexane sulfonic acid), PFNA, PFDA, PFUnDA (perfluoroundecanoic acid), and PFDoDA (perfluorododecanoic acid), respectively. Elimination rates for Rich et al. (2015) were 3 to 11 times faster then those reported by Zhao et al. (2013). In contrast, the elimination rate for PFOS measured by Wen et al. (2015) agreed with the rate reported by Rich et al. (2015), i.e., 0.22 1/d, and elimination rate for PFOA (0.31 1/d) agreed with rate reported by Zhao et al. (2013) of 0.11 1/d. The resulting ${\text{BSAF}}_{\text{K}}\text{s}$ for PFOS and PFOA across the three studies varied more than an order of magnitude, i.e., 0.013, 0.058, and 0.22 for PFOS and 0.0042, 0.0098, and 0.026 for PFOA for Wen et al. (2015), Zhao et al. (2013) and Rich et al. (2015), respectively. Causes for these differences in rates and ${\text{BSAF}}_{\text{K}}\text{s}$ are not known. Rich et al. (2015) suggested the differences between their rates and (Zhao et al., 2013) might be related to equilibration time of the spiked farmland soil used in Zhao et al. (2016) exposures. However, the studies by Rich et al. (2015) and Wen et al. (2015) used soils receiving long-term additions of biosolids containing the PFAS and the differences between these two studies suggest equilibration time of the spikes is not the only cause of the differences in measured uptake rates. The exposures by Zhao et al. (2013) and Wen et al. (2015) included uptake and elimination measurements whereas only uptake exposures were performed by Rich et al. (2015). For the kinetic studies, ${\text{BSAF}}_{\text{K}}\text{s}$ are shown in Fig. 4 and Table S6 along with ${\text{BSAF}}_{\text{K}}\text{s}$ for aquatic oligochaetes, and there is some scatter data. The resulting ${\text{BSAF}}_{\text{K}}\text{s}$ form ranges for PFOA of 0.0032 to 0.026 (8.2 fold range) and for PFOS of 0.0097 to 0.22 (22.3 fold range) for the earthworms. For the other PFAS, ranges are smaller, e.g., 0.046–0.10 for PFUnDA, but data are limited to just two studies.

In Fig. 4, uptake and elimination rates, and ${\text{BSAF}}_{\text{K}}\text{s}$ for aquatic oligochaetes are provided for comparison to the earthworms. The *L. variegatus* have the fastest uptake rates, slowest elimination rates, and largest BSAFs. Their BSAFs are nearly independent of carbon chain length, similar across the carboxylic and sulfonic acids groups, and larger in comparison to those measured for earthworms. The uptake by *L. variegatus* might be faster in comparison to earthworms because their exposure via skin to sediment pore waters containing PFAS. For the PFAS beyond the carboxylic and sulfonic acids groups, their rates and ${\text{BSAF}}_{\text{K}}\text{s}$ reside within the range of values for the PFAS acids. For some of these PFAS, biotransformation might be important, and biotransformation will enhance their elimination rates (Jin et al., 2020; Zhao et al., 2016).

The OECD 317 and ASTM laboratory testing methods have uptake exposure periods of 21 and 28 days, respectively. Chemicals with half-lives of 4.85 and 6.48 (days) will reach 95% of their steady-state values in the 21- and 28-day uptake exposures, respectively. These half-lives translate to elimination rates of 0.143 and 0.107 (1/d), respectively, and eliminations rates of 0.01, 0.1, 1.0, and 10 (1/d) (in Fig. 3b) translate to half-lives of 69.3, 6.93, 0.693 and 0.0693 days for reaching 95% of steady-state conditions in the laboratory tests. These results suggest that measurements of BSAFs for PFOA and PFOS in the laboratory will be close to or at steady-state conditions with OECD 317 and ASTM test methods. For the longer chain carboxylic and sulfonic acids, it is unlikely they will reach steady-state with the completion of the uptake exposure and thus, requiring measurement of both uptake and elimination rate constants to determine steady-state BSAFs (using Eq. (2)). Extending the uptake exposure time to 42 or 56 days allows PFAS with half-lives of 9.7 and 13 (d) or in terms of elimination rates, 0.071 and 0.053 (1/d), to attain steady-state conditions in an uptake exposure. For the non-acid PFAS, their half-lives suggest accumulation in the earthworms will be very close to or at steady-state in the standardized testing protocols. Some of the range in BSAFs in Fig. 1a is due to the combining of steady-state with non-steady-state BSAFs.

## 4. Gaps and limitations and difficulties in testing

BSAFs are limited for chemicals beyond carboxylic and sulfonic acids classes, e.g., phosphate, fluorotelomer, and ether-based PFAS. Additionally, newer replacement PFAS such as perfluoro-betaines and hexafluoropropylene oxide-dimer acid (Joerss et al., 2020; Kaboré et al., 2022) need measurements. The lack of measurements is a major gap.

The limited number of field studies is also a major gap. Data from the three studies to date (Munoz et al., 2020; Zhu and Kannan, 2019; Amundsen et al., 2008) suggest that accumulation in the field might be different from that observed in the laboratory. Without additional field measurements, understanding and resolving if there are differences in accumulation between laboratory and field settings by earthworms cannot be performed. Numerous contaminated sites have toxicity and bioaccumulation testing performed in the laboratory during the remedial investigation. Proper use of the laboratory testing results in risk assessments and designs for remedial activities at contaminated sites is dependent upon knowing if this difference exists.

Another major limitation is understanding the mechanisms for BSAFs declining with increasing exposure concentrations. Numerous studies (Wen et al., 2015; Zhao et al., 2014, 2013) and the collective data assembled in this report (Fig. 3) have this behavior. For situations where cleanup activities could potentially occur, this behavior could dramatically influence the overall remedial design and costs.

Studies addressing the question of bioaccumulation when different PFAS mixtures are present are lacking. With fish, suppression of accumulation of shorter chain carboxylic acids occurred when longer chain acids were present in bioconcentration tests (Wen et al., 2017). The effects of mixtures on bioaccumulation by earthworms is a data gap.

Kinetic uptake and elimination data are limited and for the development of simple and complex models for forecasting bioaccumulation in earthworms, better data are needed. Part of the deficiencies in kinetic rate data are the dependence of rates upon soil composition, e.g., two studies with soils with long term additions of biosolids yielded rates that were greater than order of magnitude different, e.g., ${\text{k}}_{\text{s}}$ of 0.0487 and 0.00305 (kg-OC/kg-ww/d) for PFOS, respectively (Rich et al., 2015; Wen et al., 2015).

Another large data gap is understanding the expected BSAF values for PFAS for earthworms. This gap is caused, in part, by insufficient data to develop a modeling framework for forecasting BSAFs for PFAS. In contrast, for nonpolar organic chemicals in sediments (e.g., PCBs, DDTs and PAHs), the expected BSAFs for oligochaetes and other benthic invertebrates should be in the 1–2 (kg-OC/kg-lipid) range based upon EqP theory (Toro et al., 1991; Lake et al., 1990; Tracy and Hansen, 1996). EqP theory assumes that nonpolar organic chemicals have similar affinities for lipids and sediment organic carbon and results in the BSAFs being independent of chemical class and hydrophobicity of the chemicals. PFAS do not have similar affinities for lipids and soil/sediment organic carbon and as a result, EqP theory does not apply to PFAS. In fish and mammalian species, proteins, phospholipids, and active transport pathways control the tissue distribution of PFAS (Armitage et al., 2013, 2017; Dassuncao et al., 2019; Ng and Hungerbühler, 2013, 2014; Han et al., 2012; Weaver et al., 2009) and model(s) accounting for partitioning behavior to soils and with proteins, phospholipids, and active transport pathways of the oligochaetes are needed. PFAS BSAFs from laboratory studies are almost always less than 1 (Fig. 1) and for some PFAS, much lower than 1. As discussed above, BSAFs from field studies provide higher BSAFs and causes of this are known.

**Fig. 5.**
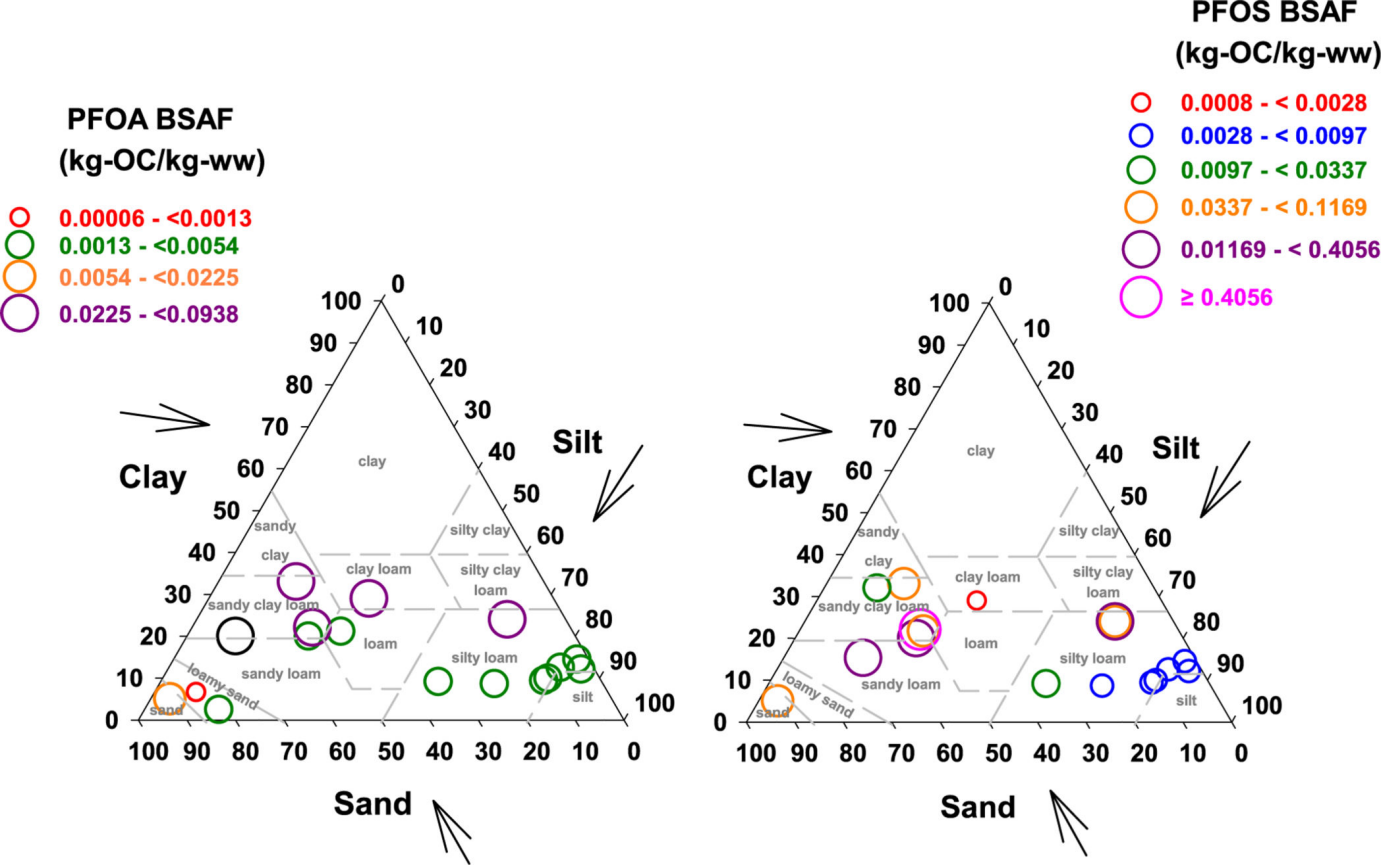
Soil texture triangle plots of PFOS and PFOA BSAFs. BSAF = Biota-Soil Accumulation Factor, PFOS = perfluorooctane sulfonic acid; PFOA = perfluorooctanoic acid.

One of the issues in defining the expected BSAF values for PFAS is the lack of model(s) for predicting their bioavailability in the soils. Bioavailability in soils is dependent upon the soil/water partition coefficient (${\text{K}}_{\text{d}}$) and research has shown that ${\text{K}}_{\text{d}}$ is a function of a number of soil parameters including organic carbon content (Higgins and Luthy, 2006; Jia et al., 2010; Johnson et al., 2007; Fabregat-Palau et al., 2021) and inorganic composition (Fabregat-Palau et al., 2021; Li et al., 2018). A model for predicting ${\text{K}}_{\text{d}}$ developed by Fabregat-Palau et al. (2021) uses $f_{\text{OC}}$, the sum of silt and clay fractions, and soil organic carbon-water and soil mineral-water partition coefficients. In the assembled BSAF database, approximately half of the studies reported sand, clay and silt contents of the soils studied (Table S7). Plotting of the BSAFs for PFOS and PFOA on soil texture triangle (Shirazi and Boersma, 1984) has, in general, larger BSAFs for soils with higher sand contents (Fig. 5). The location of the BSAFs in soil text triangle is consistent with model of Fabregat-Palau et al. (2021) where higher sand contents predicts smaller ${\text{K}}_{\text{d}}\text{s}$. With smaller ${\text{K}}_{\text{d}}\text{s}$, PFAS is less tightly sorbed to soils and this translates to higher availability to the earthworms. There is need for research to understand PFAS partitioning and how this partitioning translates into sequestration and bioavailability of PFAS to earthworms (Zhao et al., 2016).

When performing soil bioaccumulation tests, ideally, one would like constant concentration in the soil over time, be able to demonstrate steady-state conditions for the BSAF, and be able to measure ${\text{K}}_{\text{s}}$ and ${\text{k}}_{\text{e}}$. However, some chemicals degrade in the soils over the testing period resulting in declining concentrations over time. For example, in a soil dosed with 10:2 FTOH (10:2 fluorotelomer alcohol), 7 and 25% of its initial concentration remained after a 30-d exposure with and without earthworms, respectively (Zhao and Zhu, 2017). If steady-state BSAFs, i. e., non-changing BSAFs over time, cannot be demonstrated, measurement of the ${\text{k}}_{\text{s}}$ and BSAF becomes much more difficult. In these cases, time course concentration data in the soil with and without earthworms, and in the earthworms will be needed. With these data, a dynamic one-compartment bioaccumulation model with biotransformation in the earthworm (Grech et al., 2017) would be developed whereby allowing estimation of ks, ke, BSAF, and ${\text{k}}_{\text{M-earthworm}}$ (biotransformation rate within the earthworm). The dynamic solution of the bioaccumulation model would also require the ordinary differential equation describing the concentrations in soil over time. Note, measurement of the elimination rate constant (${\text{k}}_{\text{e}}$) will be possible in these cases, since, in the elimination phase, the soil will have none of the test chemical in it.

Another difficulty in performing soil bioaccumulation tests for earthworms is the availability of individual compounds and their availability in quantities needed for dosing soils. One can easily test PFAS commercial formulations such as AFFF. Unfortunately, testing complex mixtures like AFFFs makes it difficult to tease out the actual bioaccumulation potential of an individual PFAS because the chemical of interest might be a breakdown product of more complex PFAS. Additionally, reference materials to identity and quantify individual PFAS are limited, and bioaccumulation testing with complex mixtures, e.g., AFFF-impacted soil from an airport, can lead to numerous qualitative results for tentatively identified PFAS (Munoz et al., 2020).

## 5. Summary

This literature review pulls together PFAS data for BSAFs for earthworms in a form that others can easily access and use for their purposes. Laboratory measured BSAFs for the carboxylic and sulfonic acids are available whereas data for other PFAS classes are very limited. It is suggested that in working with the BSAF data preference be given to values from studies with high- and medium-studies qualities before using studies with low quality. This review highlights data gaps and deficiencies including the lack of measurements for phosphate, fluorotelomer, and ether-based PFAS, limited uptake and elimination rate data, and the expected BSAF values for PFAS.

## Supplementary Material

Supplement1

Supplement2

## Figures and Tables

**Table 1:** Measured BSAFs (kg-OC/kg-ww) for earthworms.

		Laboratory			Field			Laboratory and Field Combined							
				
Chemical	CAS	average	standard deviation	n	average	standard deviation	n	average	median	standard deviation	n	Percentile 10th	Percentile 90th	Minimum	Maximum

Carbonyl Compounds - OECD Structure Category 100															
Carboxylic acids - OECD Structure Category 102															
PFBA	45048-62-2	0.0186	0.017	6	0.23 *	-	1	0.0488	0.0229	0.0814	7	0.00386	0.120	0.00198	0.23
PFPeA	45167-47-3	0.00958	0.0138	9	0.101 *	-	1	0.0188	0.00394	0.0318	10	0.00133	0.0460	0.00112	0.101
PFHxA	92612-52-7	0.00487	0.0031	12	0.234 **	0.204	3	0.0508	0.00495	0.122	15	0.00154	0.134	0.0006	0.459
PFHpA	120885-29-2	0.00565	0.00436	11	-	-	-	0.00565	0.00495	0.00436	11	0.000681	0.00915	0.0006	0.0157
PFOA	45285-51-6	0.0134	0.0160	43	0.341	0.577	4	0.0413	0.00954	0.175	47	0.0018	0.0435	0.0000483	1.21
PFNA	72007-68-2	0.0403	0.0238	25	0.265	0.323	3	0.0643	0.0372	0.115	28	0.0117	0.0892	0.0033	0.632
PFDA	73829-36-4	0.132	0.250	28	0.815	1.05	4	0.218	0.0655	0.462	32	0.017	0.522	0.00585	2.36
PFUnDA	196859-54-8	0.278	0.545	27	1.16	1.17	4	0.392	0.135	0.697	31	0.0291	1.01	0.0126	2.81
PFDoDA	171978-95-3	0.499	0.660	26	1.76	1.37	3	0.628	0.307	0.822	29	0.0825	2.30	0.0193	2.78
PFTrDA	862374-87-6	0.214	-	1	5.87	-	1	3.04	3.04	4.00	2	0.78	5.31	0.214	5.87
PFTeDA	365971-87-5	0.121	-	1	4.70	-	1	2.41	2.41	3.24	2	0.579	4.24	0.121	4.70
PFHxDA	67905-19-5	0.0814	-	1	2.09	-	1	1.08	1.08	1.42	2	0.282	1.89	0.0814	2.09
Sulfonyl Compounds - OECD Structure Category 200															
Sulfonic Acids - OECD Structure Category 202															
PFBS	375-73-5	0.0783	0.113	23	0.444	0.393	3	0.120	0.0459	0.194	26	0.00316	0.365	0.0006	0.775
PFHxS	355-46-4	0.437	1.52	27	0.571	0.522	3	0.450	0.126	1.45	30	0.0144	0.580	0.00292	8.02
PFHpS	375-92-8	0.159	0.180	3	0.425	-	1	0.225	0.211	0.198	4	0.0542	0.408	0.0535	0.425
PFOS	1763-23-1	0.128	0.262	57	0.898 **	0.295	3	0.167	0.0587	0.311	60	0.0111	0.312	0.00073	1.92
PFDS	335-77-3	0.319	0.377	7	1.62 **	1.2	3	0.710	0.390	0.899	10	0.0467	1.56	0.0221	2.91
Cl-PFOS	1651215-26-7	0.0651	-	1	1.31	-	1	0.688	0.688	0.881	2	0.19	1.19	0.0651	1.31
Sulfonamides - OECD Structure Category: 203.01															
FOSA	754-91-6	0.184	0.346	4	-	-	-	0.184	0.0164	0.346	4	0.00333	0.500	0.00224	0.703
N-EtFOSE	1691-99-2	0.0638	0.0347	2	-	-	-	0.0638	0.0638	0.0347	2	0.0441	0.0834	0.0392	0.0884
Fluorotelomer related compounds - OECD Structure Category 400															
n:2 fluorotelomer alcohol, phosphate esters (PAPs) - OECD Structure Category: 402.04															
6:2 diPAP	57677-95-9	0.0384	-	1	-	-	-	0.0384	0.0384	-	1	-	-	-	-
Fluorotelomer sulfonate - OECD Structure Category: 402.07															
6:2 FTSA	27619-97-2	0.318	0.308	7	9.47 **	10.7	3	3.06	0.493	6.71	10	0.0243	6.49	0.0179	21.7
8:2 FTSA	39108-34-4	0.448	-	1	23.9	-	1	12.2	12.2	16.6	2	2.80	21.6	0.448	23.9
10:2 FTSA	120226-60-0	-	-	-	5.11	-	1	5.11	5.11	-	1	-	-	-	-
12:2 FTSA	1034143-66-2	-	-	-	3.61	-	1	3.61	3.61	-	1	-	-	-	-

* Signicantly different, α=0.05, one-sample t-test (t.test R-Statistics)

** Significantly different, α=0.05,Welch two-sample t-test (t.test R-Statistics)

## Data Availability

Data in Supplementary Materials.
