# Supplementary material for: Review of per- and polyfluoroalkyl substances (PFAS) bioaccumulation in earthworms
Source: Environ Adv. Author manuscript; Available in PMC 2024 Mar 13. (PMC10936581; doi:10.1016/j.envadv.2022.100335)
Supplement: Supplement2 [file NIHMS1953608-supplement-Supplement2.docx]

**Supplementary Data**

Per- and Poly-fluoroalkyl Substances (PFAS) Bioaccumulation in Earthworms

Lawrence P. Burkhard^a^ and Lauren K. Votava^b^

^a^ Great Lakes Toxicology and Ecology Division, Center for Computational Toxicology and Exposure (CCTE), Office of Research and Development, U.S. Environmental Protection Agency, 6201 Congdon Blvd, Duluth, MN 55804 USA.

^b^ Oak Ridge Associated Universities Student Services Contractor to U.S. Environmental Protection Agency, 6201 Congdon Blvd, Duluth, MN 55804 USA

Table of contents. 2

Table S1. PFAS OECD structure category, name, abbreviation, CAS number,

and molecular formula. 3

Table S2. Literature search terms 5

Table S3. Comparison of mean BSAFs (kg-OC/kg-ww) by species 11

Table S4. Comparison of mean BSAFs (kg-OC/kg-ww) study qualities 13

Table S5. Comparison of mean BSAFs (kg-OC/kg-ww) measured in the laboratory

and field settings 15

Table S6. Uptake and elimination rates for PFAS by oligochaetes 17

Table S7. Soil properties reported by study 19

Distribution plot of BSAFs and plot of BSAF (kg-OC/kg-ww) vs concentration in soil (mg/kg-OC).

Figure S1A: PFBA 20

Figure S1B: PFPeA 21

Figure S1C: PFHxA 22

Figure S1D: PFHpA 23

Figure S1E: PFOA 24

Figure S1F: PFNA 25

Figure S1G: PFDA 26

Figure S1H: PFUnDA 27

Figure S1I: PFDoDA 28

Figure S1J: PFBS 29

Figure S1K: PFHxS 30

Figure S1L: PFOS 31

Figure S1M: PFDS 32

References 33

| **Table S1. PFAS OECD structure category, name, abbreviation, CAS number, and molecular formula.** | | | |
| --- | --- | --- | --- |
| **Name** | **Abbreviation** | **CAS #** | **Molecular formula** |
| **Carbonyl Compounds - OECD Structure Category 100** | | | |
| **Carboxylic acids - OECD Structure Category 102 - perfluoroalkyl carboxylic acids (PFCAs), their salts and esters** | | | |
| Perfluorobutanoic acid | PFBA | 45048-62-2 | C_4_HF_7_O_2_ |
| Perfluoropentanoic acid | PFPeA | 45167-47-3 | C_5_HF_9_O_2_ |
| Perfluorohexanoic acid | PFHxA | 92612-52-7 | C_6_HF_11_O_2_ |
| Perfluoroheptanoic acid | PFHpA | 120885-29-2 | C_7_HF_13_O_2_ |
| Perfluorooctanoic acid | PFOA | 45285-51-6 | C_8_HF_15_O_2_ |
| Perfluorononanoic acid | PFNA | 72007-68-2 | C_9_HF_17_O_2_ |
| Perfluorodecanoic acid | PFDA | 73829-36-4 | C_10_HF_19_O_2_ |
| Perfluoroundecanoic acid | PFUnDA | 196859-54-8 | C_11_HF_21_O_2_ |
| Perfluorododecanoic acid | PFDoDA | 171978-95-3 | C_12_HF_23_O_2_ |
| Perfluorotridecanoic acid | PFTrDA | 862374-87-6 | C_13_HF_25_O_2_ |
| Perfluorotetradecanoic acid | PFTeDA | 365971-87-5 | C_14_HF_27_O_2_ |
| Perfluoropentadecanoic acid | PFPeDA | 1002-84-2 | C_15_HF_29_O_2_ |
| Perfluorohexadecanoic acid | PFHxDA | 67905-19-5 | C_16_HF_31_O_2_ |
| Perfluorooctadecanoic acid | PFOcDA | 16517-11-6 | C_18_HF_35_O_2_ |
| **Carboxylic acids - OECD Structure Category 103.01 - perfluoroalkyl carbonyl amides / amido ethanols and other alcohols** | | | |
| (Dimethyl{3-[(perfluorooctanoyl)amino]propyl}ammonio)acetate | PFOAB | 90179-39-8 | C_15_H_15_F_15_N_2_O_3_ |
| Perfluorooctaneamido ammonium | PFOAAmS | 45305-66-6 | C_14_H_16_F_15_N_2_O |
| **Sulfonyl Compounds - OECD Structure Category 200** | | | |
| **Sulfonic Acids - OECD Structure Category 202 - perfluoroalkane sulfonic acids (PFSAs), their salts and esters** | | | |
| Perfluorobutane sulfonic acid | PFBS | 375-73-5 | C_4_HF_9_O_3_S |
| Perfluoropentanesulfonic acid | PFPeS | 2706-91-4 | C_5_HF_11_O_3_S |
| Perfluorohexane sulfonic acid | PFHxS | 355-46-4 | C_6_F_13_O_3_S |
| Perfluoroheptane sulfonic acid | PFHpS | 375-92-8 | C_7_HF_15_O_3_S |
| 1-Chloroperfluorooctanesulfonic acid | Cl-PFOS | 1651215-26-7 | C_8_HClF_16_O_3_S |
| Perfluorooctane sulfonic acid | PFOS | 1763-23-1 | C_8_HF_17_O_3_S |
| Branched - Perfluorooctane sulfonic acid | br-PFOS | -- | C_8_HF_17_O_3_S |
| Perfluorononanesulfonic acid | PFNS | 68259-12-1 | C_9_HF_19_O_3_S |
| Perfluorodecane sulfonic acid | PFDS | 335-77-3 | C_10_HF_21_O_3_S |
| Perfluorododecanesulfonate | PFDoDS | 79780-39-5 | C_12_F_25_O_3_S |
| **Sulfonic Acids - OECD Structure Category 203.01 - perfluoroalkane sulfonyl amides/amido ethanols (xFASA/Es) and other alcohols** | | | |
| {[(Perfluorooctyl)sulfonyl]amino}-3-betaine | PFOSB | 75046-16-1 | C_15_H_15_F_17_N_2_O_4_S |
| 3-(Perfluorooctanesulfonamido)-N,N,N-trimethylpropan-1-aminium | PFOSAmS | 70225-25-1 | C_14_H_16_F_17_N_2_O_2_S |
| Perfluorooctane sulfonamide | PFOSA | 754-91-6 | C_8_H_2_F_17_N_2_O_2_S |
| N-methyl perfluorooctane sulfonamide | MeFOSA | 31506-32-8 | C_9_H_4_F_17_NO_2_S |
| 6:2 Fluorotelomer sulfonamide betaine | 6:2 FTAB | 34455-29-3 | C_15_H_19_F_13_N_2_O_4_S |
| 8:2 Fluorotelomer sulfonamide betaine | 8:2 FTAB | 34455-21-5 | C_17_H_19_F_17_N_2_O_4_S |
| 10:2 Fluorotelomer sulfonamide betaine | 10:2 FTAB | 34455-35-1 | C_19_H_19_F_21_N_2_O_4_S |
| 12:2 Fluorotelomer sulfonamide betaine | 12:2 FTAB | 278598-45-1 | C_21_H_19_F_25_N_2_O_4_S |
| 2-Perfluorooctylsulfonyl-N-ethylaminoethyl alcohol | N-EtFose | 1691-99-2 | C_12_H_10_F_17_NO_3_S |
| 7:3 Fluorotelomer betaine | 7:3 FtB | 171184-15-9 | C_14_H_14_F_15_NO_2_ |
| 9:3 Fluorotelomer betaine | 9:3 FtB | 171184-16-0 | C_16_H_14_F_19_NO_2_ |
| 11:3 Fluorotelomer betaine | 11:3 FtB | 171184-17-1 | C_18_H_14_F_23_NO_2_ |
| 13:3 Fluorotelomer betaine | 13:3 FtB | 1513864-13-5 | C_20_H_14_F_27_NO_2_ |
| 5:1:2 Fluorotelomer betaine | 5:1:2 FtB | 171184-02-4 | C_12_H_13_F_12_NO_2_ |
| 7:1:2 Fluorotelomer betaine | 7:1:2 FtB | 171184-03-5 | C_14_H_13_F_16_NO_2_ |
| 9:1:2 Fluorotelomer betaine | 9:1:2 FtB | 171184-04-6 | C_16_H_13_F_20_NO_2_ |
| 11:1:2 Fluorotelomer betaine | 11:1:2 FtB | 171184-05-7 | C_18_H_13_F_24_NO_2_ |
| 13:1:2 Fluorotelomer betaine | 13:1:2 FtB | 1513864-14-6 | C_20_H_14_F_28_NO_2_ |
| **Fluorotelomer related compounds - OECD Structure Category 400** | | | |
| **Fluorotelomer - OECD Structure Category: 402.04 - n:2 fluorotelomer olefins (n:2 FTOs)** |  |  |  |
| 10:2 Fluorotelomer alcohol | 10:2 FTOH | 865-86-1 | C_12_H_5_F_21_O |
| **Fluorotelomer - OECD Structure Category: 402.04 - n:2 fluorotelomer alcohol, phosphate esters (PAPs)** | |  |  |
| Bis(3,3,4,4,5,5,6,6,7,7,8,8,8-tridecafluorooctyl) hydrogen phosphate | 6:2 diPAP | 57677-95-9 | C_16_H_9_F_26_O_4_P |
| Bis(3,3,4,4,5,5,6,6,7,7,8,8,9,9,10,10,10-heptadecafluorodecyl) hydrogen phosphate | 8:2 diPAP | 678-41-1 | C_20_H_9_F_34_O_4_P |
| **Fluorotelomer - OECD Structure Category: 402.07 - Fluorotelomer sulfonate** | | | |
| 4:2 Fluorotelomer sulfonic acid | 4:2 FTS | 757124-72-4 | C_6_H_5_F_9_O_3_S |
| 6:2 Fluorotelomer sulfonic acid | 6:2 FTS | 27619-97-2 | C_8_H_4_F_13_O_3_S |
| 8:2 Fluorotelomer sulfonic acid | 8:2 FTS | 39108-34-4 | C_10_H_5_F_17_O_3_S |
| 10:2 Fluorotelomer sulfonic acid | 10:2 FTS | 120226-60-0 | C_12_H_5_F_21_O_3_S |
| 12:2 Fluorotelomer sulfonic acid | 12:2 FTS | 149246-64-0 | C_14_H_5_F_25_O_3_S |

| **Table S2. Literature Search Terms** | | |
| --- | --- | --- |
| **List of target PFAS for ECOTOX PFAS literature search conducted April 2018** | | |
| **PREFERRED_NAME** | **CASRN** | **SOURCE*** |
| 1,1,1,2,2,3,3,4,4,5,5,6,6,7,7,8,8-Heptadecafluoro-8-iodooctane | 507-63-1 | OECD |
| 1,1,1,2,2,3,3,4,4,5,5,6,6-Tridecafluoro-8-iodooctane | 2043-57-4 | OECD |
| 1,1,2,2,3,3,4,4,5,5,6,6,7,7,8,8,8-Heptadecafluoro-1-octanesulfonyl fluoride | 307-35-7 | OECD |
| 1,2,2,3,3,4,5,5,6,6-Decafluoro-4-(1,1,2,2,2-pentafluoroethyl)cyclohexanesulfonic acid, Potassium salt (1:1) | 33-52-0 | OECD |
| 10:1 Fluorotelomer alcohol | 307-46-0 | EPA |
| 10:2 Fluorotelomer acrylate | 17741-60-5 | EPA |
| 10:2 Fluorotelomer alcohol | 865-86-1 | EPA |
| 10:2 Fluorotelomer methacrylate | 2144-54-9 | EPA |
| 10:2 Fluorotelomer sulfonamide betaine | 34455-35-1 | EPA |
| 11:1 Fluorotelomer alcohol | 423-65-4 | EPA |
| 12:2 Fluorotelomer sulfonamido betaine | 278598-45-1 | EPA |
| 2-(N-Ethyl-perfluorooctanesulfonamido)acetate | 909405-49-8 | EPA |
| 2-(N-Ethylperfluorooctanesulfonamido)acetic acid | 2991-50-6 | EPA |
| 2-(N-Methylperfluorooctanesulfonamido)acetate | 909405-48-7 | EPA |
| 2-(N-Methylperfluorooctanesulfonamido)acetic acid | 2355-31-9 | EPA |
| 2H,2H,3H,3H-Perfluorooctanoic acid | 914637-49-3 | EPA |
| 2H-Perfluoro-2-decenoic acid | 70887-84-2 | EPA |
| 2H-Perfluoro-2-octenoic acid | 70887-88-6 | EPA |
| 2-Perfluorodecyl ethanoic acid | 53826-13-4 | EPA |
| 2-Perfluorohexyl ethanoic acid | 53826-12-3 | EPA |
| 2-Perfluorooctyl ethanoic acid | 27854-31-5 | EPA |
| 3,3,4,4,5,5,6,6,6-Nonafluorohexanoic acid | 70887-89-7 | Wang et al. 2011 |
| 3,4,4,5,5,6,6,6-Octafluoro-2-hexenoic acid | 70887-90-0 | Wang et al. 2011 |
| 3,4,4,5,5,6,6,7,7,8,8,9,9,10,10,11,11,12,12,12-Eicosafluoro-2-dodecenoic acid | 70887-94-4 | Buck et al. 2011 |
| 3-[[(1,1,2,2,3,3,4,4,5,5,6,6,7,7,8,8,8-Heptadecafluorooctyl)sulfonyl]amino]-N,N,N-trimethyl-1-propanaminium iodide (1:1) | 1652-63-7 | OECD |
| 3-Perfluoroheptylpropanoic acid | 812-70-4 | EPA |
| 4,8-Dioxa-3H-perfluorononanoic acid | 919005-14-4 | EPA |
| 4:2 Fluorotelomer alcohol | 2043-47-2 | EPA |
| 4:2 Fluorotelomer sulfonate | 414911-30-1 | EPA |
| 4:2 Fluorotelomer sulfonic acid | 757124-72-4 | EPA |
| 4:2 Fluorotelomer thioether amido sulfonate | 1432486-88-8 | EPA |
| 5:1 Fluorotelomer alcohol | 423-46-1 | EPA |
| 5:1:2 Fluorotelomer betaine | 171184-02-4 | EPA |
| 5:3 Fluorotelomer betaine | 171184-14-8 | EPA |
| 6:1 Fluorotelomer alcohol | 375-82-6 | EPA |
| 6:2 Fluorotelomer acrylate | 17527-29-6 | EPA |
| 6:2 Fluorotelomer alcohol | 647-42-7 | EPA |
| 6:2 Fluorotelomer methacrylate | 2144-53-8 | EPA |
| 6:2 Fluorotelomer phosphate diester | 57677-95-9 | EPA |
| 6:2 Fluorotelomer phosphate monoester | 57678-01-0 | EPA |
| 6:2 Fluorotelomer sulfonamide betaine | 34455-29-3 | EPA |
| 6:2 Fluorotelomer sulfonamido N,N-dimethyl amine | 1383438-86-5 | EPA |
| 6:2 Fluorotelomer sulfonate | 425670-75-3 | EPA |
| 6:2 Fluorotelomer sulfonic acid | 27619-97-2 | EPA |
| 6:2 Fluorotelomer thioether amido sulfonate | 88992-47-6 | EPA |
| 6:2 Fluorotelomer thioether hydroxyammonium | 88992-46-5 | EPA |
| 6:2/8:2 Fluorotelomer phosphate diester | 943913-15-3 | EPA |
| 7:1 Fluorotelomer alcohol | 307-30-2 | EPA |
| 7:1:2 Fluorotelomer betaine | 171184-03-5 | EPA |
| 7:2 sFluorotelomer alcohol | 24015-83-6 | EPA |
| 7:3 Fluorotelomer betaine | 171184-15-9 | EPA |
| 8:1 Fluorotelomer alcohol | 423-56-3 | EPA |
| 8:2 Fluorotelomer acrylate | 27905-45-9 | EPA |
| 8:2 Fluorotelomer alcohol | 678-39-7 | EPA |
| 8:2 Fluorotelomer dihydrogen phosphate | 57678-03-2 | EPA |
| 8:2 Fluorotelomer methacrylate | 1996-88-9 | EPA |
| 8:2 Fluorotelomer phosphate diester | 678-41-1 | EPA |
| 8:2 Fluorotelomer sulfonamido N,N-dimethyl amine ion | 1383438-87-6 | EPA |
| 8:2 Fluorotelomer sulfonate | 481071-78-7 | EPA |
| 8:2 Fluorotelomer sulfonic acid | 39108-34-4 | EPA |
| 8:2 Fluorotelomer thioether amido sulfonate | 1383439-45-9 | EPA |
| 8:2 Fuorotelomer sulfonamide betaine | 34455-21-5 | EPA |
| 8-Fluorosulfonylperfluoro(2,5-dimethyl-3,6-dioxaoctanoyl) fluoride | 4089-58-1 | EPA |
| 9:1 Fluorotelomer alcohol | 307-37-9 | EPA |
| 9:1:2 Fluorotelomer betaine | 171184-04-6 | EPA |
| 9:3 Fluorotelomer betaine | 171184-16-0 | EPA |
| Ammonium 2-(N-ethylperfluorooctanesulfonamido)acetate | 2991-52-8 | EPA |
| Ammonium 4,8-dioxa-3H-perfluorononanoate | 958445-44-8 | EPA |
| Ammonium perfluoro-2-methyl-3-oxahexanoate | 62037-80-3 | EPA |
| Ammonium perfluorobutanesulfonate | 68259-10-9 | EPA |
| Ammonium perfluorodecanesulfonate | 67906-42-7 | EPA |
| Ammonium perfluorodecanoate | 3108-42-7 | EPA |
| Ammonium perfluoroheptanesulfonate | 68259-07-4 | EPA |
| Ammonium perfluoroheptanoate | 6130-43-4 | EPA |
| Ammonium perfluorohexanesulfonate | 68259-08-5 | EPA |
| Ammonium perfluorohexanoate | 21615-47-4 | EPA |
| Ammonium perfluorononanesulfonate | 17202-41-4 | EPA |
| Ammonium perfluorononanoate | 4149-60-4 | EPA |
| Ammonium perfluorooctanesulfonate | 29081-56-9 | EPA |
| Ammonium perfluorooctanoate | 3825-26-1 | EPA |
| Ammonium perfluoropentanesulfonate | 68259-09-6 | EPA |
| Ammonium perfluoropentanoate | 68259-11-0 | EPA |
| Ammonium perfluoroundecanoate | 3658-63-7 | EPA |
| Difluoro(perfluoromethoxy)acetic acid | 674-13-5 | EPA |
| Difluoro(perfluoropropoxy)acetic acid | 919005-50-8 | EPA |
| gamma-omega-Perfluoroalkyl iodides C8-C14 | 85995-91-1 | OECD |
| Lithium perfluoroheptanesulfonate | 117806-54-9 | EPA |
| Lithium perfluorohexanesulfonate | 55120-77-9 | EPA |
| Lithium perfluorooctanesulfonate | 29457-72-5 | EPA |
| N,N,N-triethylethanaminium 1,1,2,2,3,3,4,4,5,5,6,6,7,7,8,8,8-heptadecafluoro-1-octanesulfonate (1:1) | 56773-42-3 | OECD |
| N,N'-[Phosphinicobis(oxy-2,1-ethanediyl)]bis[N-ethyl-1,1,2,2,3,3,4,4,5,5,6,6,7,7,8,8,8-heptadecafluoro-1-octanesulfonamide ammonium salt (1:1) | 30381-98-7 | OECD |
| N-Ethyl-N-(2-hydroxyethyl)perfluorooctanesulfonamide | 1691-99-2 | EPA |
| N-Ethylperfluorooctanesulfonamide | 4151-50-2 | EPA |
| N-Methyl-N-(2-hydroxyethyl)perfluorooctanesulfonamide | 24448-09-7 | EPA |
| N-Methylperfluorooctanesulfonamide | 31506-32-8 | EPA |
| P-(1,1,2,2,3,3,4,4,5,5,6,6,6-Tridecafluorohexyl)phosphonic acid | 40143-76-8 | Buck et al. 2011 |
| P-(1,1,2,2,3,3,4,4,5,5,6,6,7,7,8,8,8-Heptadecafluorooctyl)-P-(1,1,2,2,3,3,4,4,5,5,6,6,6-tridecafluorohexyl)phosphinic acid | 610800-34-5 | Buck et al. 2011 |
| P-(1,1,2,2,3,3,4,4,5,5,6,6,7,7,8,8,8-Heptadecafluorooctyl)phosphonic acid | 40143-78-0 | Buck et al. 2011 |
| P-(1,1,2,2,3,3,4,4,5,5,6,6,7,7,8,8,9,9,10,10,10-Heneicosafluorodecyl)phosphonic acid | 52299-26-0 | Buck et al. 2011 |
| P,P-Bis(1,1,2,2,3,3,4,4,5,5,6,6,6-tridecafluorohexyl)phosphinic acid | 40143-77-9 | Buck et al. 2011 |
| P,P-Bis(1,1,2,2,3,3,4,4,5,5,6,6,7,7,8,8,8-heptadecafluorooctyl)phosphinic acid | 40143-79-1 | Buck et al. 2011 |
| Perfluoro(2,5,8,10-tetramethyl-3,6,9-trioxaundecanoic) acid | 1212077-14-9 | EPA |
| Perfluoro(2,5,8,11,14-pentamethyl-3,6,9,12,15-pentaoxaoctadecanoic) acid | 52481-85-3 | EPA |
| Perfluoro-(2,5,8-trimethyl-3,6,9-trioxadodecanoic)acid | 65294-16-8 | EPA |
| Perfluoro(4-methoxybutanoic) acid | 863090-89-5 | EPA |
| Perfluoro(4-methyl-3,6-dioxaoct-7-ene)sulfonyl fluoride | 16090-14-5 | EPA |
| Perfluoro-2-(perfluorobutoxy)-2-(perfluoromethyl)propanoic acid | NOCAS_892364 | EPA |
| Perfluoro-2-(perfluoromethoxy)propanoic acid | 13140-29-9 | EPA |
| Perfluoro-2-(perfluoropropoxy)-2-(perfluoromethyl)propanoic acid | NOCAS_892361 | EPA |
| Perfluoro-2,5-dimethyl-3,6-dioxanonanoic acid | 13252-14-7 | EPA |
| Perfluoro-2-[(perfluoropentyl)oxy]propanoic acid | 504435-11-4 | EPA |
| Perfluoro-2-{[perfluoro-3-(perfluoroethoxy)-2-propanyl]oxy}ethanesulfonic acid | 749836-20-2 | EPA |
| Perfluoro-2-methyl-3-oxahexanoic acid | 13252-13-6 | EPA |
| Perfluoro-3-(1H-perfluoroethoxy)propane | 3330-15-2 | EPA |
| Perfluoro-3,5,7,9,11-pentaoxadodecanoic acid | 39492-91-6 | EPA |
| Perﬂuoro-3,5,7,9-butaoxadecanoic acid | 39492-90-5 | EPA |
| Perfluoro-3,5,7-trioxaoctanoic acid | 39492-89-2 | EPA |
| Perﬂuoro-3,5-dioxahexanoic acid | 39492-88-1 | EPA |
| Perfluoro-3,6,9-trioxadecanoic acid | 151772-59-7 | EPA |
| Perfluoro-3,6,9-trioxatridecanoic acid | 330562-41-9 | EPA |
| Perfluoro-3,6-dioxa-4-methyl-7-octene-1-sulfonic acid | 29311-67-9 | EPA |
| Perfluoro-3,6-dioxadecanoic acid | 137780-69-9 | EPA |
| Perfluoro-3,6-dioxaheptanoic acid | 151772-58-6 | EPA |
| Perfluoro-3-ethoxypropanoic acid | 377-76-4 | EPA |
| Perfluoro-3-methoxypropanoic acid | 377-73-1 | EPA |
| Perfluoro-4-(perfluoroethyl)cyclohexylsulfonate | 80988-54-1 | EPA |
| Perfluoro-4-(perfluoroethyl)cyclohexylsulfonic acid | 646-83-3 | EPA |
| Perfluoro-4-isopropoxybutanoic acid | 801212-59-9 | EPA |
| Perfluorobutane sulfonamide amino carboxylates | NOCAS_892534 | EPA |
| Perfluorobutane sulfonamido amine | 68555-77-1 | EPA |
| Perfluorobutanesulfonate | 45187-15-3 | EPA |
| Perfluorobutanesulfonic acid | 375-73-5 | EPA |
| Perfluorobutanoate | 45048-62-2 | EPA |
| Perfluorobutanoic acid | 375-22-4 | EPA |
| Perfluorodecanesulfonate | 126105-34-8 | EPA |
| Perfluorodecanesulfonic acid | 335-77-3 | EPA |
| Perfluorodecanoate | 73829-36-4 | EPA |
| Perfluorodecanoic acid | 335-76-2 | EPA |
| Perfluorododecanoate | 171978-95-3 | EPA |
| Perfluorododecanoic acid | 307-55-1 | EPA |
| Perfluoroethanesulfonic acid | 354-88-1 | EPA |
| Perfluoroheptane sulfonamide amino carboxylates | NOCAS_892546 | EPA |
| Perfluoroheptane sulfonamido amine | 67584-54-7 | EPA |
| Perfluoroheptanesulfonate | 146689-46-5 | EPA |
| Perfluoroheptanesulfonic acid | 375-92-8 | EPA |
| Perfluoroheptanoate | 120885-29-2 | EPA |
| Perfluoroheptanoic acid | 375-85-9 | EPA |
| Perfluorohexadecanoic acid | 67905-19-5 | EPA |
| Perfluorohexane sulfonamide amino carboxylates | NOCAS_892548 | EPA |
| Perfluorohexane sulfonamido amine | 50598-28-2 | EPA |
| Perfluorohexanesulfonate | 108427-53-8 | EPA |
| Perfluorohexanesulfonic acid | 355-46-4 | EPA |
| Perfluorohexanoate | 92612-52-7 | EPA |
| Perfluorohexanoic acid | 307-24-4 | EPA |
| Perfluorononanesulfonate | 474511-07-4 | EPA |
| Perfluorononanesulfonic acid | 68259-12-1 | EPA |
| Perfluorononanoate | 72007-68-2 | EPA |
| Perfluorononanoic acid | 375-95-1 | EPA |
| Perfluorooctadecanoic acid | 16517-11-6 | EPA |
| Perfluorooctane sulfonamide amino carboxylates | NOCAS_892552 | EPA |
| Perfluorooctane sulfonamido amine | 13417-01-1 | EPA |
| Perfluorooctanesulfonamide | 754-91-6 | EPA |
| Perfluorooctanesulfonamido ethanol | 10116-92-4 | EPA |
| Perfluorooctanesulfonate | 45298-90-6 | EPA |
| Perfluorooctanesulfonic acid | 1763-23-1 | EPA |
| Perfluorooctanoate ion(1-) | 45285-51-6 | EPA |
| Perfluorooctanoic acid | 335-67-1 | EPA |
| Perfluoropentane sulfonamide amino carboxylates | NOCAS_892553 | EPA |
| Perfluoropentane sulfonamido amine | 68555-78-2 | EPA |
| Perfluoropentanesulfonate | 175905-36-9 | EPA |
| Perfluoropentanesulfonic acid | 2706-91-4 | EPA |
| Perfluoropentanoate | 45167-47-3 | EPA |
| Perfluoropentanoic acid | 2706-90-3 | EPA |
| Perfluoropropanesulfonic acid | 423-41-6 | EPA |
| Perfluoropropanoate | 44864-55-3 | EPA |
| Perfluoropropanoic acid | 422-64-0 | EPA |
| Perfluorosulfonic acid, PTFE copolymer | 66796-30-3 | EPA |
| Perfluorotetradecanoate | 365971-87-5 | EPA |
| Perfluorotetradecanoic acid | 376-06-7 | EPA |
| Perfluorotridecanoate | 862374-87-6 | EPA |
| Perfluorotridecanoic acid | 72629-94-8 | EPA |
| Perfluoroundecanoate | 196859-54-8 | EPA |
| Perfluoroundecanoic acid | 2058-94-8 | EPA |
| Potassium 11-chloroeicosafluoro-3-oxaundecane-1-sulfonate | 83329-89-9 | EPA |
| Potassium 2-(N-ethylperfluorooctanesulfonamido)acetate | 2991-51-7 | EPA |
| Potassium 9-chlorohexadecafluoro-3-oxanonane-1-sulfonate | 73606-19-6 | EPA |
| Potassium perfluorobutanesulfonate | 29420-49-3 | EPA |
| Potassium perfluoroheptanesulfonate | 60270-55-5 | EPA |
| Potassium perfluorohexanesulfonate | 3871-99-6 | EPA |
| Potassium perfluorooctanesulfonate | 2795-39-3 | EPA |
| Potassium perfluorooctanoate | 2395-00-8 | EPA |
| Potassium perfluoropentanesulfonate | 3872-25-1 | EPA |
| Silver perfluorobutanoate | 3794-64-7 | EPA |
| Silver perfluorooctanoate | 335-93-3 | EPA |
| Sodium 2-(N-ethylperfluorooctanesulfonamido)acetate | 3871-50-9 | EPA |
| Sodium 4,8-dioxa-3H-perfluorononanoate | NOCAS_892452 | EPA |
| Sodium perfluorobutanoate | 2218-54-4 | EPA |
| Sodium perfluorodecanesulfonate | 2806-15-7 | EPA |
| Sodium perfluorodecanoate | 3830-45-3 | EPA |
| Sodium perfluoroheptanoate | 20109-59-5 | EPA |
| Sodium perfluorohexanesulfonate | 82382-12-5 | EPA |
| Sodium perfluorohexanoate | 2923-26-4 | EPA |
| Sodium perfluorooctanesulfonate | 4021-47-0 | EPA |
| Sodium perfluorooctanoate | 335-95-5 | EPA |
| Sodium perfluoropentanoate | 2706-89-0 | EPA |
| Trifluoroacetate | 14477-72-6 | EPA |
| Trifluoroacetic acid | 76-05-1 | EPA |
| *Sources: |  |  |
| EPA = Initial EPA PFAS Research list obtained from the CompTox Chemicals Dashboard, April 2018; | |  |
| [OECD = OECD Lists of PFOS, PFAS, PFOA, PFCA, Related Compounds and Chemicals That May Degrade to PFCA, http://www.oecd.org/officialdocuments/publicdisplaydocumentpdf/?doclanguage=en&cote=env/jm/mono(2006)15;](http://www.oecd.org/officialdocuments/publicdisplaydocumentpdf/?doclanguage=en&cote=env/jm/mono(2006)15) | | |
| [Buck et al. 2011 = Buck et al. 2011. Perfluoroalkyl and polyfluoralkyl substances (PFASs) in the environment: terminology, classification, and origins, https://doi.org/10.1002/ieam.258;](https://doi.org/10.1002/ieam.258) | | |
| Wang, et al. 2011 = Wang et al. 2011. Using COSMOtherm to predict physiochemical properties of poly- and perfluorinated alkyl substances (PFASs), doi:10.1071/EN10143_AC (https://www.publish.csiro.au/en/acc/EN10143/EN10143_AC.pdf) | | |

| **Table S3.** Comparison of mean BSAFs (kg-OC/kg-ww) by species | | | | | | | | | | | | | | | | | | | | | | | | | | | | | | | | | | |
| --- | --- | --- | --- | --- | --- | --- | --- | --- | --- | --- | --- | --- | --- | --- | --- | --- | --- | --- | --- | --- | --- | --- | --- | --- | --- | --- | --- | --- | --- | --- | --- | --- | --- | --- |
|  |  | | ***Eisenia andrei*** | | | | | | | ***Lumbricus terrestris*** | | | | | | | | ***Eisenia fetida*** | | | | | | **MX **** | | |  | **ANOVA** | **TukeyHSD** | | | | | |
| **Chemical** | **CAS** | | **average** | | **median** | | **standard deviation** | **n** | | **average** | | **median** | | | **standard deviation** | | **n** | **average** | | **median** | | **standard deviation** | **n** | **average** | **median** | **standard deviation** | **n** | **p-value** | **ET-EA** | **LT-EA** | **LT-EF** | **MX-EA** | **MX-EF** | **LT-MX** |
| **Carbonyl Compounds - OECD Structure Category 100** | | | | | | | | | | | | | | | | | | | | | | | | | | | | | | | | | | |
| **Carboxylic acids - OECD Structure Category 102 - perfluoroalkyl carboxylic acids (PFCAs), their salts and esters** | | | | | | | | | | | | | | | | | | | | | | | | | | | | | | | | | | |
| PFBA | 45048-62-2 | | -- | | -- | | -- | 0 | | -- | | -- | | | -- | | 0 | 0.0488 | | 0.0229 | | 0.0814 | 7 | -- | -- | -- | 0 | -- | -- | -- | -- | -- | -- | -- |
| PFPeA | 45167-47-3 | | -- | | -- | | -- | 0 | | -- | | -- | | | -- | | 0 | 0.0188 | | 0.00394 | | 0.0318 | 10 | -- | -- | -- | 0 | -- | -- | -- | -- | -- | -- | -- |
| PFHxA | 92612-52-7 | | -- | | -- | | -- | 0 | | -- | | -- | | | -- | | 0 | 0.0093 | | 0.00488 | | 0.0162 | 13 | 0.321 | 0.321 | 0.196 | 2 | 0.000298 | -- | -- | -- | -- | 0.000298 | -- |
| PFHpA | 120885-29-2 | | -- | | -- | | -- | 0 | | -- | | -- | | | -- | | 0 | 0.00565 | | 0.00495 | | 0.00436 | 11 | -- | -- | -- | 0 | -- | -- | -- | -- | -- | -- | -- |
| PFOA | 45285-51-6 | | 0.0071 | | 0.00789 | | 0.00292 | 3 | | 0.0168 | | 0.0159 | | | 0.00403 | | 3 | 0.015 | | 0.00469 | | 0.0186 | 39 | 0.642 | 0.642 | 0.798 | 2 | 0.0214 | 0.999 | 0.916 | 0.744 | 0.0862 | 0.0144 | 0.263 |
| PFNA | 72007-68-2 | | -- | | -- | | -- | 0 | | 0.0225 | | 0.0225 | | | -- | | 1 | 0.0441 | | 0.0372 | | 0.0305 | 26 | 0.632 | 0.632 | -- | 1 | 0.0075 | -- | -- | 0.886 | -- | 0.00606 | 0.0257 |
| PFDA | 73829-36-4 | | 0.0381 | | 0.03 | | 0.0267 | 4 | | 0.027 | | 0.027 | | | -- | | 1 | 0.164 | | 0.0674 | | 0.274 | 25 | 1.35 | 1.35 | 1.43 | 2 | 0.0261 | 0.65 | 0.999 | 0.872 | 0.0205 | 0.041 | 0.116 |
| PFUnDA | 196859-54-8 | | 0.286 | | 0.286 | | 0.169 | 2 | | 0.07 | | 0.07 | | | -- | | 1 | 0.305 | | 0.126 | | 0.574 | 26 | 1.78 | 1.78 | 1.46 | 2 | 0.0615 | 0.832 | 0.824 | 0.973 | 0.518 | 0.0499 | 0.215 |
| PFDoDA | 171978-95-3 | | 0.663 | | 0.51 | | 0.455 | 4 | | 0.197 | | 0.197 | | | -- | | 1 | 0.548 | | 0.261 | | 0.777 | 23 | 2.78 | 2.78 | -- | 1 | 0.221 | 0.633 | 0.876 | 0.998 | 0.678 | 0.268 | 0.463 |
| PFTrDA | 862374-87-6 | | -- | | -- | | -- | 0 | | -- | | -- | | | -- | | 0 | 3.04 | | 3.04 | | 4 | 2 | -- | -- | -- | 0 | -- | -- | -- | -- | -- | -- | -- |
| PFTeDA | 365971-87-5 | | -- | | -- | | -- | 0 | | -- | | -- | | | -- | | 0 | 2.41 | | 2.41 | | 3.24 | 2 | -- | -- | -- | 0 | -- | -- | -- | -- | -- | -- | -- |
| PFPeDA | 1002-84-2 | | -- | | -- | | -- | 0 | | -- | | -- | | | -- | | 0 | -- | | -- | | -- | 0 | -- | -- | -- | 0 | -- | -- | -- | -- | -- | -- | -- |
| PFHxDA | 67905-19-5 | | -- | | -- | | -- | 0 | | -- | | -- | | | -- | | 0 | 1.08 | | 1.08 | | 1.42 | 2 | -- | -- | -- | 0 | -- | -- | -- | -- | -- | -- | -- |
| PFOcDA | 16517-11-6 | | -- | | -- | | -- | 0 | | -- | | -- | | | -- | | 0 | -- | | -- | | -- | 0 | -- | -- | -- | 0 | -- | -- | -- | -- | -- | -- | -- |
| **Carboxylic acids - OECD Structure Category 103.01 - perfluoroalkyl carbonyl amides / amido ethanols and other alcohols** | | | | | | | | | | | | | | | | | | | | | | | | | | | | | | | | | | |
| PFOAB | 90179-39-8 | | -- | | -- | | -- | 0 | | 0.0813 | | 0.0689 | | | 0.0361 | | 3 | -- | | -- | | -- | 0 | -- | -- | -- | 0 | -- | -- | -- | -- | -- | -- | -- |
| PFOAAmS | 45305-66-6 | | -- | | -- | | -- | 0 | | 0.0318 | | 0.0265 | | | 0.014 | | 3 | -- | | -- | | -- | 0 | -- | -- | -- | 0 | -- | -- | -- | -- | -- | -- | -- |
| **Sulfonyl Compounds - OECD Structure Category 200** | | | | | | | | | | | | | | | | | | | | | | | | | | | | | | | | | | |
| **Sulfonic Acids - OECD Structure Category 202 - perfluoroalkane sulfonic acids (PFSAs), their salts and esters** | | | | | | | | | | | | | | | | | | | | | | | | | | | | | | | | | | |
| PFBS | 375-73-5 | | 0.0114 | | 0.0114 | | 0.00134 | 2 | | -- | | -- | | | -- | | 0 | 0.0813 | | 0.0459 | | 0.115 | 22 | 0.66 | 0.66 | 0.163 | 2 | 0.0388 | 0.682 | -- | -- | 0.0516 | 0.0474 | -- |
| PFPeS | 2706-91-4 | | -- | | -- | | -- | 0 | | -- | | -- | | | -- | | 0 | 0.0721 | | 0.0721 | | -- | 1 | -- | -- | -- | 0 | -- | -- | -- | -- | -- | -- | -- |
| PFHxS | 355-46-4 | | 0.0156 | | 0.0156 | | 0.000477 | 2 | | -- | | -- | | | -- | | 0 | 0.454 | | 0.126 | | 1.55 | 26 | 0.832 | 0.832 | 0.365 | 2 | 0.0526 | 0.242 | -- | -- | 0.0418 | 0.176 | -- |
| PFHpS | 375-92-8 | | -- | | -- | | -- | 0 | | -- | | -- | | | -- | | 0 | 0.225 | | 0.211 | | 0.198 | 4 | -- | -- | -- | 0 | -- | -- | -- | -- | -- | -- | -- |
| Cl-PFOS | 1651215-26-7 | | -- | | -- | | -- | 0 | | -- | | -- | | | -- | | 0 | 0.688 | | 0.688 | | 0.881 | 2 | -- | -- | -- | 0 | -- | -- | -- | -- | -- | -- | -- |
| PFOS | 1763-23-1 | | 0.0548 | | 0.0353 | | 0.0353 | 7 | | 0.196 | | 0.196 | | | 0.0525 | | 2 | 0.158 | | 0.0576 | | 0.32 | 49 | 0.742 | 0.742 | 0.168 | 2 | 0.0691 | 0.989 | 0.611 | 0.635 | 0.0977 | 0.0804 | 0.803 |
| br-PFOS | -- | | -- | | -- | | -- | 0 | | -- | | -- | | | -- | | 0 | -- | | -- | | -- | 0 | -- | -- | -- | 0 | -- | -- | -- | -- | -- | -- | -- |
| PFNS | 68259-12-1 | | -- | | -- | | -- | 0 | | -- | | -- | | | -- | | 0 | 0.55 | | 0.55 | | 0.679 | 2 | -- | -- | -- | 0 | -- | -- | -- | -- | -- | -- | -- |
| PFDS | 335-77-3 | | -- | | -- | | -- | 0 | | -- | | -- | | | -- | | 0 | 0.456 | | 0.21 | | 0.52 | 8 | 1.73 | 1.73 | 1.67 | 2 | 0.155 | -- | -- | -- | -- | 0.155 | -- |
| PFDoDS | 79780-39-5 | | -- | | -- | | -- | 0 | | -- | | -- | | | -- | | 0 | 0.223 | | 0.223 | | -- | 1 | -- | -- | -- | 0 | -- | -- | -- | -- | -- | -- | -- |
| **Sulfonic Acids - OECD Structure Category 203.01 - perfluoroalkane sulfonyl amides/amido ethanols (xFASA/Es) and other alcohols** | | | | | | | | | | | | | | | | | | | | | | | | | | | | | | | | | | |
| PFOSB | 75046-16-1 | | -- | | -- | | -- | 0 | | 0.0477 | | 0.0424 | | | 0.0191 | | 3 | -- | | -- | | -- | 0 | -- | -- | -- | 0 | -- | -- | -- | -- | -- | -- | -- |
| PFOSAmS | 70225-25-1 | | -- | | -- | | -- | 0 | | 0.0265 | | 0.0212 | | | 0.014 | | 3 | -- | | -- | | -- | 0 | -- | -- | -- | 0 | -- | -- | -- | -- | -- | -- | -- |
| PFOSA | 754-91-6 | | -- | | -- | | -- | 0 | | -- | | -- | | | -- | | 0 | 0.184 | | 0.0164 | | 0.346 | 4 | -- | -- | -- | 0 | -- | -- | -- | -- | -- | -- | -- |
| MeFOSA | 31506-32-8 | | -- | | -- | | -- | 0 | | -- | | -- | | | -- | | 0 | 0.0896 | | 0.0896 | | -- | 1 | -- | -- | -- | 0 | -- | -- | -- | -- | -- | -- | -- |
| 6:2 FTAB | 34455-29-3 | | -- | | -- | | -- | 0 | | -- | | -- | | | -- | | 0 | 0.0644 | | 0.0109 | | 0.11 | 4 | -- | -- | -- | 0 | -- | -- | -- | -- | -- | -- | -- |
| 8:2 FTAB | 34455-21-5 | | -- | | -- | | -- | 0 | | -- | | -- | | | -- | | 0 | 0.0225 | | 0.026 | | 0.0109 | 3 | -- | -- | -- | 0 | -- | -- | -- | -- | -- | -- | -- |
| 10:2 FTAB | 34455-35-1 | | -- | | -- | | -- | 0 | | -- | | -- | | | -- | | 0 | 0.03 | | 0.03 | | -- | 1 | -- | -- | -- | 0 | -- | -- | -- | -- | -- | -- | -- |
| 12:2 FTAB | 278598-45-1 | | -- | | -- | | -- | 0 | | -- | | -- | | | -- | | 0 | 0.026 | | 0.026 | | -- | 1 | -- | -- | -- | 0 | -- | -- | -- | -- | -- | -- | -- |
| N-EtFose | 1691-99-2 | | -- | | -- | | -- | 0 | | -- | | -- | | | -- | | 0 | 0.0638 | | 0.0638 | | 0.0347 | 2 | -- | -- | -- | 0 | -- | -- | -- | -- | -- | -- | -- |
| 7:3 FtB | 171184-15-9 | | -- | | -- | | -- | 0 | | -- | | -- | | | -- | | 0 | 0.0323 | | 0.00419 | | 0.0497 | 3 | -- | -- | -- | 0 | -- | -- | -- | -- | -- | -- | -- |
| 9:3 FtB | 171184-16-0 | | -- | | -- | | -- | 0 | | -- | | -- | | | -- | | 0 | 0.0301 | | 0.014 | | 0.035 | 3 | -- | -- | -- | 0 | -- | -- | -- | -- | -- | -- | -- |
| 11:3 FtB | 171184-17-1 | | -- | | -- | | -- | 0 | | -- | | -- | | | -- | | 0 | 0.0453 | | 0.0232 | | 0.0525 | 3 | -- | -- | -- | 0 | -- | -- | -- | -- | -- | -- | -- |
| 13:3 FtB | 1513864-13-5 | | -- | | -- | | -- | 0 | | -- | | -- | | | -- | | 0 | 0.0746 | | 0.0121 | | 0.114 | 3 | -- | -- | -- | 0 | -- | -- | -- | -- | -- | -- | -- |
| 5:1:2 FtB | 171184-02-4 | | -- | | -- | | -- | 0 | | -- | | -- | | | -- | | 0 | 0.00945 | | 0.00279 | | 0.0117 | 3 | -- | -- | -- | 0 | -- | -- | -- | -- | -- | -- | -- |
| 7:1:2 FtB | 171184-03-5 | | -- | | -- | | -- | 0 | | -- | | -- | | | -- | | 0 | 0.0297 | | 0.00419 | | 0.0452 | 3 | -- | -- | -- | 0 | -- | -- | -- | -- | -- | -- | -- |
| 9:1:2 FtB | 171184-04-6 | | -- | | -- | | -- | 0 | | -- | | -- | | | -- | | 0 | 0.0272 | | 0.013 | | 0.0306 | 3 | -- | -- | -- | 0 | -- | -- | -- | -- | -- | -- | -- |
| 11:1:2 FtB | 171184-05-7 | | -- | | -- | | -- | 0 | | -- | | -- | | | -- | | 0 | 0.041 | | 0.0223 | | 0.0461 | 3 | -- | -- | -- | 0 | -- | -- | -- | -- | -- | -- | -- |
| 13:1:2 FtB | 1513864-14-6 | | -- | | -- | | -- | 0 | | -- | | -- | | | -- | | 0 | 0.0578 | | 0.0121 | | 0.0851 | 3 | -- | -- | -- | 0 | -- | -- | -- | -- | -- | -- | -- |
| **Fluorotelomer related compounds - OECD Structure Category 400** | | | | | | | | | | | | | | | | | | | | | | | | | | | | | | | | | | |
| **Fluorotelomer - OECD Structure Category: 402.04 - n:2 fluorotelomer olefins (n:2 FTOs)** | | | | | | | | | | | | | | | | | | | | | | | | | | | | | | | | | | |
| 10:2 FTOH | 865-86-1 | | -- | | -- | | -- | 0 | | -- | | -- | | | -- | | 0 | 0.0404 | | 0.0404 | | -- | 1 | -- | -- | -- | 0 | -- | -- | -- | -- | -- | -- | -- |
| **Fluorotelomer - OECD Structure Category: 402.04 - n:2 fluorotelomer alcohol, phosphate esters (PAPs)** | | | | | | | | | | | | | | | | | | | | | | | | | | | | | | | | | | |
| 6:2 diPAP | 57677-95-9 | | 0.0384 * | | 0.0384 | | -- | 1 | | -- | | -- | | | -- | | 0 | -- | | -- | | -- | 0 | -- | -- | -- | 0 | -- | -- | -- | -- | -- | -- | -- |
| 8:2 diPAP | 678-41-1 | | -- | | -- | | -- | 0 | | -- | | -- | | | -- | | 0 | -- | | -- | | -- | 0 | -- | -- | -- | 0 | -- | -- | -- | -- | -- | -- | -- |
| **Fluorotelomer - OECD Structure Category: 402.07 - Fluorotelomer sulfonate** | | | | | | | | | | | | | | | | | | | | | | | | | | | | | | | | | | |
| 4:2 FTS | 757124-72-4 | | -- | | -- | | -- | 0 | | -- | | -- | | | -- | | 0 | 0.00175 | | 0.00175 | | -- | 1 | -- | -- | -- | 0 | -- | -- | -- | -- | -- | -- | -- |
| 6:2 FTS | 27619-97-2 | | -- | | -- | | -- | 0 | | -- | | -- | | | -- | | 0 | 2.99 | | 0.392 | | 7.57 | 8 | 3.35 | 3.35 | 2.05 | 2 | 0.199 | -- | -- | -- | -- | 0.199 | -- |
| 8:2 FTS | 39108-34-4 | | -- | | -- | | -- | 0 | | -- | | -- | | | -- | | 0 | 12.2 | | 12.2 | | 16.6 | 2 | -- | -- | -- | 0 | -- | -- | -- | -- | -- | -- | -- |
| 10:2 FTS | 120226-60-0 | | -- | | -- | | -- | 0 | | -- | | -- | | | -- | | 0 | 5.11 | | 5.11 | | -- | 1 | -- | -- | -- | 0 | -- | -- | -- | -- | -- | -- | -- |
| 12:2 FTS | 149246-64-0 | | -- | | -- | | -- | 0 | | -- | | -- | | | -- | | 0 | 3.61 | | 3.61 | | -- | 1 | -- | -- | -- | 0 | -- | -- | -- | -- | -- | -- | -- |
| * Measurement for *Metaphire guillelmi* | | | | | | | | | | | | | | | | | | | | |  |  |  |  |  |  |  |  |  |  |  |  |  |  |
| ** Species codes: | |  | |  | |  | | |  | |  | |  |  | |  | | |  | |  |  |  |  |  |  |  |  |  |  |  |  |  |  |
| EF = *Eisenia fetida* | | | |  | |  | | |  | |  | |  |  | |  | | |  | |  |  |  |  |  |  |  |  |  |  |  |  |  |  |
| EA = *Eisenia andrei* | | | |  | |  | | |  | |  | |  |  | |  | | |  | |  |  |  |  |  |  |  |  |  |  |  |  |  |  |
| LT = *Lumbricus terrestris* | | | |  | |  | | |  | |  | |  |  | |  | | |  | |  |  |  |  |  |  |  |  |  |  |  |  |  |  |
| MX = *Lumbricus rebellus, Aporrectodea rosea, Dendrobaena octaedra, Dendrodrilus rubidus* | | | | | | | | | | | | | | | | | | | | |  |  |  |  |  |  |  |  |  |  |  |  |  |  |

| **Table S4. Comparison of mean whole body BSAFs (kg-OC/kg-ww) study qualities** | | | | | | | | | | | | | | | | | | | | | | | | | | | | | | | |
| --- | --- | --- | --- | --- | --- | --- | --- | --- | --- | --- | --- | --- | --- | --- | --- | --- | --- | --- | --- | --- | --- | --- | --- | --- | --- | --- | --- | --- | --- | --- | --- |
|  |  |  | **Low** | | | | | | | | **medium** | | | | | | | | | | **high** | | | | | | | **ANOVA** |  | **TurkeyHSD** |  |
| **Chemical** | **CAS #** | **Species *** | **average** | **median** | **standard deviation** | | **n** | **Minimum** | | **Maximum** | **average** | | **median** | **standard deviation** | | **n** | | **Minimum** | | **Maximum** | **average** | | **median** | **standard deviation** | **n** | **Minimum** | **Maximum** | **p-value** | **low-medium** | **low-high** | **medium-high** |
| **Carbonyl Compounds - OECD Structure Category 100** | | | | | | | | | | | | | | | | | | | | | | | | | | | | | | | |
| **Carboxylic acids - OECD Structure Category 102 - perfluoroalkyl carboxylic acids (PFCAs), their salts and esters** | | | | | | | | | | | | | | | | | | | | | | | | | | | | | | | |
| PFBA | 45048-62-2 | EF | 0.0244 | 0.0242 | 0.0186 | | 4 | 0.00198 | | 0.0474 | 0.00698 | | 0.00698 | 0.00263 | | 2 | | 0.00512 | | 0.00884 | 0.23 | | 0.23 | -- | 1 | 0.23 | 0.23 | 17% | 74% | 23% | 16% |
| PFPeA | 45167-47-3 | EF | 0.011 | 0.0027 | 0.0157 | | 7 | 0.00112 | | 0.0399 | 0.00465 | | 0.00465 | 0.000986 | | 2 | | 0.00395 | | 0.00535 | 0.101 | | 0.101 | -- | 1 | 0.101 | 0.101 | 16% | 100% | 14% | 22% |
| PFHxA | 92612-52-7 | MX, EF | 0.0056 | 0.00495 | 0.00361 | | 7 | 0.0006 | | 0.00997 | 0.109 | | 0.00465 | 0.186 | | 6 | | 0.000975 | | 0.459 | 0.0345 | | 0.0345 | 0.0395 | 2 | 0.00652 | 0.0624 | 43% | 53% | 54% | 95% |
| PFHpA | 120885-29-2 | EF | 0.00617 | 0.00498 | 0.00452 | | 8 | 0.000681 | | 0.0157 | 0.0018 | | 0.0018 | 0.0017 | | 2 | | 0.0006 | | 0.003 | 0.00915 | | 0.00915 | -- | 1 | 0.00915 | 0.00915 | 22% | 28% | 78% | 28% |
| PFOA | 45285-51-6 | MX, EF, LT, EA | 0.0139 | 0.00596 | 0.0176 | | 26 | 0.0000483 | | 0.0832 | 0.191 | | 0.0159 | 0.448 | | 7 | | 0.00105 | | 1.21 | 0.0172 | | 0.0075 | 0.0198 | 14 | 0.00318 | 0.0663 | 16% | 18% | 44% | 72% |
| PFNA | 72007-68-2 | MX, EF, LT | 0.0429 | 0.0364 | 0.0244 | | 15 | 0.01 | | 0.0969 | 0.141 | | 0.0232 | 0.275 | | 5 | | 0.0033 | | 0.632 | 0.0568 | | 0.0552 | 0.0386 | 8 | 0.016 | 0.14 | 59% | 78% | 84% | 56% |
| PFDA | 73829-36-4 | MX, EF, LT, EA | 0.168 | 0.0682 | 0.296 | | 19 | 0.0157 | | 1.09 | 0.463 | | 0.0349 | 0.936 | | 6 | | 0.00585 | | 2.36 | 0.143 | | 0.0637 | 0.191 | 7 | 0.0169 | 0.542 | 94% | 94% | 100% | 97% |
| PFUnDA | 196859-54-8 | MX, EF, LT, EA | 0.36 | 0.163 | 0.635 | | 19 | 0.0211 | | 2.16 | 0.732 | | 0.0581 | 1.2 | | 5 | | 0.0126 | | 2.81 | 0.235 | | 0.102 | 0.349 | 7 | 0.0377 | 1.01 | 90% | 96% | 91% | 100% |
| PFDoDA | 171978-95-3 | MX, EF, LT, EA | 0.635 | 0.4 | 0.751 | | 18 | 0.0193 | | 2.68 | 0.734 | | 0.0666 | 1.37 | | 4 | | 0.0196 | | 2.78 | 0.551 | | 0.256 | 0.776 | 7 | 0.13 | 2.28 | 31% | 28% | 98% | 45% |
| PFTrDA | 862374-87-6 | EF | -- | -- | -- | | 0 | -- | | -- | 0.214 | | 0.214 | -- | | 1 | | 0.214 | | 0.214 | 5.87 | | 5.87 | -- | 1 | 5.87 | 5.87 | -- | -- | -- | -- |
| PFTeDA | 365971-87-5 | EF | -- | -- | -- | | 0 | -- | | -- | 0.121 | | 0.121 | -- | | 1 | | 0.121 | | 0.121 | 4.7 | | 4.7 | -- | 1 | 4.7 | 4.7 | -- | -- | -- | -- |
| PFPeDA | 1002-84-2 | -- | -- | -- | -- | | 0 | -- | | -- | -- | | -- | -- | | 0 | | -- | | -- | -- | | -- | -- | 0 | -- | -- | -- | -- | -- | -- |
| PFHxDA | 67905-19-5 | EF | -- | -- | -- | | 0 | -- | | -- | 0.0814 | | 0.0814 | -- | | 1 | | 0.0814 | | 0.0814 | 2.09 | | 2.09 | -- | 1 | 2.09 | 2.09 | -- | -- | -- | -- |
| PFOcDA | 16517-11-6 | -- | -- | -- | -- | | 0 | -- | | -- | -- | | -- | -- | | 0 | | -- | | -- | -- | | -- | -- | 0 | -- | -- | -- | -- | -- | -- |
| **Carboxylic acids - OECD Structure Category 103.01 - perfluoroalkyl carbonyl amides / amido ethanols and other alcohols** | | | | | | | | | | | | | | | | | | | | | | | | | | | | | | | |
| PFOAB | 90179-39-8 | LT | -- | -- | -- | | 0 | -- | | -- | 0.0813 | | 0.0689 | 0.0361 | | 3 | | 0.053 | | 0.122 | -- | | -- | -- | 0 | -- | -- | -- | -- | -- | -- |
| PFOAAmS | 45305-66-6 | LT | -- | -- | -- | | 0 | -- | | -- | 0.0318 | | 0.0265 | 0.014 | | 3 | | 0.0212 | | 0.0477 | -- | | -- | -- | 0 | -- | -- | -- | -- | -- | -- |
| **Sulfonyl Compounds - OECD Structure Category 200** | | | | | | | | | | | | | | | | | | | | | | | | | | | | | | | |
| **Sulfonic Acids - OECD Structure Category 202 - perfluoroalkane sulfonic acids (PFSAs), their salts and esters** | | | | | | | | | | | | | | | | | | | | | | | | | | | | | | | |
| PFBS | 375-73-5 | MX, EF, EA | 0.0934 | 0.0459 | 0.124 | | 18 | 0.00273 | | 0.513 | 0.24 | | 0.057 | 0.335 | | 6 | | 0.0006 | | 0.775 | 0.00668 | | 0.00668 | 0.00435 | 2 | 0.0036 | 0.00975 | 32% | 91% | 29% | 48% |
| PFPeS | 2706-91-4 | EF | -- | -- | -- | | 0 | -- | | -- | 0.0721 | | 0.0721 | -- | | 1 | | 0.0721 | | 0.0721 | -- | | -- | -- | 0 | -- | -- | -- | -- | -- | -- |
| PFHxS | 355-46-4 | MX, EF, EA | 0.575 | 0.12 | 1.86 | | 18 | 0.00663 | | 8.02 | 0.312 | | 0.1 | 0.438 | | 6 | | 0.00292 | | 1.09 | 0.215 | | 0.221 | 0.167 | 6 | 0.0355 | 0.481 | 71% | 85% | 89% | 69% |
| PFHpS | 375-92-8 | EF | 0.366 | 0.366 | -- | | 1 | 0.366 | | 0.366 | 0.0546 | | 0.0546 | 0.00164 | | 2 | | 0.0535 | | 0.0558 | 0.425 | | 0.425 | -- | 1 | 0.425 | 0.425 | 2% | -- | -- | -- |
| Cl-PFOS | 1651215-26-7 | EF | -- | -- | -- | | 0 | -- | | -- | 0.0651 | | 0.0651 | -- | | 1 | | 0.0651 | | 0.0651 | 1.31 | | 1.31 | -- | 1 | 1.31 | 1.31 | -- | -- | -- | -- |
| PFOS | 1763-23-1 | MX, EF, EA, LT | 0.154 | 0.0732 | 0.312 | | 38 | 0.00073 | | 1.92 | 0.208 | | 0.0589 | 0.312 | | 9 | | 0.00728 | | 0.861 | 0.176 | | 0.0168 | 0.328 | 13 | 0.00968 | 1.21 | 86% | 97% | 90% | 86% |
| br-PFOS | -- | -- | -- | -- | -- | | 0 | -- | | -- | -- | | -- | -- | | 0 | | -- | | -- | -- | | -- | -- | 0 | -- | -- | -- | -- | -- | -- |
| PFNS | 68259-12-1 | EF | -- | -- | -- | | 0 | -- | | -- | 0.0698 | | 0.0698 | -- | | 1 | | 0.0698 | | 0.0698 | 1.03 | | 1.03 | -- | 1 | 1.03 | 1.03 | -- | -- | -- | -- |
| PFDS | 335-77-3 | MX, EF | 0.609 | 0.579 | 0.439 | | 3 | 0.186 | | 1.06 | 1.19 | | 0.546 | 1.51 | | 3 | | 0.102 | | 2.91 | 0.428 | | 0.142 | 0.66 | 4 | 0.0221 | 1.41 | 47% | 100% | 57% | 52% |
| PFDoDS | 79780-39-5 | EF | -- | -- | -- | | 0 | -- | | -- | 0.223 | | 0.223 | -- | | 1 | | 0.223 | | 0.223 | -- | | -- | -- | 0 | -- | -- | -- | -- | -- | -- |
| **Sulfonic Acids - OECD Structure Category 203.01 - perfluoroalkane sulfonyl amides/amido ethanols (xFASA/Es) and other alcohols** | | | | | | | | | | | | | | | | | | | | | | | | | | | | | | | |
| PFOSB | 75046-16-1 | LT | -- | -- | -- | | 0 | -- | | -- | 0.0477 | | 0.0424 | 0.0191 | | 3 | | 0.0318 | | 0.0689 | -- | | -- | -- | 0 | -- | -- | -- | -- | -- | -- |
| PFOSAmS | 70225-25-1 | LT | -- | -- | -- | | 0 | -- | | -- | 0.0265 | | 0.0212 | 0.014 | | 3 | | 0.0159 | | 0.0424 | -- | | -- | -- | 0 | -- | -- | -- | -- | -- | -- |
| PFOSA | 754-91-6 | EF | 0.184 | 0.0164 | 0.346 | | 4 | 0.00224 | | 0.703 | -- | | -- | -- | | 0 | | -- | | -- | -- | | -- | -- | 0 | -- | -- | -- | -- | -- | -- |
| MeFOSA | 31506-32-8 | EF | 0.0896 | 0.0896 | -- | | 1 | 0.0896 | | 0.0896 | -- | | -- | -- | | 0 | | -- | | -- | -- | | -- | -- | 0 | -- | -- | -- | -- | -- | -- |
| 6:2 FTAB | 34455-29-3 | EF | -- | -- | -- | | 0 | -- | | -- | 0.00922 | | 0.0093 | 0.00337 | | 3 | | 0.00581 | | 0.0126 | 0.23 | | 0.23 | -- | 1 | 0.23 | 0.23 | 2% | -- | -- | 2% |
| 8:2 FTAB | 34455-21-5 | EF | -- | -- | -- | | 0 | -- | | -- | 0.0181 | | 0.0181 | 0.0112 | | 2 | | 0.0102 | | 0.026 | 0.0312 | | 0.0312 | -- | 1 | 0.0312 | 0.0312 | 57% | -- | -- | -- |
| 10:2 FTAB | 34455-35-1 | EF | -- | -- | -- | | 0 | -- | | -- | 0.03 | | 0.03 | -- | | 1 | | 0.03 | | 0.03 | -- | | -- | -- | 0 | -- | -- | -- | -- | -- | -- |
| 12:2 FTAB | 278598-45-1 | EF | -- | -- | -- | | 0 | -- | | -- | 0.026 | | 0.026 | -- | | 1 | | 0.026 | | 0.026 | -- | | -- | -- | 0 | -- | -- | -- | -- | -- | -- |
| N-EtFose | 1691-99-2 | EF | -- | -- | -- | | 0 | -- | | -- | -- | | -- | -- | | 0 | | -- | | -- | 0.0638 | | 0.0638 | 0.0347 | 2 | 0.0392 | 0.0884 | -- | -- | -- | -- |
| 7:3 FtB | 171184-15-9 | EF | -- | -- | -- | | 0 | -- | | -- | 0.0036 | | 0.0036 | 0.000822 | | 2 | | 0.00302 | | 0.00419 | 0.0897 | | 0.0897 | -- | 1 | 0.0897 | 0.0897 | 6% | -- | -- | -- |
| 9:3 FtB | 171184-16-0 | EF | -- | -- | -- | | 0 | -- | | -- | 0.01 | | 0.01 | 0.00559 | | 2 | | 0.00604 | | 0.014 | 0.0702 | | 0.0702 | -- | 1 | 0.0702 | 0.0702 | 22% | -- | -- | -- |
| 11:3 FtB | 171184-17-1 | EF | -- | -- | -- | | 0 | -- | | -- | 0.0153 | | 0.0153 | 0.0112 | | 2 | | 0.00744 | | 0.0232 | 0.105 | | 0.105 | -- | 1 | 0.105 | 0.105 | 28% | -- | -- | -- |
| 13:3 FtB | 1513864-13-5 | EF | -- | -- | -- | | 0 | -- | | -- | 0.0086 | | 0.0086 | 0.00493 | | 2 | | 0.00512 | | 0.0121 | 0.207 | | 0.207 | -- | 1 | 0.207 | 0.207 | 14% | -- | -- | -- |
| 5:1:2 FtB | 171184-02-4 | EF | -- | -- | -- | | 0 | -- | | -- | 0.00267 | | 0.00267 | 0.000164 | | 2 | | 0.00256 | | 0.00279 | 0.023 | | 0.023 | -- | 1 | 0.023 | 0.023 | 2% | -- | -- | -- |
| 7:1:2 FtB | 171184-03-5 | EF | -- | -- | -- | | 0 | -- | | -- | 0.0036 | | 0.0036 | 0.000822 | | 2 | | 0.00302 | | 0.00419 | 0.0819 | | 0.0819 | -- | 1 | 0.0819 | 0.0819 | 6% | -- | -- | -- |
| 9:1:2 FtB | 171184-04-6 | EF | -- | -- | -- | | 0 | -- | | -- | 0.00965 | | 0.00965 | 0.00477 | | 2 | | 0.00628 | | 0.013 | 0.0624 | | 0.0624 | -- | 1 | 0.0624 | 0.0624 | 20% | -- | -- | -- |
| 11:1:2 FtB | 171184-05-7 | EF | -- | -- | -- | | 0 | -- | | -- | 0.0148 | | 0.0148 | 0.0107 | | 2 | | 0.00721 | | 0.0223 | 0.0936 | | 0.0936 | -- | 1 | 0.0936 | 0.0936 | 29% | -- | -- | -- |
| 13:1:2 FtB | 1513864-14-6 | EF | -- | -- | -- | | 0 | -- | | -- | 0.00872 | | 0.00872 | 0.00477 | | 2 | | 0.00535 | | 0.0121 | 0.156 | | 0.156 | -- | 1 | 0.156 | 0.156 | 15% | -- | -- | -- |
| **Fluorotelomer related compounds - OECD Structure Category 400** | | | | | | | | | | | | | | | | | | | | | | | | | | | | | | | |
| **Fluorotelomer - OECD Structure Category: 402.04 - n:2 fluorotelomer olefins (n:2 FTOs)** | | | | | | | | | | | | | | | | | | | | | | | | | | | | | | | |
| 10:2 FTOH | 865-86-1 | EF | 0.0404 | 0.0404 | -- | | 1 | 0.0404 | | 0.0404 | -- | | -- | -- | | 0 | | -- | | -- | -- | | -- | -- | 0 | -- | -- | -- | -- | -- | -- |
| **Fluorotelomer - OECD Structure Category: 402.04 - n:2 fluorotelomer alcohol, phosphate esters (PAPs)** | | | | | | | | | | | | | | | | | | | | | | | | | | | | | | | |
| 6:2 diPAP | 57677-95-9 | MG | -- | -- | -- | | 0 | -- | | -- | -- | | -- | -- | | 0 | | -- | | -- | 0.0384 | | 0.0384 | -- | 1 | 0.0384 | 0.0384 | -- | -- | -- | -- |
| 8:2 diPAP | 678-41-1 | -- | -- | -- | -- | | 0 | -- | | -- | -- | | -- | -- | | 0 | | -- | | -- | -- | | -- | -- | 0 | -- | -- | -- | -- | -- | -- |
| **Fluorotelomer - OECD Structure Category: 402.07 - Fluorotelomer sulfonate** | | | | | | | | | | | | | | | | | | | | | | | | | | | | | | | |
| 4:2 FTS | 757124-72-4 | EF | 0.00175 | 0.00175 | -- | | 1 | 0.00175 | | 0.00175 | -- | | -- | -- | | 0 | | -- | | -- | -- | | -- | -- | 0 | -- | -- | -- | -- | -- | -- |
| 6:2 FTS | 27619-97-2 | MX, EF | 0.314 | 0.239 | 0.337 | | 6 | 0.0179 | | 0.816 | 3.35 | | 3.35 | 2.05 | | 2 | | 1.9 | | 4.8 | 11 | | 11 | 15.1 | 2 | 0.342 | 21.7 | 9% | 16% | 17% | 100% |
| 8:2 FTS | 39108-34-4 | EF | 0.448 | 0.448 | -- | | 1 | 0.448 | | 0.448 | -- | | -- | -- | | 0 | | -- | | -- | 23.9 | | 23.9 | -- | 1 | 23.9 | 23.9 | -- | -- | -- | -- |
| 10:2 FTS | 120226-60-0 | EF | -- | -- | -- | | 0 | -- | | -- | -- | | -- | -- | | 0 | | -- | | -- | 5.11 | | 5.11 | -- | 1 | 5.11 | 5.11 | -- | -- | -- | -- |
| 12:2 FTS | 149246-64-0 | EF | -- | -- | -- | | 0 | -- | | -- | -- | | -- | -- | | 0 | | -- | | -- | 3.61 | | 3.61 | -- | 1 | 3.61 | 3.61 | -- | -- | -- | -- |
| * Species codes: | | | | | |  | | |  | | |  | | |  | |  | |  | | |  |  |  |  |  |  |  |  |  |  |
| EF = *Eisenia fetida* | | | | | | | | |  | | |  | | |  | |  | |  | | |  |  |  |  |  |  |  |  |  |  |
| EA = *Eisenia andrei* | | | | | | | | |  | | |  | | |  | |  | |  | | |  |  |  |  |  |  |  |  |  |  |
| LT = *Lumbricus terrestris* | | | | | | | | |  | | |  | | |  | |  | |  | | |  |  |  |  |  |  |  |  |  |  |
| MG = *Metaphire guillelmi* | | | | | |  | | |  | | |  | | |  | |  | |  | | |  |  |  |  |  |  |  |  |  |  |
| MX = *Lumbricus rebellus, Aporrectodea rosea, Dendrobaena octaedra, Dendrodrilus rubidus* | | | | | | | | | | | | | | | | | | | | | | |  |  |  |  |  |  |  |  |  |

| **Table S5.** Comparison of mean BSAFs (kg-OC/kg-ww) measured in the laboratory and field settings. | | | | | | | | | | | | | | | | | | | | | | | | | | |
| --- | --- | --- | --- | --- | --- | --- | --- | --- | --- | --- | --- | --- | --- | --- | --- | --- | --- | --- | --- | --- | --- | --- | --- | --- | --- | --- |
|  |  |  | **Laboratory** | | | | | | | | | **Field** | | | | | | | | | | | | **Comparison of Means *** | | |
| **Chemical** | **CAS #** | **Species *** | **average** | **median** | **standard deviation** | **n** | **Percentile 10th** | **Percentile 90th** | **Minimum** | **Maximum** | **average** | | **median** | **standard deviation** | **n** | **Percentile 10th** | **Percentile 90th** | | **Minimum** | | **Maximum** | | **p-value** | | **95% Confidence Limits** | **95% Confidence Limits** |
| **Carbonyl Compounds - OECD Structure Category 100** | | | | | | | | | | | | | | | | | | | | | | | | | | |
| **Carboxylic Acids - OECD Structure 102- perfluoroalkyl carboxylic acids (PFCAs), their salts and esters** | | | | | | | | | | | | | | | | | | | | | | | | | | |
| PFBA | 45048-62-2 | EF | 0.0186 | 0.0159 | 0.017 | 6 | 0.00355 | 0.0365 | 0.00198 | 0.0474 | | 0.23 | 0.23 | -- | 1 | 0.23 | 0.23 | | 0.23 | | 0.23 | | 0.00158 | | -5.69 | -3.22 |
| PFPeA | 45167-47-3 | EF | 0.00958 | 0.00393 | 0.0138 | 9 | 0.0013 | 0.0291 | 0.00112 | 0.0399 | | 0.101 | 0.101 | -- | 1 | 0.101 | 0.101 | | 0.101 | | 0.101 | | 0.0000704 | | -6.41 | -4.47 |
| PFHxA | 92612-52-7 | MX, EF | 0.00487 | 0.00465 | 0.0031 | 12 | 0.00112 | 0.00957 | 0.0006 | 0.00997 | | 0.234 | 0.182 | 0.204 | 3 | 0.0863 | 0.404 | | 0.0624 | | 0.459 | | 0.0106 | | -5.92 | -1.76 |
| PFHpA | 120885-29-2 | EF | 0.00565 | 0.00495 | 0.00436 | 11 | 0.000681 | 0.00915 | 0.0006 | 0.0157 | | -- | -- | -- | 0 | -- | -- | | -- | | -- | | -- | | -- | -- |
| PFOA | 45285-51-6 | MX, EF, LT, EA | 0.0134 | 0.00517 | 0.016 | 43 | 0.0013 | 0.0259 | 0.0000483 | 0.0832 | | 0.341 | 0.0721 | 0.577 | 4 | 0.0292 | 0.868 | | 0.0133 | | 1.21 | | 0.0532 | | -5.66 | 0.0658 |
| PFNA | 72007-68-2 | MX, EF, LT | 0.0403 | 0.0364 | 0.0238 | 25 | 0.011 | 0.069 | 0.0033 | 0.0969 | | 0.265 | 0.14 | 0.323 | 3 | 0.0461 | 0.533 | | 0.0225 | | 0.632 | | 0.289 | | -5.37 | 2.62 |
| PFDA | 73829-36-4 | MX, EF, LT, EA | 0.132 | 0.0635 | 0.25 | 28 | 0.0165 | 0.189 | 0.00585 | 1.09 | | 0.815 | 0.44 | 1.05 | 4 | 0.12 | 1.81 | | 0.027 | | 2.36 | | 0.158 | | -4.6 | 1.14 |
| PFUnDA | 196859-54-8 | MX, EF, LT, EA | 0.278 | 0.129 | 0.545 | 27 | 0.0274 | 0.371 | 0.0126 | 2.16 | | 1.16 | 0.884 | 1.17 | 4 | 0.275 | 2.27 | | 0.07 | | 2.81 | | 0.119 | | -4.05 | 0.715 |
| PFDoDA | 171978-95-3 | MX, EF, LT, EA | 0.499 | 0.288 | 0.66 | 26 | 0.0666 | 0.949 | 0.0193 | 2.68 | | 1.76 | 2.28 | 1.37 | 3 | 0.614 | 2.68 | | 0.197 | | 2.78 | | 0.232 | | -4.75 | 1.92 |
| PFTrDA | 862374-87-6 | EF | 0.214 | 0.214 | -- | 1 | 0.214 | 0.214 | 0.214 | 0.214 | | 5.87 | 5.87 | -- | 1 | 5.87 | 5.87 | | 5.87 | | 5.87 | | -- | | -- | -- |
| PFTeDA | 365971-87-5 | EF | 0.121 | 0.121 | -- | 1 | 0.121 | 0.121 | 0.121 | 0.121 | | 4.7 | 4.7 | -- | 1 | 4.7 | 4.7 | | 4.7 | | 4.7 | | -- | | -- | -- |
| PFPeDA | 1002-84-2 | -- | -- | -- | -- | 0 | -- | -- | -- | -- | | -- | -- | -- | 0 | -- | -- | | -- | | -- | | -- | | -- | -- |
| PFHxDA | 67905-19-5 | EF | 0.0814 | 0.0814 | -- | 1 | 0.0814 | 0.0814 | 0.0814 | 0.0814 | | 2.09 | 2.09 | -- | 1 | 2.09 | 2.09 | | 2.09 | | 2.09 | | -- | | -- | -- |
| PFOcDA | 16517-11-6 | -- | -- | -- | -- | 0 | -- | -- | -- | -- | | -- | -- | -- | 0 | -- | -- | | -- | | -- | | -- | | -- | -- |
| **Sulfonyl Compounds - OECD Structure Category 200** | | | | | | | | | | | | | | | | | | | | | | | | | | |
| **Sulfonic Acids - OECD Structure 202** | | |  |  |  |  |  |  |  |  | |  |  |  |  |  |  | |  | |  | |  | |  |  |
| PFBS | 375-73-5 | MX, EF, EA | 0.0783 | 0.0443 | 0.113 | 23 | 0.0029 | 0.199 | 0.0006 | 0.513 | | 0.444 | 0.545 | 0.393 | 3 | 0.117 | 0.729 | | 0.00975 | | 0.775 | | 0.355 | | -7.27 | 3.93 |
| PFPeS | 2706-91-4 | EF | 0.0721 | 0.0721 | -- | 1 | 0.0721 | 0.0721 | 0.0721 | 0.0721 | | -- | -- | -- | 0 | -- | -- | | -- | | -- | | -- | | -- | -- |
| PFHxS | 355-46-4 | MX, EF, EA | 0.437 | 0.124 | 1.52 | 27 | 0.0118 | 0.396 | 0.00292 | 8.02 | | 0.571 | 0.574 | 0.522 | 3 | 0.152 | 0.987 | | 0.0468 | | 1.09 | | 0.325 | | -4.91 | 2.43 |
| PFHpS | 375-92-8 | EF | 0.159 | 0.0558 | 0.18 | 3 | 0.0539 | 0.304 | 0.0535 | 0.366 | | 0.425 | 0.425 | -- | 1 | 0.425 | 0.425 | | 0.425 | | 0.425 | | 0.155 | | -5 | 0.458 |
| Cl-PFOS | 1651215-26-7 | EF | 0.0651 | 0.0651 | -- | 1 | 0.0651 | 0.0651 | 0.0651 | 0.0651 | | 1.31 | 1.31 | -- | 1 | 1.31 | 1.31 | | 1.31 | | 1.31 | | -- | | -- | -- |
| PFOS | 1763-23-1 | MX, EF, EA, LT | 0.128 | 0.0576 | 0.262 | 57 | 0.011 | 0.272 | 0.00073 | 1.92 | | 0.898 | 0.861 | 0.295 | 3 | 0.671 | 1.14 | | 0.623 | | 1.21 | | 0.00000854 | | -3.44 | -2.18 |
| br-PFOS | -- | -- | -- | -- | -- | 0 | -- | -- | -- | -- | | -- | -- | -- | 0 | -- | -- | | -- | | -- | | -- | | -- | -- |
| PFNS | 68259-12-1 | EF | 0.0698 | 0.0698 | -- | 1 | 0.0698 | 0.0698 | 0.0698 | 0.0698 | | 1.03 | 1.03 | -- | 1 | 1.03 | 1.03 | | 1.03 | | 1.03 | | -- | | -- | -- |
| PFDS | 335-77-3 | MX, EF | 0.319 | 0.186 | 0.377 | 7 | 0.0385 | 0.772 | 0.0221 | 1.06 | | 1.62 | 1.41 | 1.2 | 3 | 0.718 | 2.61 | | 0.546 | | 2.91 | | 0.0237 | | -3.79 | -0.384 |
| PFDoDS | 79780-39-5 | EF | 0.223 | 0.223 | -- | 1 | 0.223 | 0.223 | 0.223 | 0.223 | | -- | -- | -- | 0 | -- | -- | | -- | | -- | | -- | | -- | -- |
| **Sulfonic Acids - OECD Structure Category 203.01 - perfluoroalkane sulfonyl amides/amido ethanols (xFASA/Es) and other alcohols** | | | | | | | | | | | | | | | | | | | | | | | | | | |
| PFOSB | 75046-16-1 | LT | 0.0477 | 0.0424 | 0.0191 | 3 | 0.0339 | 0.0636 | 0.0318 | 0.0689 | | -- | -- | -- | 0 | -- | -- | | -- | | -- | | -- | | -- | -- |
| PFOSAmS | 70225-25-1 | LT | 0.0265 | 0.0212 | 0.014 | 3 | 0.017 | 0.0382 | 0.0159 | 0.0424 | | -- | -- | -- | 0 | -- | -- | | -- | | -- | | -- | | -- | -- |
| PFOSA | 754-91-6 | EF | 0.184 | 0.0164 | 0.346 | 4 | 0.00333 | 0.5 | 0.00224 | 0.703 | | -- | -- | -- | 0 | -- | -- | | -- | | -- | | -- | | -- | -- |
| MeFOSA | 31506-32-8 | EF | 0.0896 | 0.0896 | -- | 1 | 0.0896 | 0.0896 | 0.0896 | 0.0896 | | -- | -- | -- | 0 | -- | -- | | -- | | -- | | -- | | -- | -- |
| 6:2 FTAB | 34455-29-3 | EF | 0.00922 | 0.0093 | 0.00337 | 3 | 0.00651 | 0.0119 | 0.00581 | 0.0126 | | 0.23 | 0.23 | -- | 1 | 0.23 | 0.23 | | 0.23 | | 0.23 | | 0.00468 | | -5.7 | -3.77 |
| 8:2 FTAB | 34455-21-5 | EF | 0.0181 | 0.0181 | 0.0112 | 2 | 0.0118 | 0.0245 | 0.0102 | 0.026 | | 0.0312 | 0.0312 | -- | 1 | 0.0312 | 0.0312 | | 0.0312 | | 0.0312 | | 0.398 | | -10 | 1.82 |
| 10:2 FTAB | 34455-35-1 | EF | 0.03 | 0.03 | -- | 1 | 0.03 | 0.03 | 0.03 | 0.03 | | -- | -- | -- | 0 | -- | -- | | -- | | -- | | -- | | -- | -- |
| 12:2 FTAB | 278598-45-1 | EF | 0.026 | 0.026 | -- | 1 | 0.026 | 0.026 | 0.026 | 0.026 | | -- | -- | -- | 0 | -- | -- | | -- | | -- | | -- | | -- | -- |
| N-EtFose | 1691-99-2 | EF | 0.0638 | 0.0638 | 0.0347 | 2 | 0.0441 | 0.0834 | 0.0392 | 0.0884 | | -- | -- | -- | 0 | -- | -- | | -- | | -- | | -- | | -- | -- |
| 7:3 FtB | 171184-15-9 | EF | 0.00302 | 0.00302 | -- | 1 | 0.00302 | 0.00302 | 0.00302 | 0.00302 | | 0.0469 | 0.0469 | 0.0605 | 2 | 0.0127 | 0.0812 | | 0.00419 | | 0.0897 | | 0.439 | | -23.4 | 15.5 |
| 9:3 FtB | 171184-16-0 | EF | 0.01 | 0.01 | 0.00559 | 2 | 0.00684 | 0.0132 | 0.00604 | 0.014 | | 0.0702 | 0.0702 | -- | 1 | 0.0702 | 0.0702 | | 0.0702 | | 0.0702 | | 0.129 | | -10 | 0.622 |
| 11:3 FtB | 171184-17-1 | EF | 0.0153 | 0.0153 | 0.0112 | 2 | 0.00902 | 0.0217 | 0.00744 | 0.0232 | | 0.105 | 0.105 | -- | 1 | 0.105 | 0.105 | | 0.105 | | 0.105 | | 0.17 | | -11.6 | 2.91 |
| 13:3 FtB | 1513864-13-5 | EF | 0.0086 | 0.0086 | 0.00493 | 2 | 0.00581 | 0.0114 | 0.00512 | 0.0121 | | 0.207 | 0.207 | -- | 1 | 0.207 | 0.207 | | 0.207 | | 0.207 | | 0.0833 | | -10.3 | 0.62 |
| 5:1:2 FtB | 171184-02-4 | EF | 0.00267 | 0.00267 | 0.000164 | 2 | 0.00258 | 0.00277 | 0.00256 | 0.00279 | | 0.023 | 0.023 | -- | 1 | 0.023 | 0.023 | | 0.023 | | 0.023 | | 0.0129 | | -6.48 | -5.37 |
| 7:1:2 FtB | 171184-03-5 | EF | 0.0036 | 0.0036 | 0.000822 | 2 | 0.00314 | 0.00407 | 0.00302 | 0.00419 | | 0.0819 | 0.0819 | -- | 1 | 0.0819 | 0.0819 | | 0.0819 | | 0.0819 | | 0.033 | | -7.71 | -3.57 |
| 9:1:2 FtB | 171184-04-6 | EF | 0.00965 | 0.00965 | 0.00477 | 2 | 0.00695 | 0.0124 | 0.00628 | 0.013 | | 0.0624 | 0.0624 | -- | 1 | 0.0624 | 0.0624 | | 0.0624 | | 0.0624 | | 0.119 | | -9.34 | -0.0713 |
| 11:1:2 FtB | 171184-05-7 | EF | 0.0148 | 0.0148 | 0.0107 | 2 | 0.00872 | 0.0208 | 0.00721 | 0.0223 | | 0.0936 | 0.0936 | -- | 1 | 0.0936 | 0.0936 | | 0.0936 | | 0.0936 | | 0.175 | | -11.6 | 2.81 |
| 13:1:2 FtB | 1513864-14-6 | EF | 0.00872 | 0.00872 | 0.00477 | 2 | 0.00602 | 0.0114 | 0.00535 | 0.0121 | | 0.156 | 0.156 | -- | 1 | 0.156 | 0.156 | | 0.156 | | 0.156 | | 0.087 | | -10 | 0.359 |
| **Fluorotelomer related compounds - OECD Structure Category 400** | | | | | | | | | | | | | | | | | | | | | | | | | | |
| **Fluorotelomer - OECD Structure Category: 402.04 - n:2 fluorotelomer olefins (n:2 FTOs)** | | | | | | | | | | | | | | | | | | | | | | | | | | |
| 10:2 Fluorotelomer alcohol | 10:2 FTOH | 865-86-1 | EF | 0.0404 | 0.0404 | -- | 1 | 0.0404 | 0.0404 | 0.0404 | | 0.0404 | -- | -- | -- | 0 | -- | | -- | | -- | | -- | | -- | -- |
| **Fluorotelomer - OECD Structure Category: 402.04 - n:2 fluorotelomer alcohol, phosphate esters (PAPs)** | | | | | | | | | | | | | | | | | | | | | | | | | | |
| 6:2 diPAP | 57677-95-9 | MG | 0.0384 | 0.0384 | -- | 1 | 0.0384 | 0.0384 | 0.0384 | 0.0384 | | -- | -- | -- | 0 | -- | -- | | -- | | -- | | -- | | -- | -- |
| 8:2 diPAP | 678-41-1 | -- | -- | -- | -- | 0 | -- | -- | -- | -- | | -- | -- | -- | 0 | -- | -- | | -- | | -- | | -- | | -- | -- |
| **Fluorotelomer - OECD Structure Category: 402.07 - Fluorotelomer sulfonate** | | | | | | | | | | | | | | | | | | | | | | | | | | |
| 4:2 FTS | 757124-72-4 | EF | 0.00175 | 0.00175 | -- | 1 | 0.00175 | 0.00175 | 0.00175 | 0.00175 | | -- | -- | -- | 0 | -- | -- | | -- | | -- | | -- | | -- | -- |
| 6:2 FTS | 27619-97-2 | MX, EF | 0.318 | 0.342 | 0.308 | 7 | 0.0222 | 0.654 | 0.0179 | 0.816 | | 9.47 | 4.8 | 10.7 | 3 | 2.48 | 18.3 | | 1.9 | | 21.7 | | 0.0101 | | -6.11 | -1.32 |
| 8:2 FTS | 39108-34-4 | EF | 0.448 | 0.448 | -- | 1 | 0.448 | 0.448 | 0.448 | 0.448 | | 23.9 | 23.9 | -- | 1 | 23.9 | 23.9 | | 23.9 | | 23.9 | | -- | | -- | -- |
| 10:2 FTS | 120226-60-0 | EF | -- | -- | -- | 0 | -- | -- | -- | -- | | 5.11 | 5.11 | -- | 1 | 5.11 | 5.11 | | 5.11 | | 5.11 | | -- | | -- | -- |
| 12:2 FTS | 149246-64-0 | EF | -- | -- | -- | 0 | -- | -- | -- | -- | | 3.61 | 3.61 | -- | 1 | 3.61 | 3.61 | | 3.61 | | 3.61 | | -- | | -- | -- |
| * Comparison of means for pairs with two or more measurements for laboratory and field were done with anova algorithm and comparison of means for pairs with one measurement in the laboratory or field were done with One Sample t-test (R Core Team 2013). | | | | | | | | | | | | | | | | | | | | | | | | | | |
|  |  |  |  |  |  |  |  |  |  |  | |  |  |  |  |  | |  | |  | |  |  | |  |  |
| **Species codes:** |  |  |  |  |  |  |  |  |  |  | |  |  |  |  |  | |  | |  | |  |  | |  |  |
| EF = Eisenia fetida | |  |  |  |  |  |  |  |  |  | |  |  |  |  |  | |  | |  | |  |  | |  |  |
| EA = Eisenia andrei | |  |  |  |  |  |  |  |  |  | |  |  |  |  |  | |  | |  | |  |  | |  |  |
| LT = Lumbricus terrestris | |  |  |  |  |  |  |  |  |  | |  |  |  |  |  | |  | |  | |  |  | |  |  |
| MG = *Metaphire guillelmi* | |  |  |  |  |  |  |  |  |  | |  |  |  |  |  | |  | |  | |  |  | |  |  |
| MX = Lumbricus rebellus, Aporrectodea rosea, Dendrobaena octaedra, Dendrodrilus rubidus, | | | | | | | | | | | | | | | | | | | | | | | | | | |

| **Table S6. Uptake and elimination rates for PFAS by oligochaetes** | | | | | | | | | | |
| --- | --- | --- | --- | --- | --- | --- | --- | --- | --- | --- |
| **Terrestrial oligochaetes**: *Eisenia fetida, Metaphire guillelmi, and Lumbricus terrestris* | | | | | | | | | | |
|  | ks | | ke | t1/2 | OC | silt | clay | sand | BSAF | reference (species) |
| Chemical | kg-OC/kg-ww/d | | 1/d | days | % | kg/kg | kg/kg | kg/kg | kg-OC/kg-ww |  |
| **Carbonyl Compounds - OECD Structure Category 100** | | | | | |  |  |  |  |  |
| **Carboxylic Acids - OECD Structure 102** | | | | |  |  |  |  |  |  |
| PFHxA | 0.00120 | | 0.187 | 3.70 | 4.88 | 64.0 | 24.0 | 12.0 | 0.006525 | 3 EF |
| PFHpA | 0.001155 | | 0.1260 | 5.5 | 4.88 | 64.0 | 24.0 | 12.0 | 0.00915 | 3 EF |
| PFOA | 0.001118 | | 0.1140 | 6.1 | 4.88 | 64.0 | 24.0 | 12.0 | 0.009825 | 3 EF |
|  | 0.000788 | | 0.2160 | 3.21 | 0.690 | 57.2 | 9.13 | 32.7 | 0.00366 | 5 EF |
|  | 0.000711 | | 0.224 | 3.09 | 0.715 | 80.7 | 12.5 | 6.80 | 0.00318 | 5 EF |
|  | 0.001076 | | 0.229 | 3.03 | 1.15 | 69.0 | 8.63 | 22.3 | 0.00469 | 5 EF |
|  | 0.001165 | | 0.26 | 2.67 | 1.26 | 83.1 | 14.5 | 2.36 | 0.00449 | 5 EF |
|  | 0.00225 | | 0.485 | 1.43 | 1.38 | 85.1 | 12.1 | 2.83 | 0.00461 | 5 EF |
|  | 0.00205 | | 0.397 | 1.75 | 1.66 | 79.4 | 9.93 | 10.7 | 0.00517 | 5 EF |
|  | 0.001422 | | 0.363 | 1.91 | 1.95 | 78.8 | 9.40 | 11.9 | 0.00392 | 5 EF |
|  | 0.0321 | | 1.22880 | 0.56 | 6.00 | -- | -- | -- | 0.0260 | 4 EF |
| PFNA | 0.001575 | | 0.0990 | 7.0 | 4.88 | 64.0 | 24.0 | 12.0 | 0.015975 | 3 EF |
|  | 0.031824 | | 0.6432 | 1.08 | 6.00 | -- | -- | -- | 0.0494 | 4 EF |
| PFDA | 0.003375 | | 0.0830 | 8.4 | 4.88 | 64.0 | 24.0 | 12.0 | 0.041 | 3 EF |
|  | 0.02184 | | 0.3432 | 2.02 | 6.00 | -- | -- | -- | 0.0637 | 4 EF |
| PFUnDA | 0.00705 | | 0.0690 | 10.0 | 4.88 | 64.0 | 24.0 | 12.0 | 0.102 | 3 EF |
|  | 0.02496 | | 0.5520 | 1.3 | 6.00 | -- | -- | -- | 0.0455 | 4 EF |
| PFDoDA | 0.00765 | | 0.0300 | 23.0 | 4.88 | 64.0 | 24.0 | 12.0 | 0.26 | 3 EF |
|  | 0.0131 | | 0.0962 | 7.2 | 6.00 | -- | -- | -- | 0.1365 | 4 EF |
| PFOAB | 0.00795 * | | 0.150 | 4.62 | 5.30 | -- | -- | -- | 0.0530 | 10 LT |
| PFOAAmS | 0.005512 * | | 0.260 | 2.67 | 5.30 | -- | -- | -- | 0.0212 | 10 LT |
| **Sulfonyl Compounds - OECD Structure Category 200** | | | | | |  |  |  |  |  |
| **Sulfonic Acids - OECD Structure 202** | | | | |  |  |  |  |  |  |
| PFBS | 0.000225 | | 0.0720 | 9.60 | 4.88 | 64.0 | 24.0 | 12.0 | 0.0036 | 3 EF |
| PFHxS | 0.00150 | | 0.042 | 17.00 | 4.88 | 64.0 | 24.0 | 12.0 | 0.035 | 3 EF |
|  | 0.0434 | | 0.1517 | 4.57 | 6.00 | -- | -- | -- | 0.286 | 4 EF |
| PFOS | 0.00175 | | 0.16 | 4.387 | 0.690 | 57.2 | 9.13 | 32.7 | 0.011087 | 5 EF |
|  | 0.001598 | | 0.165 | 4.20 | 0.715 | 80.7 | 12.5 | 6.80 | 0.00968 | 5 EF |
|  | 0.00342 | | 0.231 | 3.00 | 1.15 | 69.0 | 8.63 | 22.3 | 0.01487 | 5 EF |
|  | 0.00328 | | 0.236 | 2.94 | 1.26 | 83.1 | 14.5 | 2.36 | 0.01386 | 5 EF |
|  | 0.00443 | | 0.2640 | 2.63 | 1.38 | 85.1 | 12.1 | 2.83 | 0.01679 | 5 EF |
|  | 0.00388 | | 0.265 | 2.62 | 1.66 | 79.4 | 9.93 | 10.7 | 0.01463 | 5 EF |
|  | 0.002974 | | 0.254 | 2.73 | 1.95 | 78.8 | 9.40 | 11.9 | 0.011712 | 5 EF |
|  | 0.00225 | | 0.039 | 18.00 | 4.88 | 64.0 | 24.0 | 12.0 | 0.058 | 3 EF |
|  | 0.0487 | | 0.2232 | 3.1 | 6.00 | -- | -- | -- | 0.2184 | 4 EF |
| PFDS | 0.002184 | | 0.0986 | 7.0 | 6.00 | -- | -- | -- | 0.0221 | 4 EF |
| PFOSB | 0.017808 * | | 0.420 | 1.65 | 5.30 | -- | -- | -- | 0.0424 | 10 LT |
| PFOSAmS | 0.01484 * | | 0.700 | 0.990 | 5.30 | -- | -- | -- | 0.0212 | 10 LT |
| **Sulfonamides - OECD Structure Category: 203.01** | | | | | |  |  |  |  |  |
| N-EtFOSE | 0.05595 | | 0.214 | 3.24 | 4.88 | -- | -- | -- | 0.039 | 6 EF |
| **n:2 fluorotelomer alcohol, phosphate esters (PAPs) - OECD Structure Category: 402.04** | | | | | | | | |  |  |
| 6:2 diPAP | 0.00495 | | 0.13 | 5.32 | 4.18 | -- | -- | -- | 0.0384 | 8 MG |
| **Fluorotelomer sulfonate - OECD Structure Category: 402.07** | | | | | | |  |  |  |  |
| 6:2 FTSA | 0.0925 | | 0.057 | 12.2 | 4.95 | 24.8 | 22.1 | 53.1 | 0.3425 | 7 EF |
|  |  | |  |  |  |  |  |  |  |  |
| **Aquatic oligochaetes:** *Lumbriculus variegatus* and *Limnodrilus hoffmeisteri* | | | | | | | | | | |
|  |  | |  |  |  |  |  |  |  |  |
| **Carbonyl Compounds - OECD Structure Category 100** | | | | | |  |  |  |  |  |
| **Carboxylic Acids - OECD Structure 102** | | | | |  |  |  |  |  |  |
| PFOA | 0.0384 | | 0.0403 | 17.2 | 4.34 | 38.0 | 31.0 | 31.0 | 0.950 | 2 LV |
| PFNA | 0.0262 | | 0.0163 | 42.5 | 4.34 | 38.0 | 31.0 | 31.0 | 1.60 | 2 LV |
| PFDA | 0.0278 | | 0.0274 | 25.3 | 4.34 | 38.0 | 31.0 | 31.0 | 1.02 | 2 LV |
| PFUnDA | 0.0278 | | 0.0451 | 15.4 | 4.34 | 38.0 | 31.0 | 31.0 | 0.620 | 2 LV |
| PFDoDA | 0.0257 | | 0.0463 | 15.0 | 4.34 | 38.0 | 31.0 | 31.0 | 0.550 | 2 LV |
| **Sulfonyl Compounds - OECD Structure Category 200** | | | | | |  |  |  |  |  |
| **Sulfonic Acids - OECD Structure 202** | | | | |  |  |  |  |  |  |
| PFOS | 0.0463 | | 0.0379 | 18.28 | 4.34 | 38.0 | 31.0 | 31.0 | 1.22 | 2 LV |
| PFDS | 0.0444 | | 0.0883 | 7.85 | 4.34 | 38.0 | 31.0 | 31.0 | 0.500 | 2 LV |
| **n:2 fluorotelomer alcohol, phosphate esters (PAPs) - OECD Structure Category: 402.04** | | | | | | | | |  |  |
| 8:2 diPAP | 0.00275 | | 0.114 | 6.10 | -- | -- | -- | -- | 0.0242 | 1 LH |
| * Uptake rate estimated from reported BSAF and k_e_ | | | | | | | | | | |
| Species code:  *EF=Eisenia fetida, MG= Metaphire guillelmi, LH=Limnodrilus hoffmeisteri, LV=Lumbriculus variegatus, LT = Lumbricus terrestris* | | | | | | | | | | |
| **References** | |  | | | | | | | | |
| 1 | | Chen et al. 2019 | | | | | | | | |
| 2 | | Higgins et al. 2007 | | | | | | | | |
| 3 | | Zhao et al. 2013 | | | | | | | | |
| 4 | | Rich et al. 2015 | | | | | | | | |
| 5 | | Wen et al. 2015 | | | | | | | | |
| 6 | | Zhao et al. 2016 | | | | | | | | |
| 7 | | Zhao et al. 2021 | | | | | | | | |
| 8 | | Zhu et al. 2021 | | | | | | | | |
| 10 | | Jin et al. 2020 | | | | | | | | |

| **Table S7. Soil properties reported by study** |  |  |  |  |  |  |  |
| --- | --- | --- | --- | --- | --- | --- | --- |
| **Study** | **Soil Type/Study soil name** | **sand** | **silt** | **clay** | **Organic Matter** | **Organic Carbon** | **pH** |
|  |  | % | % | % | % | % |  |
| Bräunig et al. 2019 | AFFF - Soil A | 63 | 25 | 20 | -- | 2.9 | 6.3 |
| Bräunig et al. 2019 | AFFF - Soil B | 92 | 4 | 5 | -- | 0.5 | 8.5 |
| Bräunig et al. 2019 | AFFF - Soil C | 54 | 16 | 33 | -- | 1.1 | 6.9 |
| Das, Megharaj, and Naidu 2015 | sandy clay loam | 52.63 | 25.62 | 21.74 | -- | 0.96 | 4.8 |
| He, Megharaj, and Naidu 2016 | Edinburgh, South Australia (EB) | 48.7 | 31.1 | 21.2 | 1.6 | 0.8 | 8.08 |
| He, Megharaj, and Naidu 2016 | Williamston, New South Wales (WT) | 84.8 | 8.6 | 6.6 | 2.53 | 1.265 | 5.99 |
| Jarjour et al. 2022 | sandy loam | 68.3 | 16.4 | 15.3 | 2.5 | 1.96 | 7.2 |
| Karnjanapiboonwong et al. 2018 | sandy loam | 74 | 10 | 16 | 0 | 1.3 | 8.3 |
| Sobhani et al. 2021 | University of Newcastle campus | 38 | 33 | 29 | -- | -- | 6.5 |
| Wang et al. 2022 | -- | 70 | 10 | 20 | -- | -- | 6.5 |
| Wen et al. 2015 | Control | 32.1 | 23.1 | 35.1 | 1.2 | -- | 7.36 |
| Wen et al. 2015 | 1-Dezhou | 32.7 | 57.2 | 9.13 | 1.38 | -- | 8.05 |
| Wen et al. 2015 | 2-Changping | 6.8 | 80.7 | 12.5 | 1.43 | -- | 8.21 |
| Wen et al. 2015 | 3-Changping | 22.3 | 69 | 8.63 | 2.29 | -- | 8.43 |
| Wen et al. 2015 | 4-Changping | 2.36 | 83.1 | 14.5 | 2.52 | -- | 8.15 |
| Wen et al. 2015 | 5-Changping | 2.83 | 85.1 | 12.1 | 2.76 | -- | 8.2 |
| Wen et al. 2015 | 6-Dezhou | 10.7 | 79.4 | 9.93 | 3.32 | -- | 8.26 |
| Wen et al. 2015 | 7-Dezhou | 11.9 | 78.8 | 9.4 | 3.9 | -- | 8.13 |
| Zhao et al. 2013 | loamy | 12 | 64 | 24 | 4.11 | 2.055 | 7.67 |
| Zhao et al. 2014 | loamy | 12 | 64 | 24 | 4.11 | 2.055 | 7.67 |
| Zhao et al. 2016 | loamy | 12 | 64 | 24 | 4.11 | 2.055 | 7.67 |
| Zhao et al. 2018 | Agricultural | 53.1 | 24.8 | 22.1 | -- | 2.84 | 7.55 |
| Zhao and Zhu 2017 | loamy | 12 | 64 | 24 | 4.11 | 2.055 | 7.67 |

**Carboxylic Acids**

**
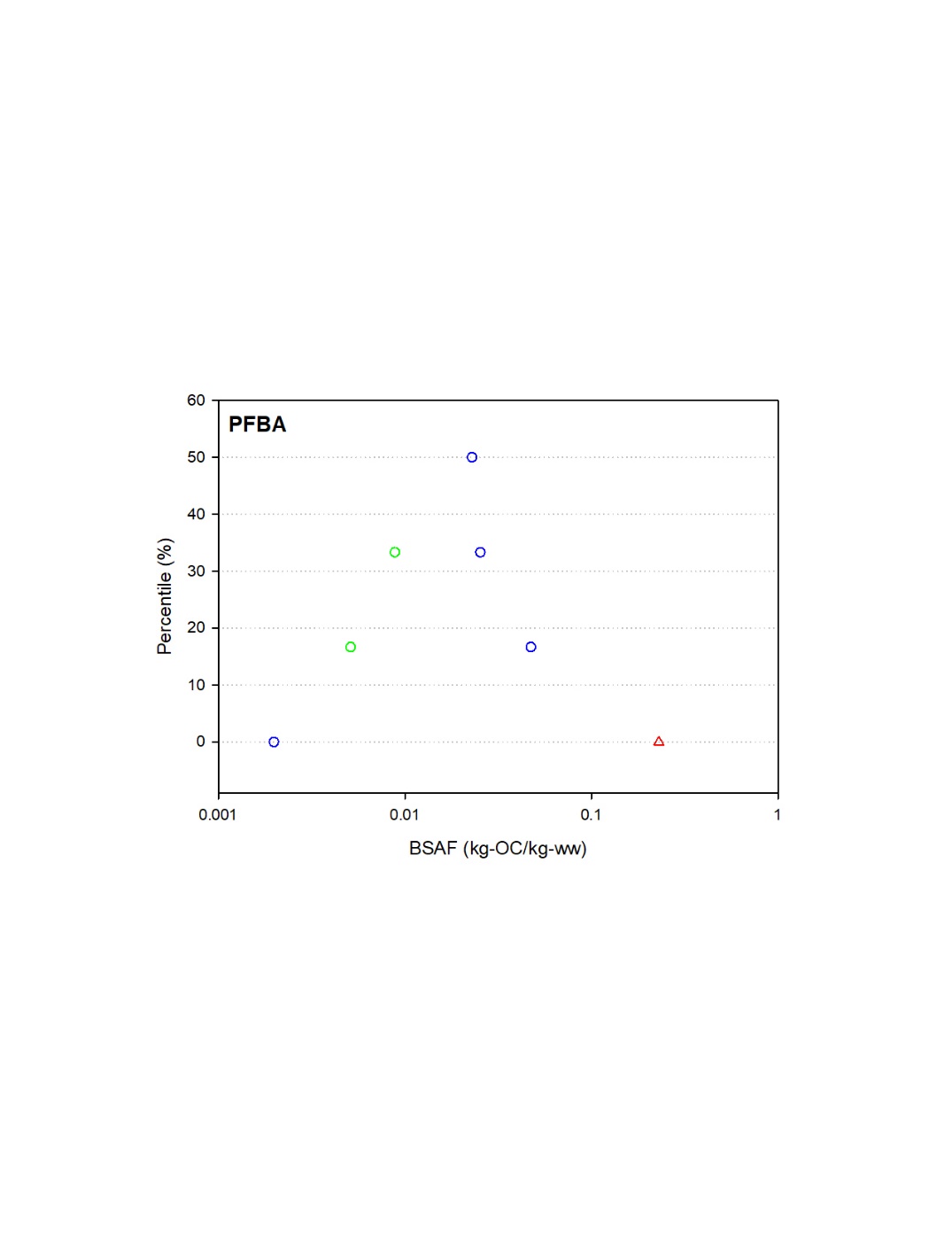
**


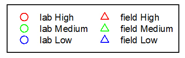


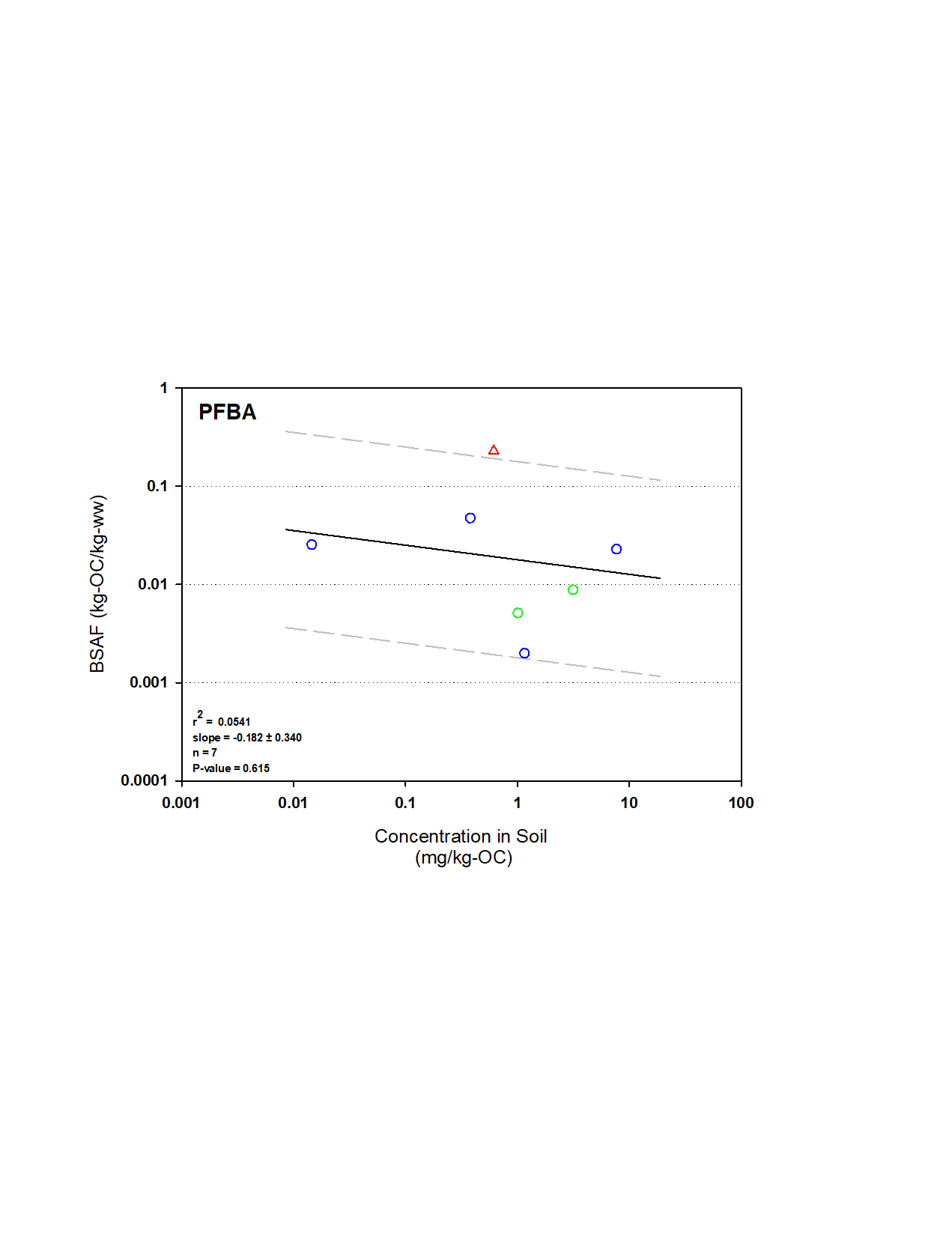


Figure S1A. For PFBA: Distribution plot of BSAFs and plot of BSAF (kg-OC/kg-ww) vs concentration in soil (mg/kg-OC). Measurement location: laboratory (circle) and field (triangle). Study quality ranking: high (red), medium (green) and low (blue). Regression line (solid) and statistics (slope ± standard error) along with lines 10-fold higher and lower are shown in BSAF vs concentration soil plot.


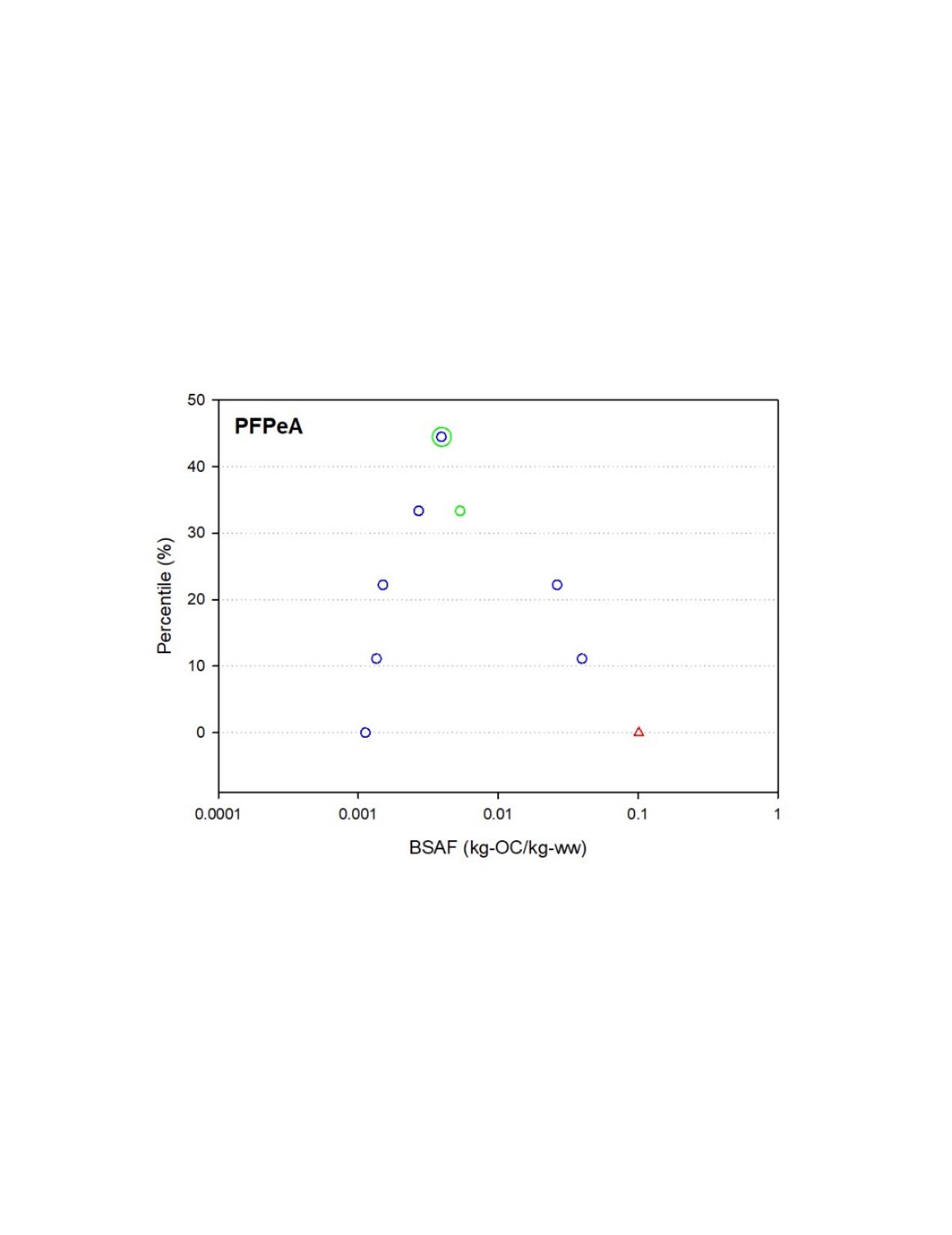


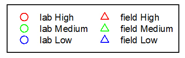


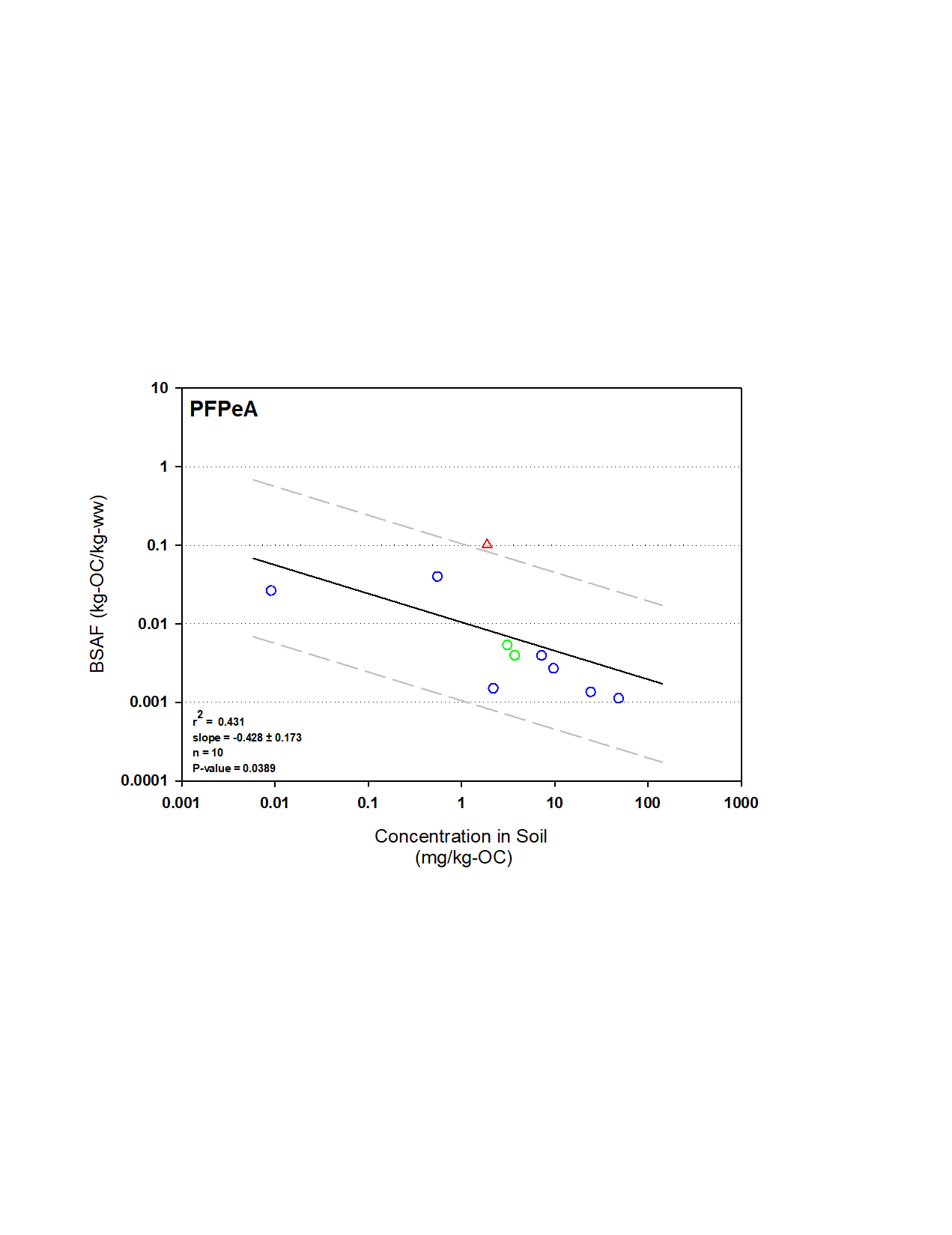


Figure S1B. For PFPeA: Distribution plot of BSAFs and plot of BSAF (kg-OC/kg-ww) vs concentration in soil (mg/kg-OC). Measurement location: laboratory (circle) and field (triangle). Study quality ranking: high (red), medium (green) and low (blue). Regression line (solid) and statistics (slope ± standard error) along with lines 10-fold higher and lower are shown in BSAF vs concentration soil plot. When data points overlap, second point is drawn a bit larger.


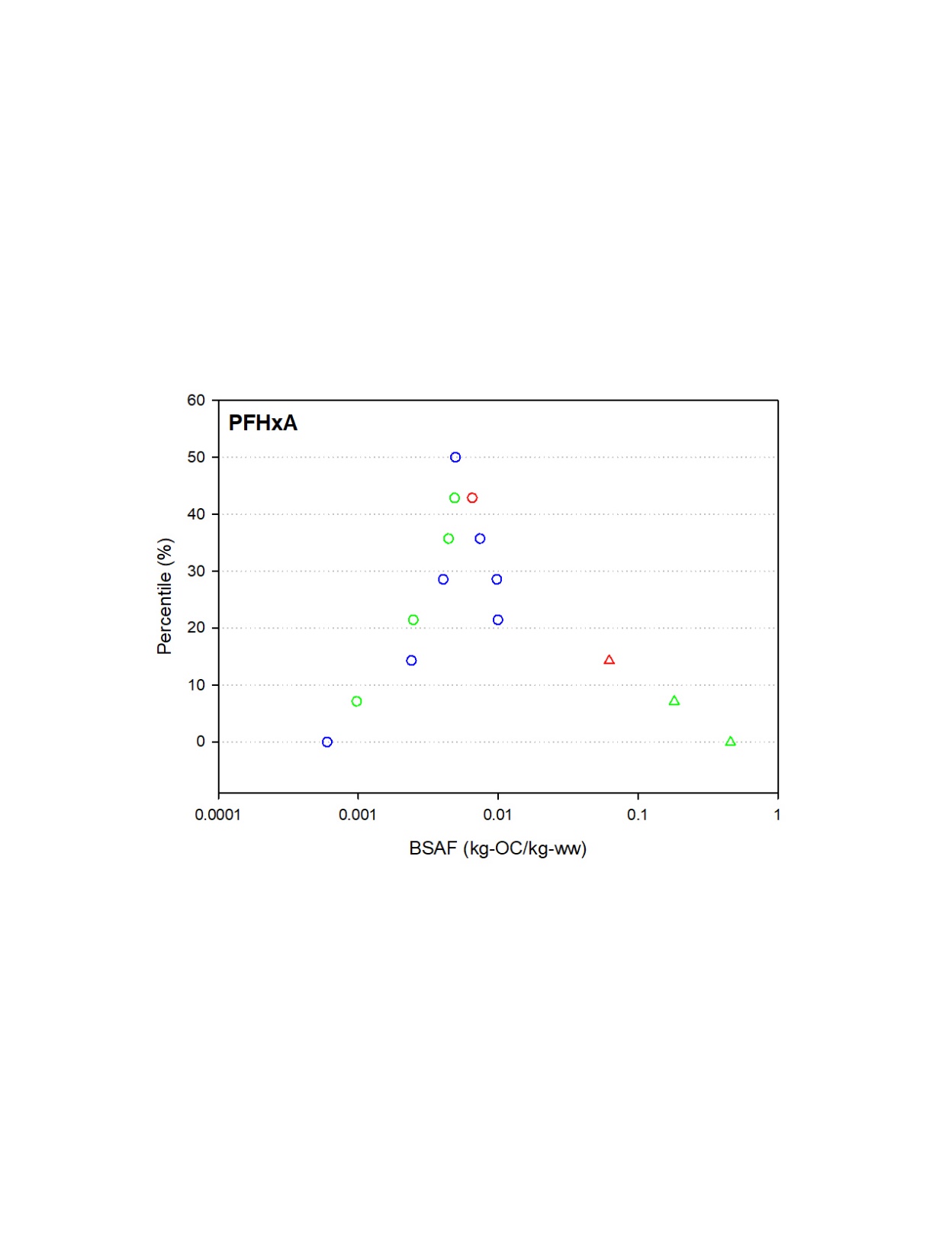


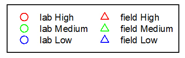


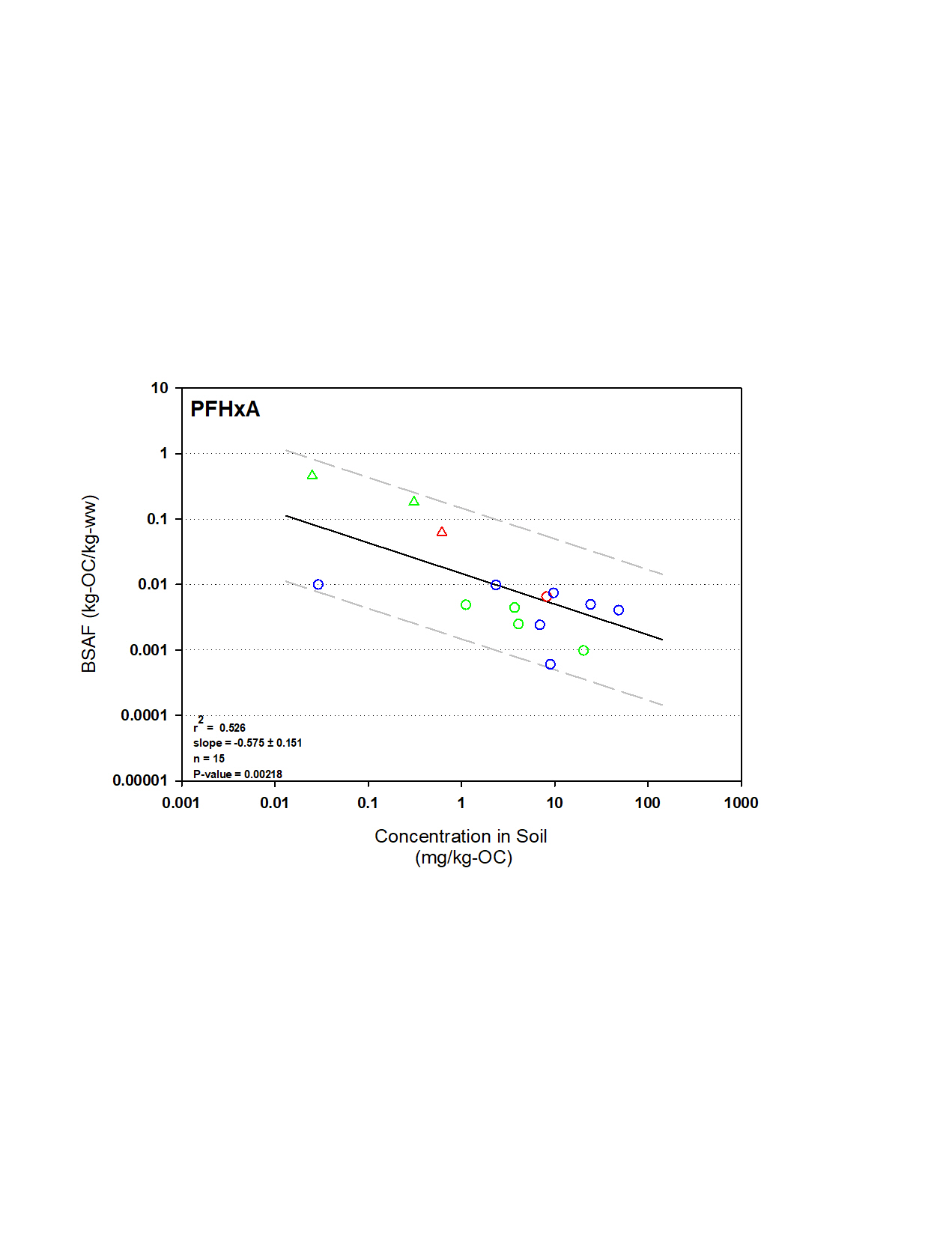


Figure S1C. For PFHxA: Distribution plot of BSAFs and plot of BSAF (kg-OC/kg-ww) vs concentration in soil (mg/kg-OC). Measurement location: laboratory (circle) and field (triangle). Study quality ranking:high (red), medium (green) and low (blue). Regression line (solid) and statistics (slope ± standard error) along with lines 10-fold higher and lower are shown in BSAF vs concentration soil plot.


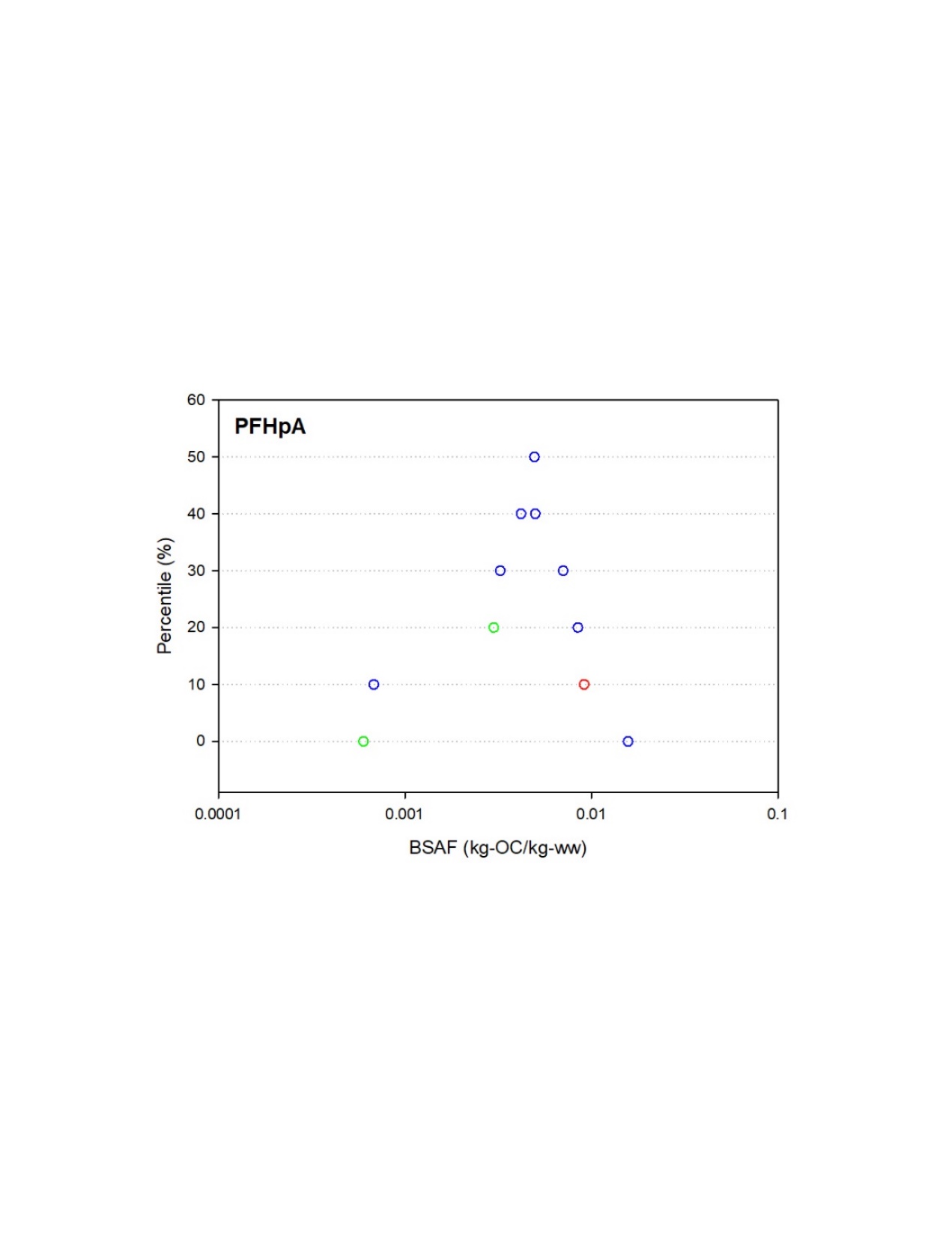


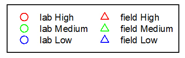


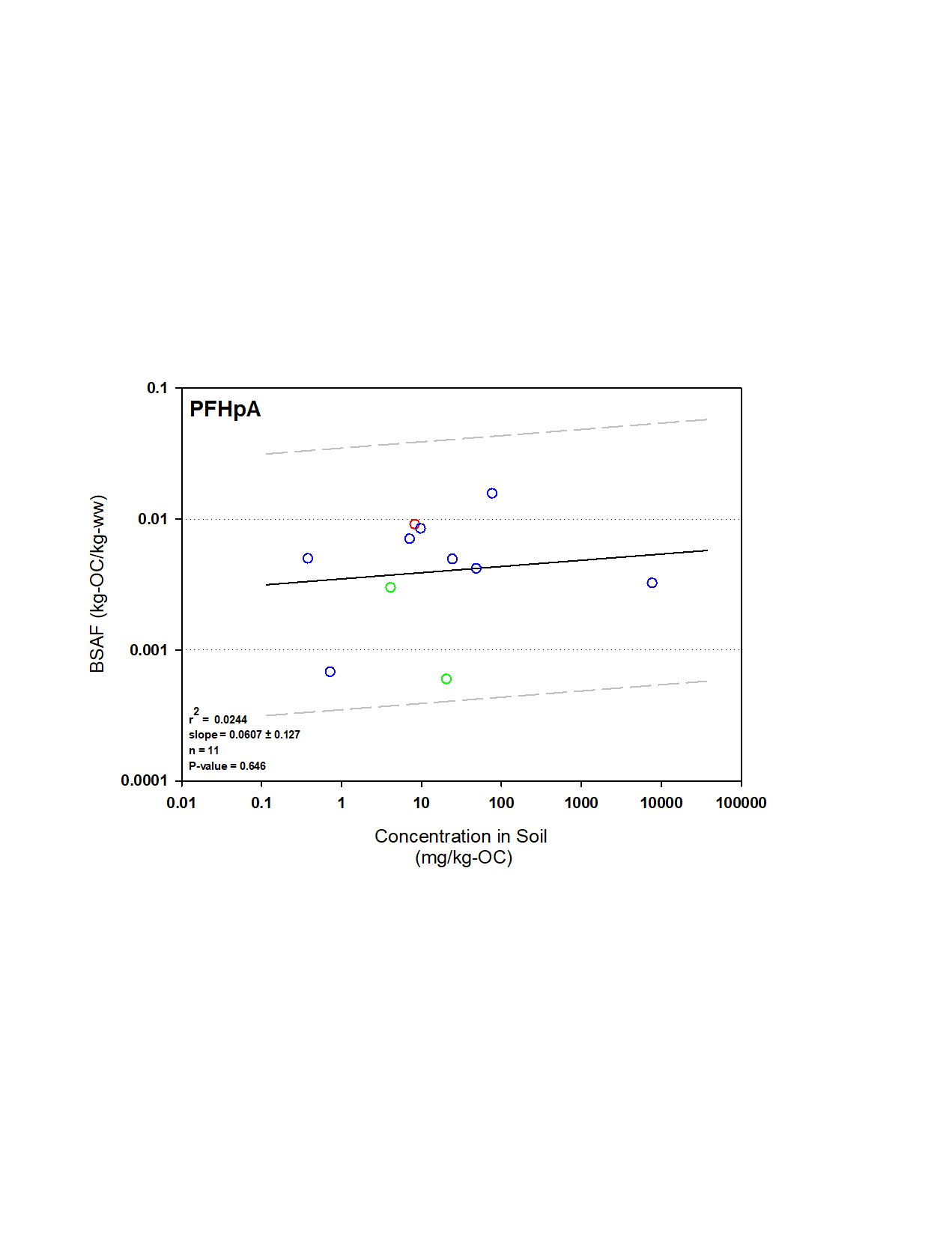


Figure S1D. For PFHpA: Distribution plot of BSAFs and plot of BSAF (kg-OC/kg-ww) vs concentration in soil (mg/kg-OC). Measurement location: laboratory (circle) and field (triangle). Study quality ranking: high (red), medium (green) and low (blue). Regression line (solid) and statistics (slope ± standard error) along with lines 10-fold higher and lower are shown in BSAF vs concentration soil plot.


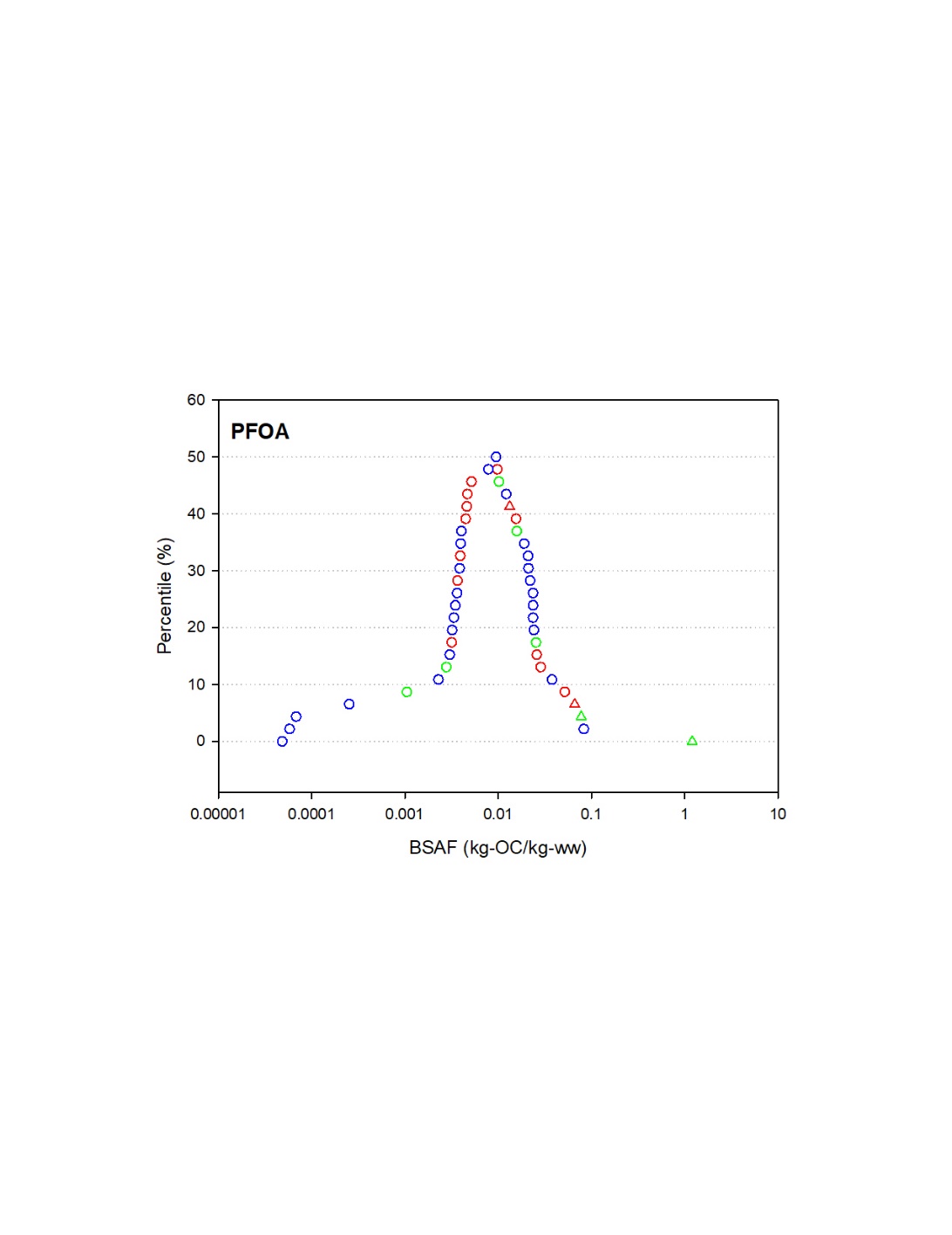


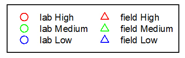


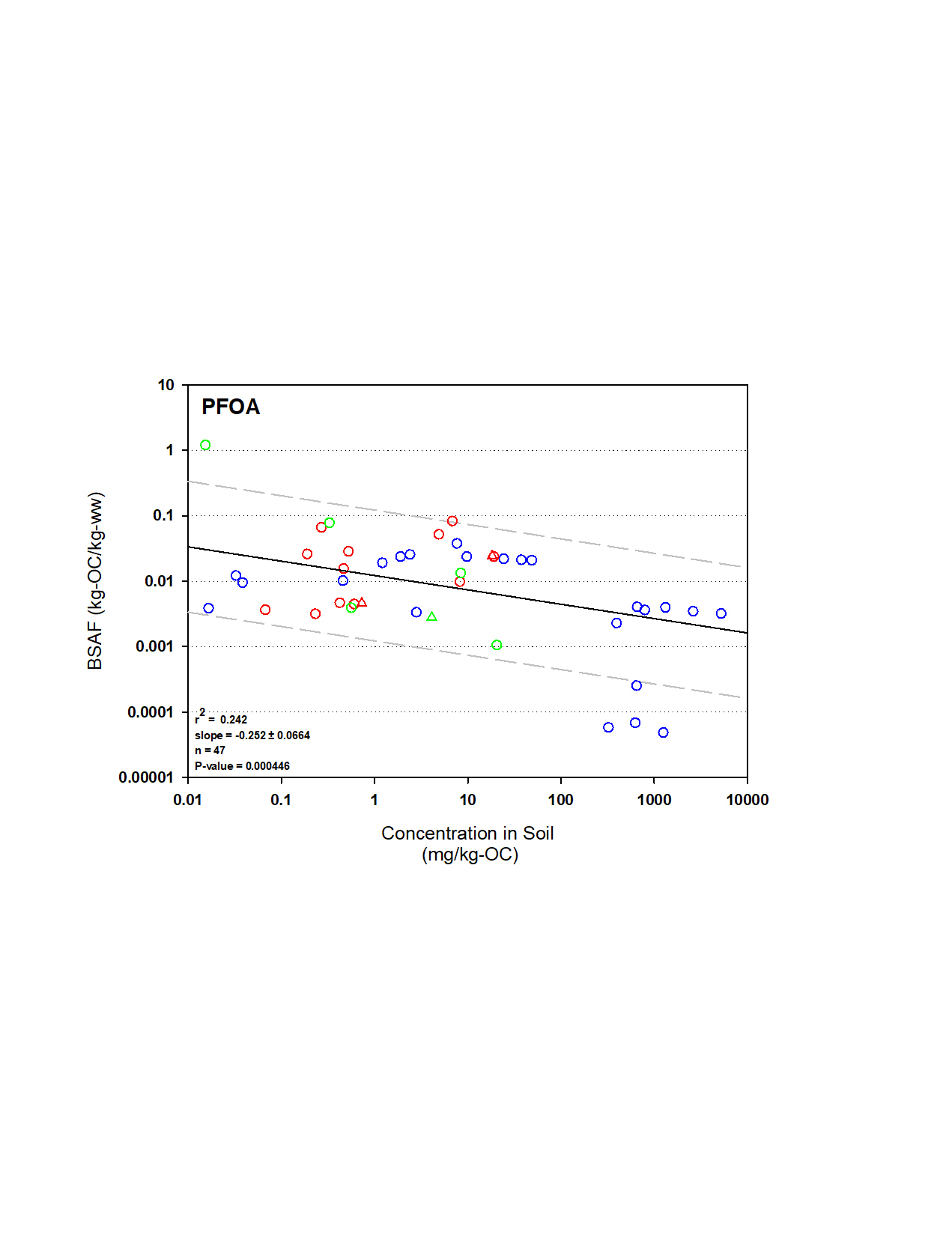


Figure S1E. For PFOA: Distribution plot of BSAFs and plot of BSAF (kg-OC/kg-ww) vs concentration in soil (mg/kg-OC). Measurement location: laboratory (circle) and field (triangle). Study quality ranking: high (red), medium (green) and low (blue). Regression line (solid) and statistics (slope ± standard error) along with lines 10-fold higher and lower are shown in BSAF vs concentration soil plot.


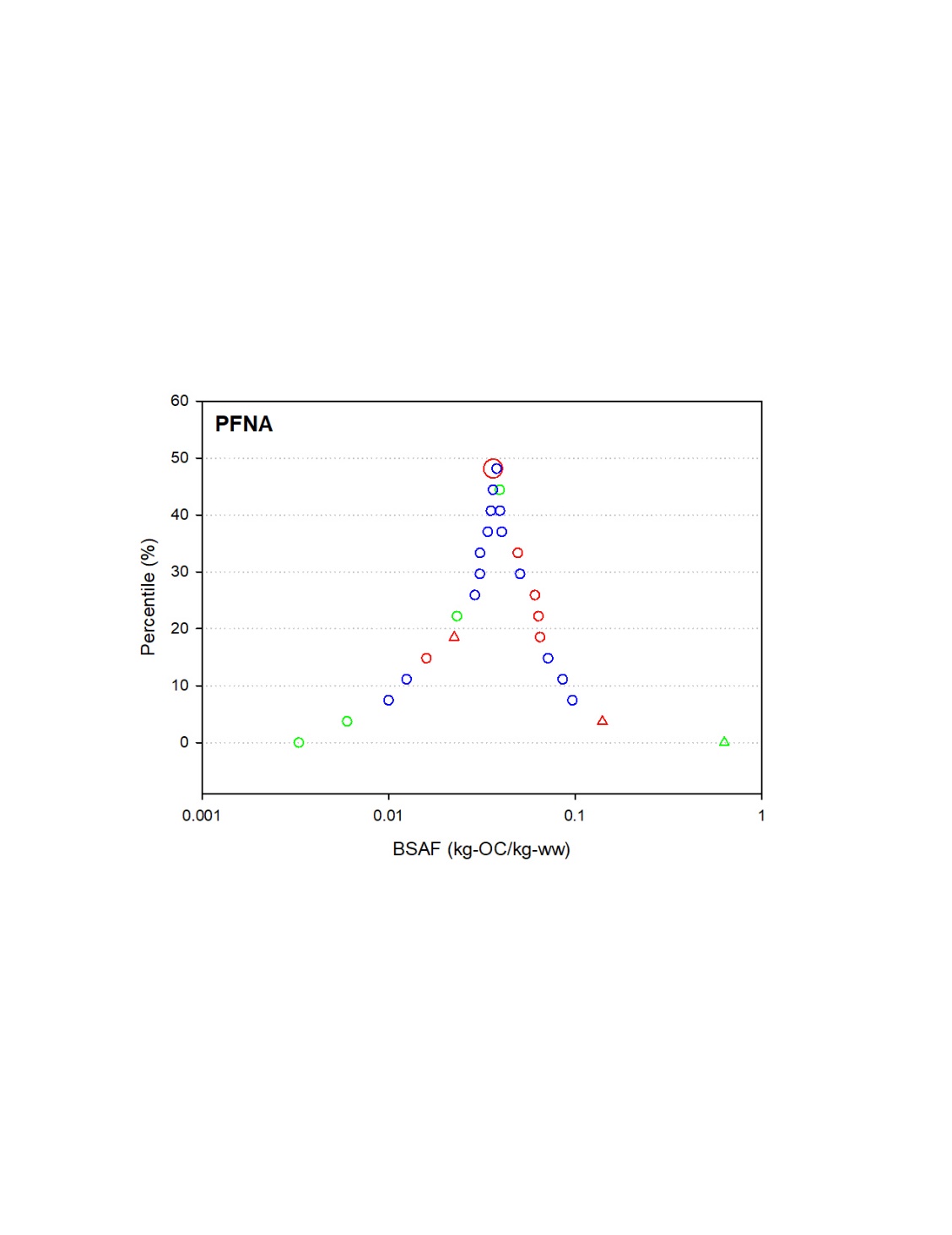


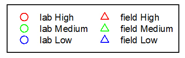


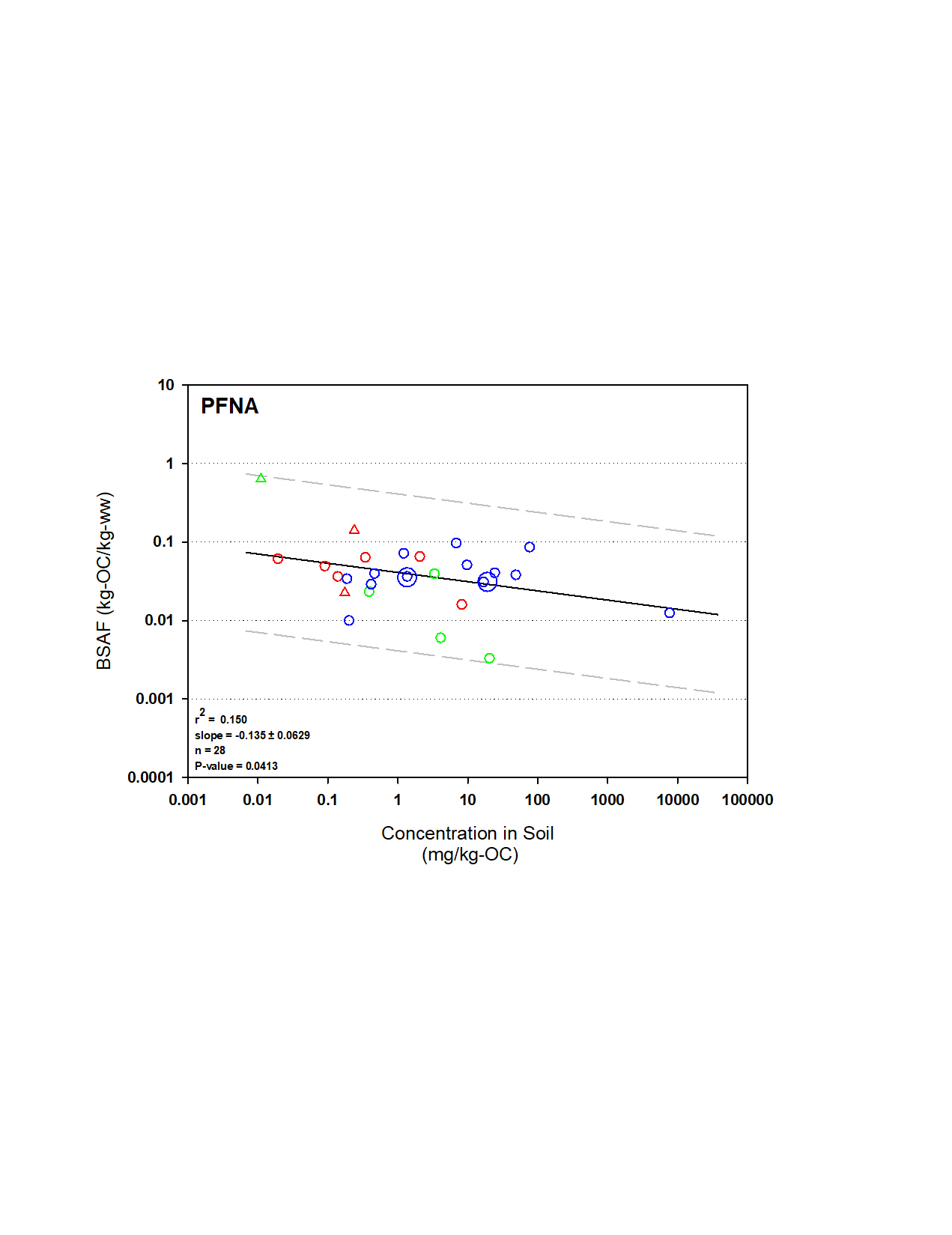


Figure S1F. For PFNA: Distribution plot of BSAFs and plot of BSAF (kg-OC/kg-ww) vs concentration in soil (mg/kg-OC). Measurement location: laboratory (circle) and field (triangle). Study quality ranking: high (red), medium (green) and low (blue). Regression line (solid) and statistics (slope ± standard error) along with lines 10-fold higher and lower are shown in BSAF vs concentration soil plot. When data points overlap, second point is drawn a bit larger.


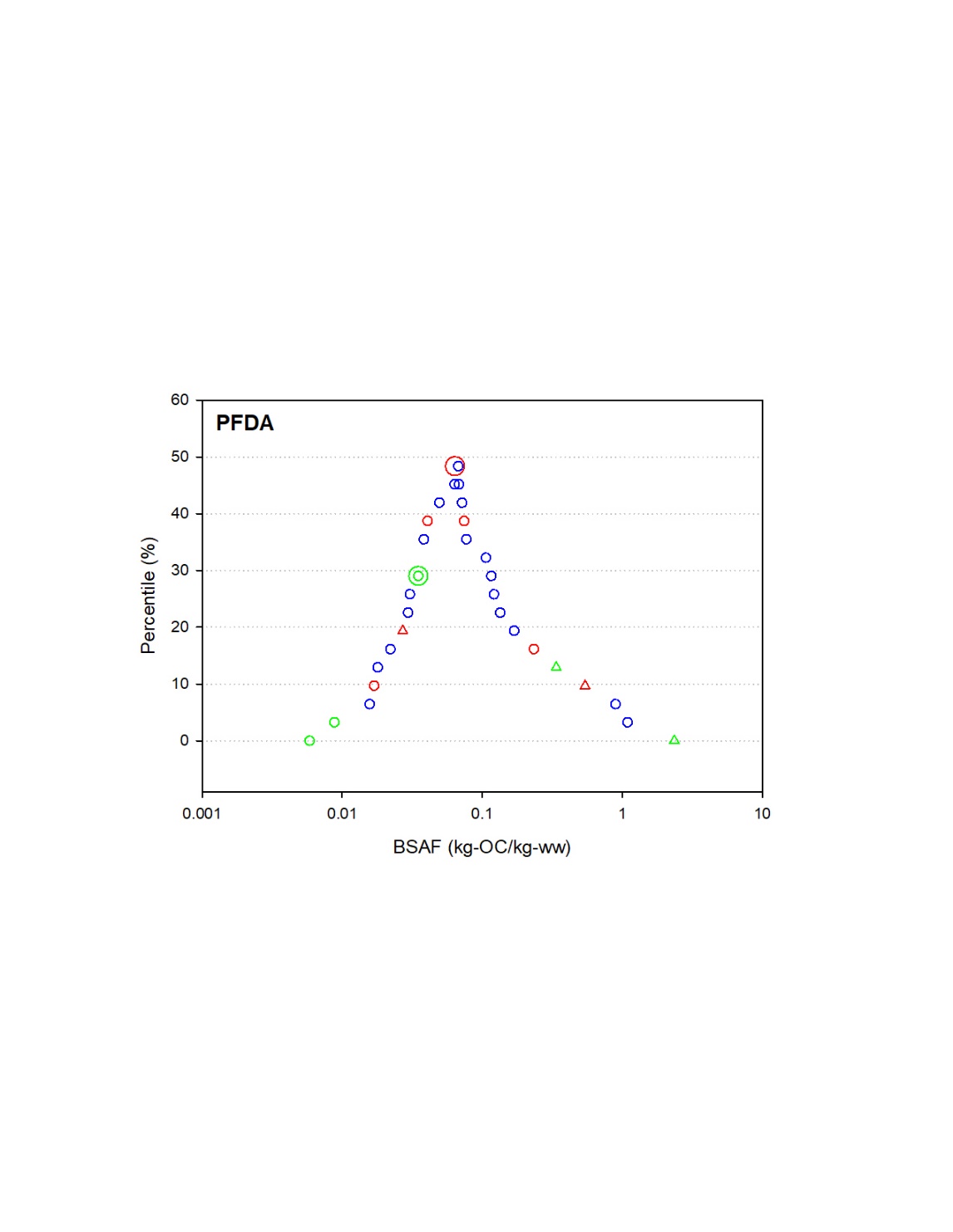


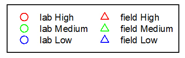


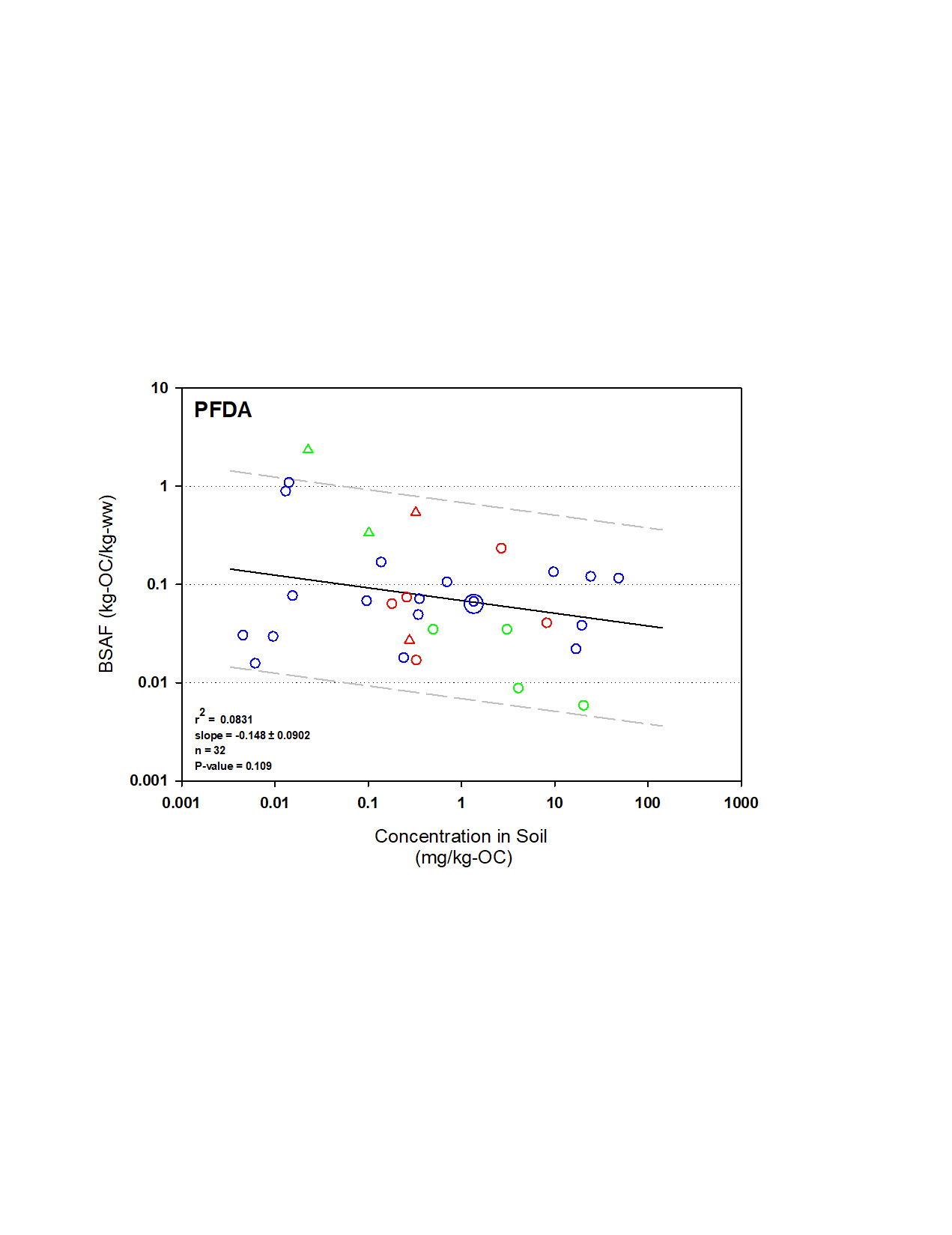


Figure S1G. For PFDA: Distribution plot of BSAFs and plot of BSAF (kg-OC/kg-ww) vs concentration in soil (mg/kg-OC). Measurement location: laboratory (circle) and field (triangle). Study quality ranking: high (red), medium (green) and low (blue). Regression line (solid) and statistics (slope ± standard error) along with lines 10-fold higher and lower are shown in BSAF vs concentration soil plot. When data points overlap, second point is drawn a bit larger.


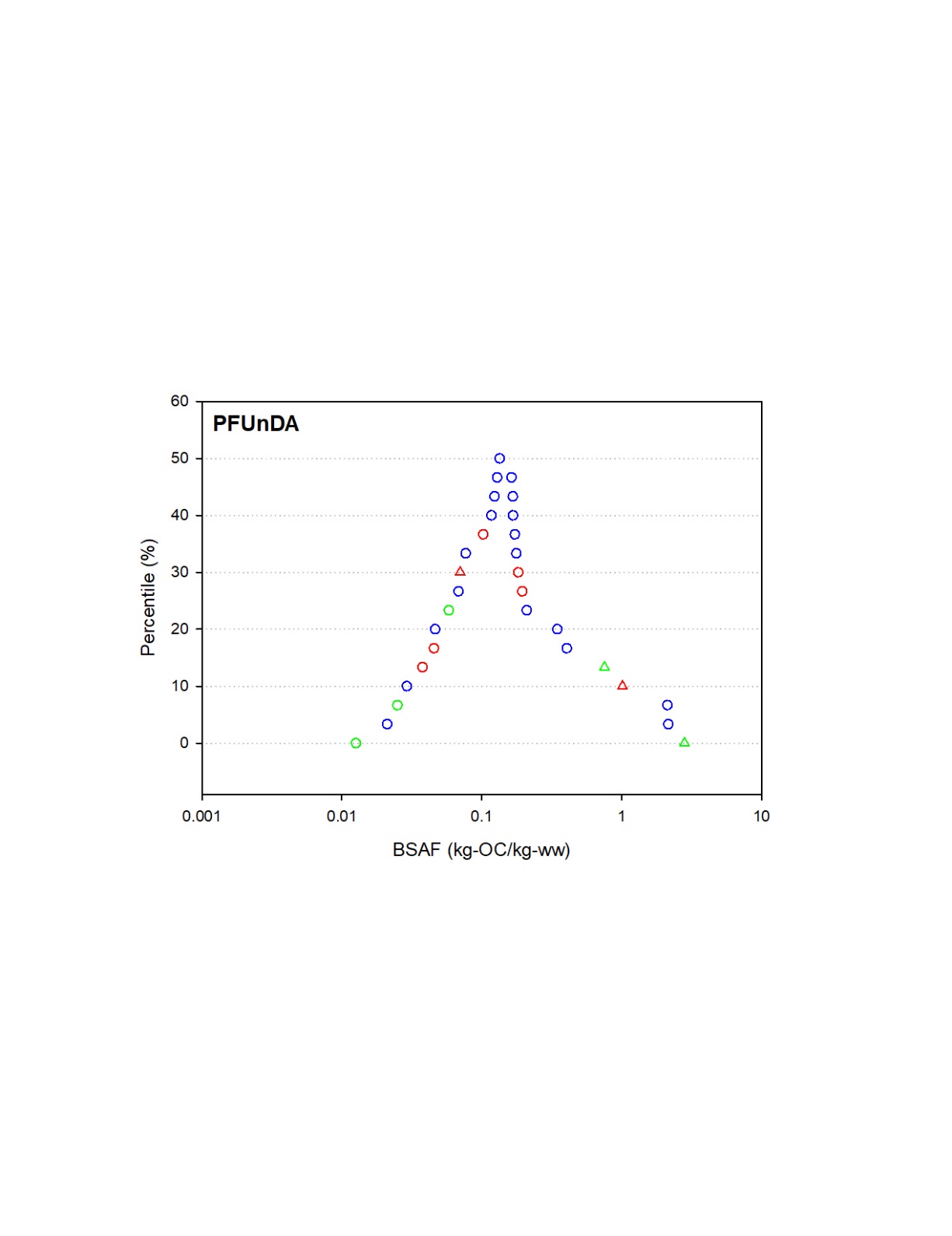


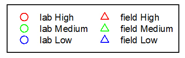

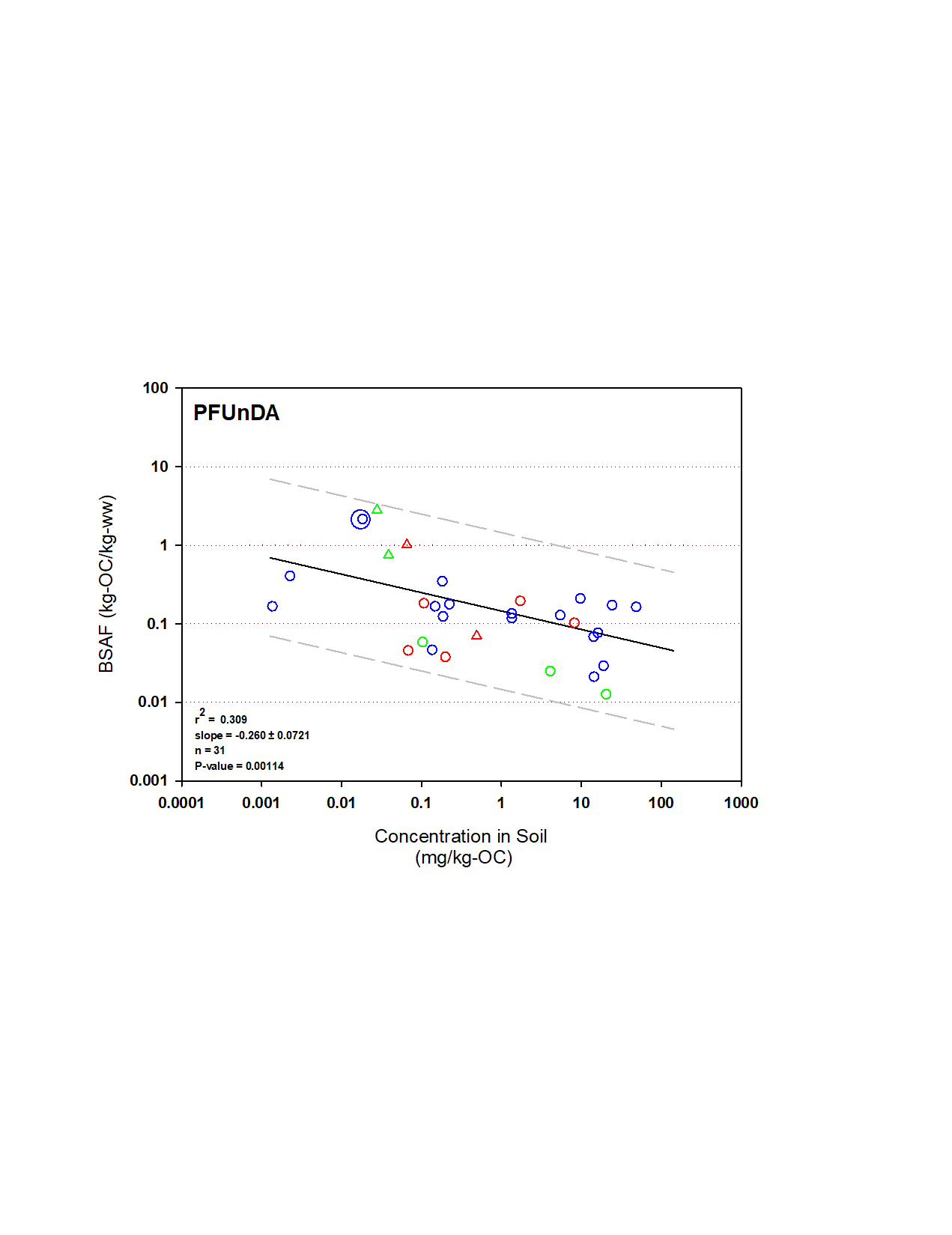


Figure S1H. For PFUnDA: Distribution plot of BSAFs and plot of BSAF (kg-OC/kg-ww) vs concentration in soil (mg/kg-OC). Measurement location: laboratory (circle) and field (triangle). Study quality ranking: high (red), medium (green) and low (blue). Regression line (solid) and statistics (slope ± standard error) along with lines 10-fold higher and lower are shown in BSAF vs concentration soil plot.


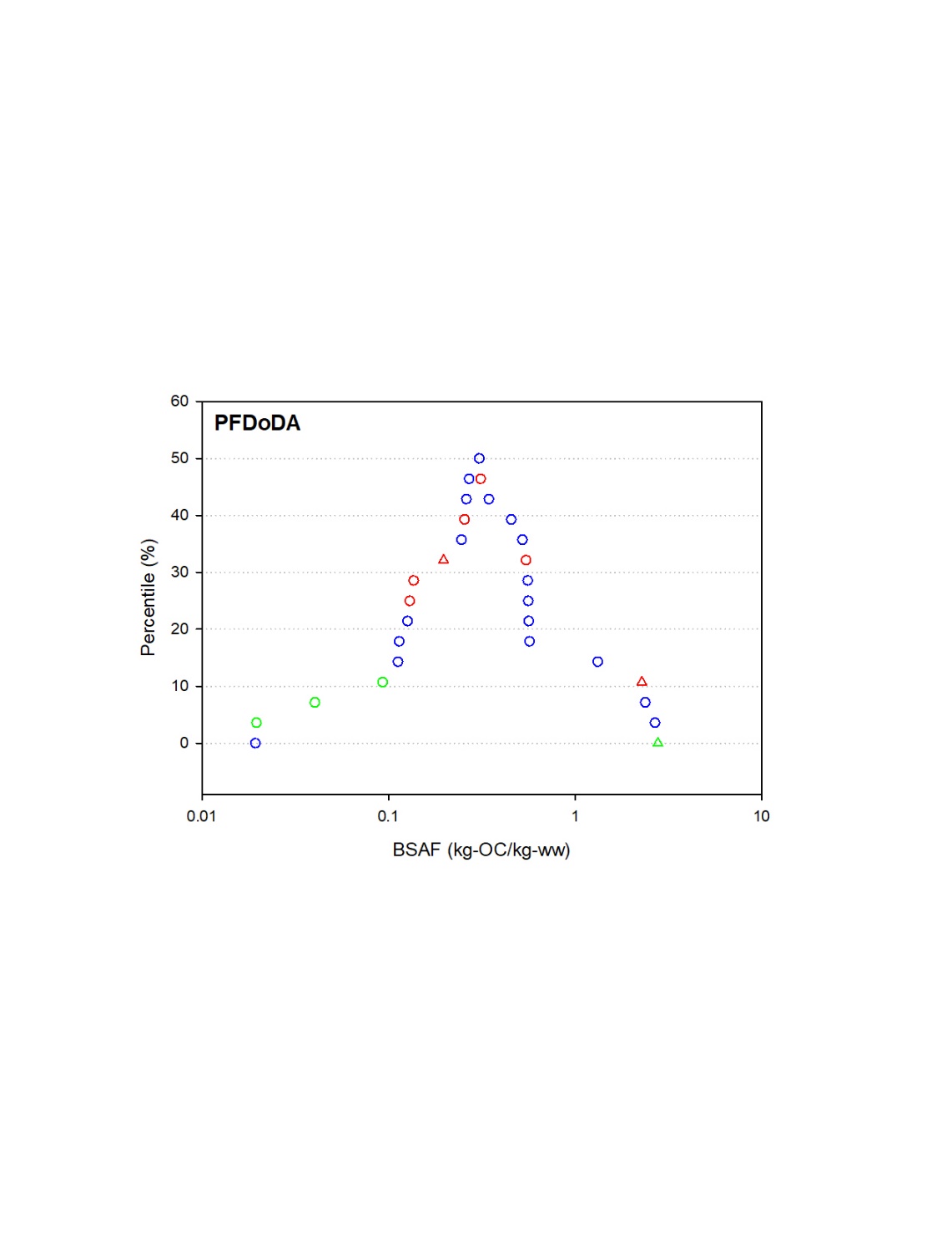


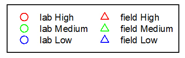


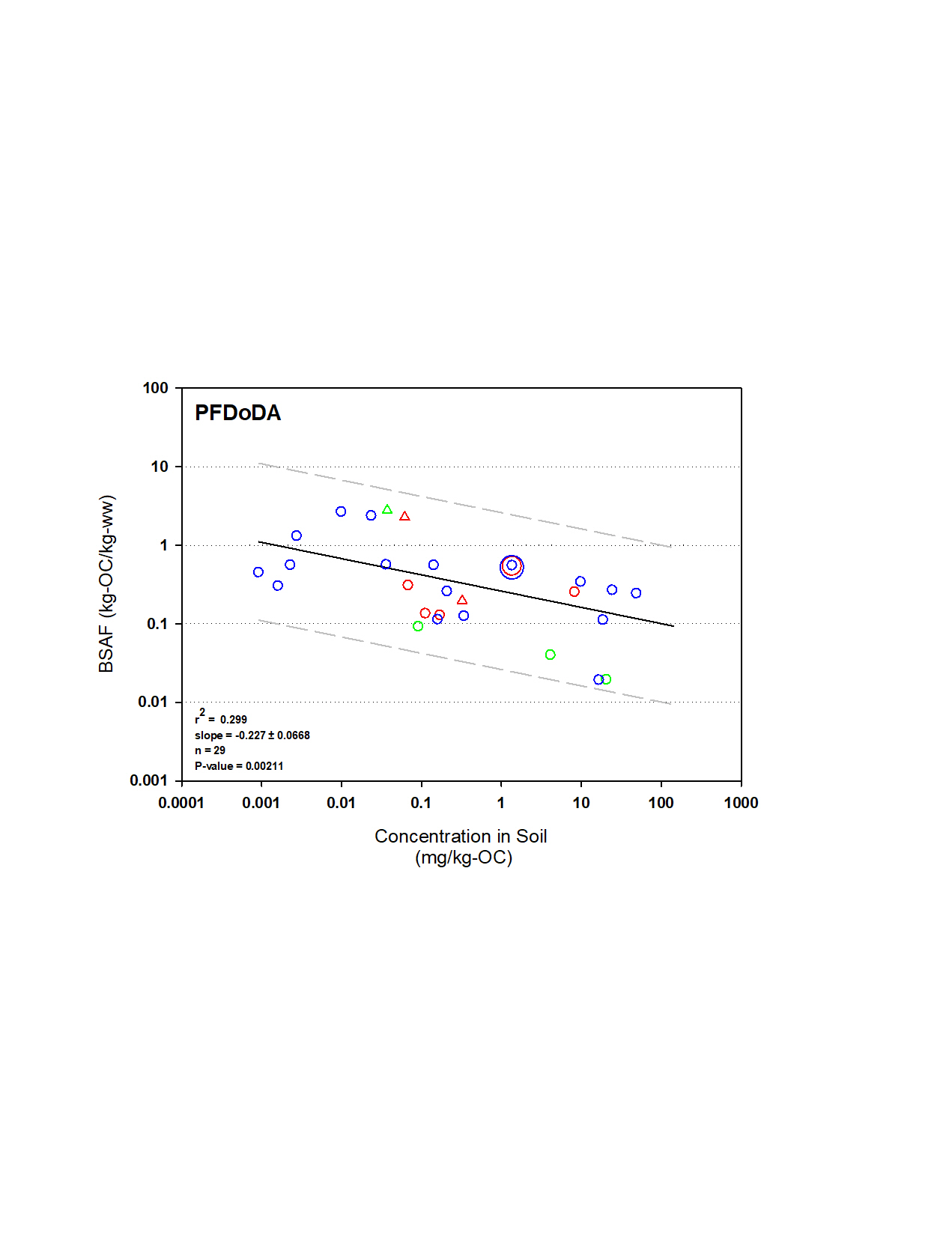


Figure S1I. For PFDoDA: Distribution plot of BSAFs and plot of BSAF (kg-OC/kg-ww) vs concentration in soil (mg/kg-OC). Measurement location: laboratory (circle) and field (triangle). Study quality ranking: high (red), medium (green) and low (blue). Regression line (solid) and statistics (slope ± standard error) along with lines 10-fold higher and lower are shown in BSAF vs concentration soil plot.


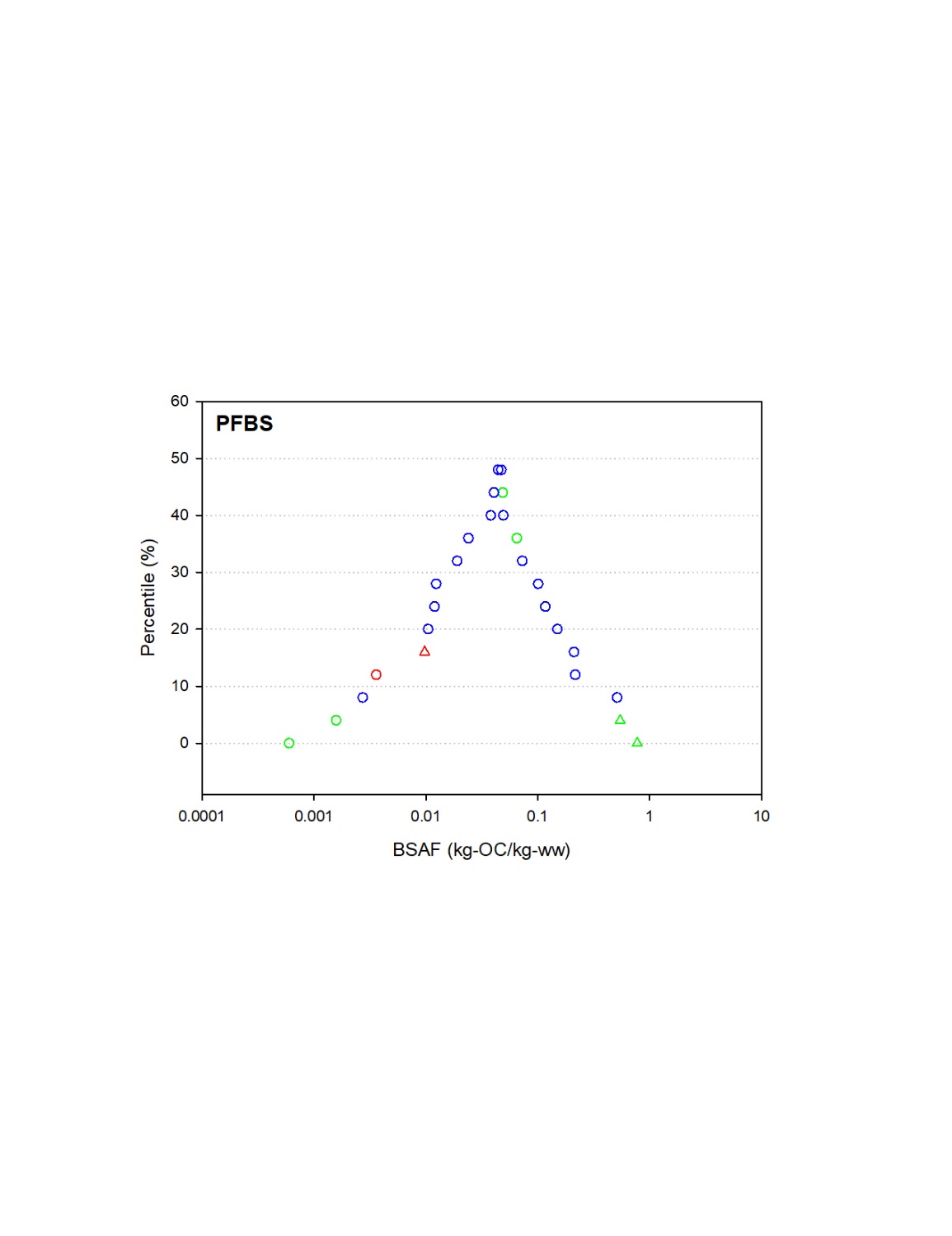


**Sulfonic Acids**


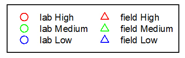


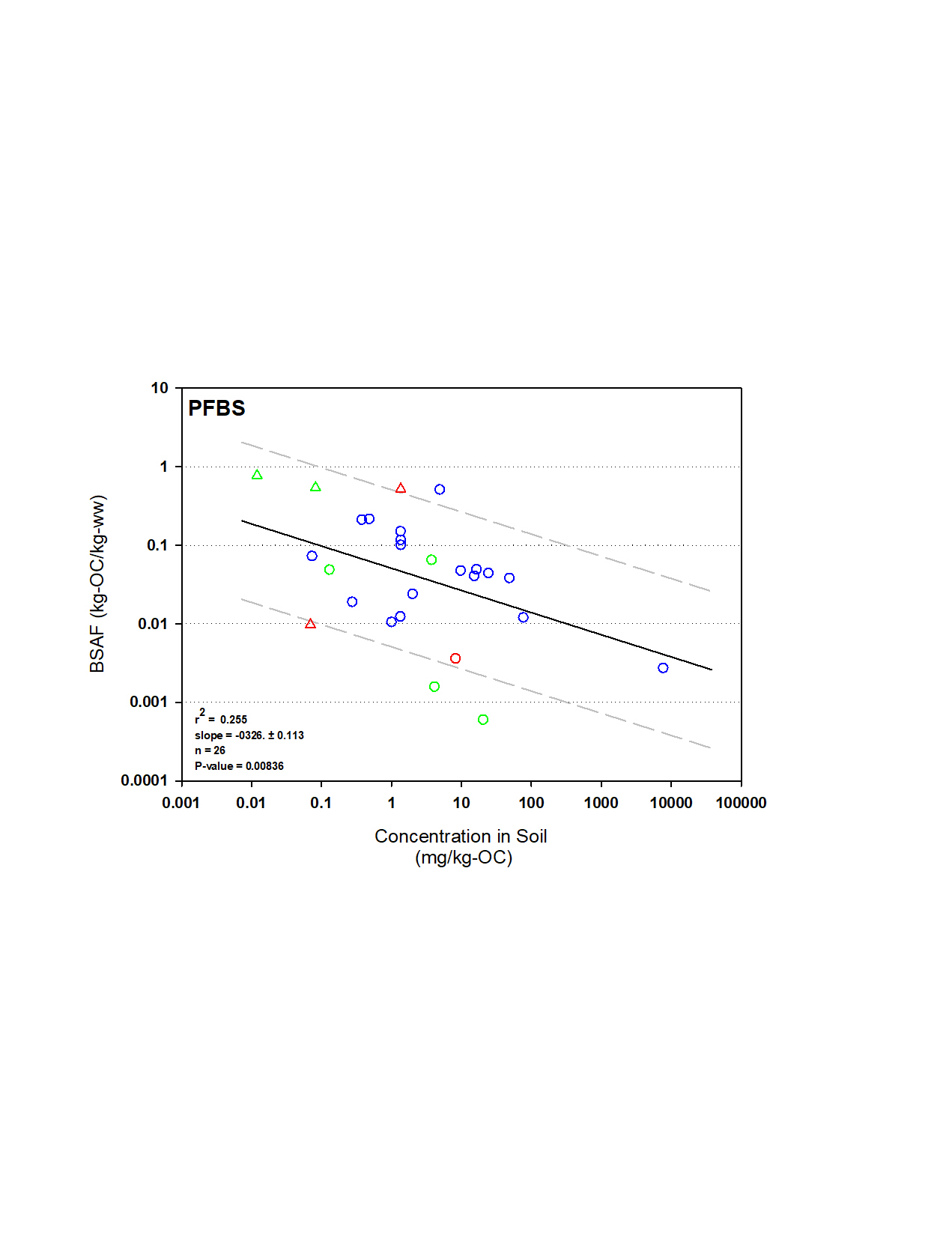


Figure S1J. For PFBS: Distribution plot of BSAFs and plot of BSAF (kg-OC/kg-ww) vs concentration in soil (mg/kg-OC). Measurement location: laboratory (circle) and field (triangle). Study quality ranking: high (red), medium (green) and low (blue). Regression line (solid) and statistics (slope ± standard error) along with lines 10-fold higher and lower are shown in BSAF vs concentration soil plot.


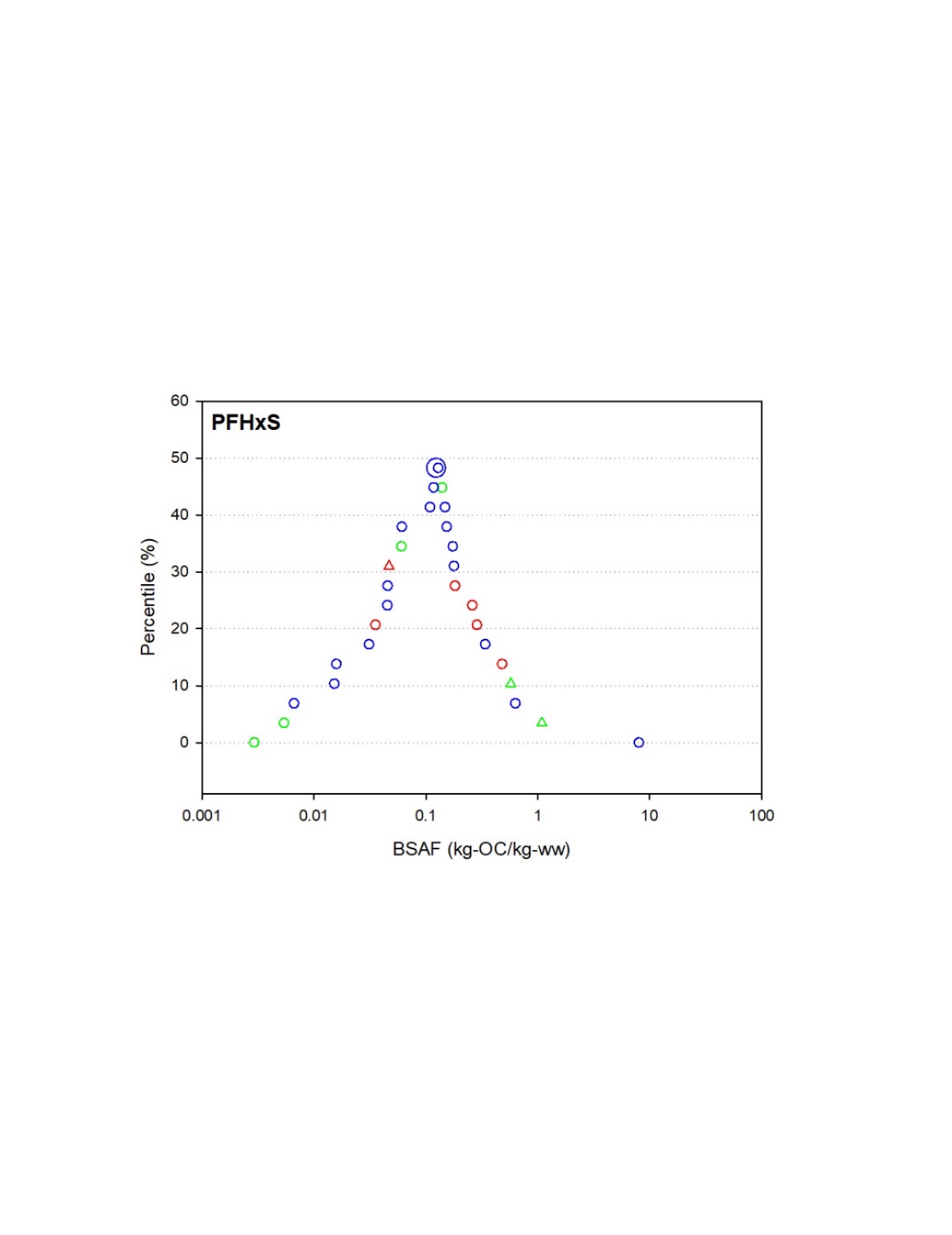


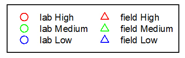


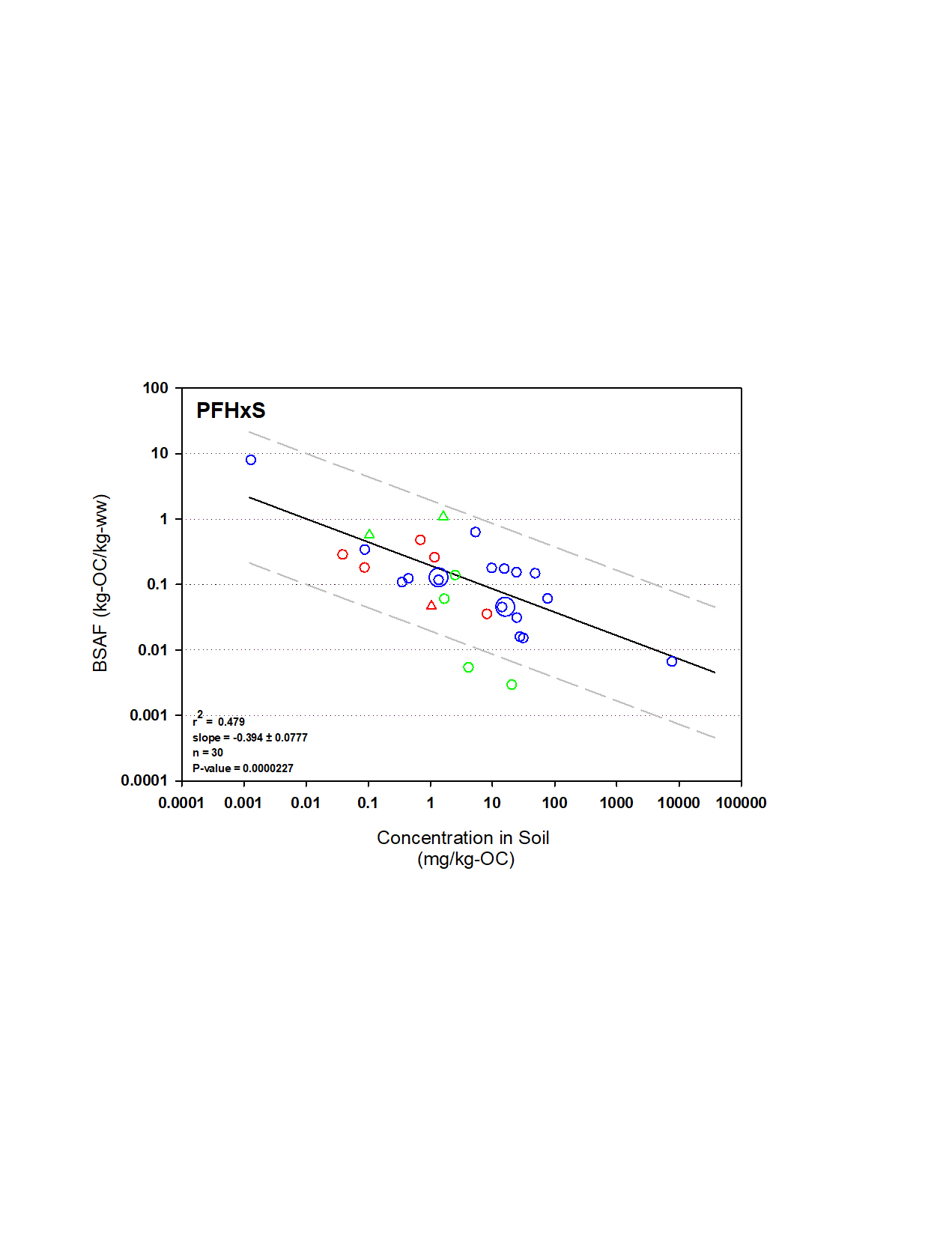


Figure S1K. For PFHxS: Distribution plot of BSAFs and plot of BSAF (kg-OC/kg-ww) vs concentration in soil (mg/kg-OC). Measurement location: laboratory (circle) and field (triangle). Study quality ranking: high (red), medium (green) and low (blue). Regression line (solid) and statistics (slope ± standard error) along with lines 10-fold higher and lower are shown in BSAF vs concentration soil plot. When data points overlap, second point is drawn a bit larger.


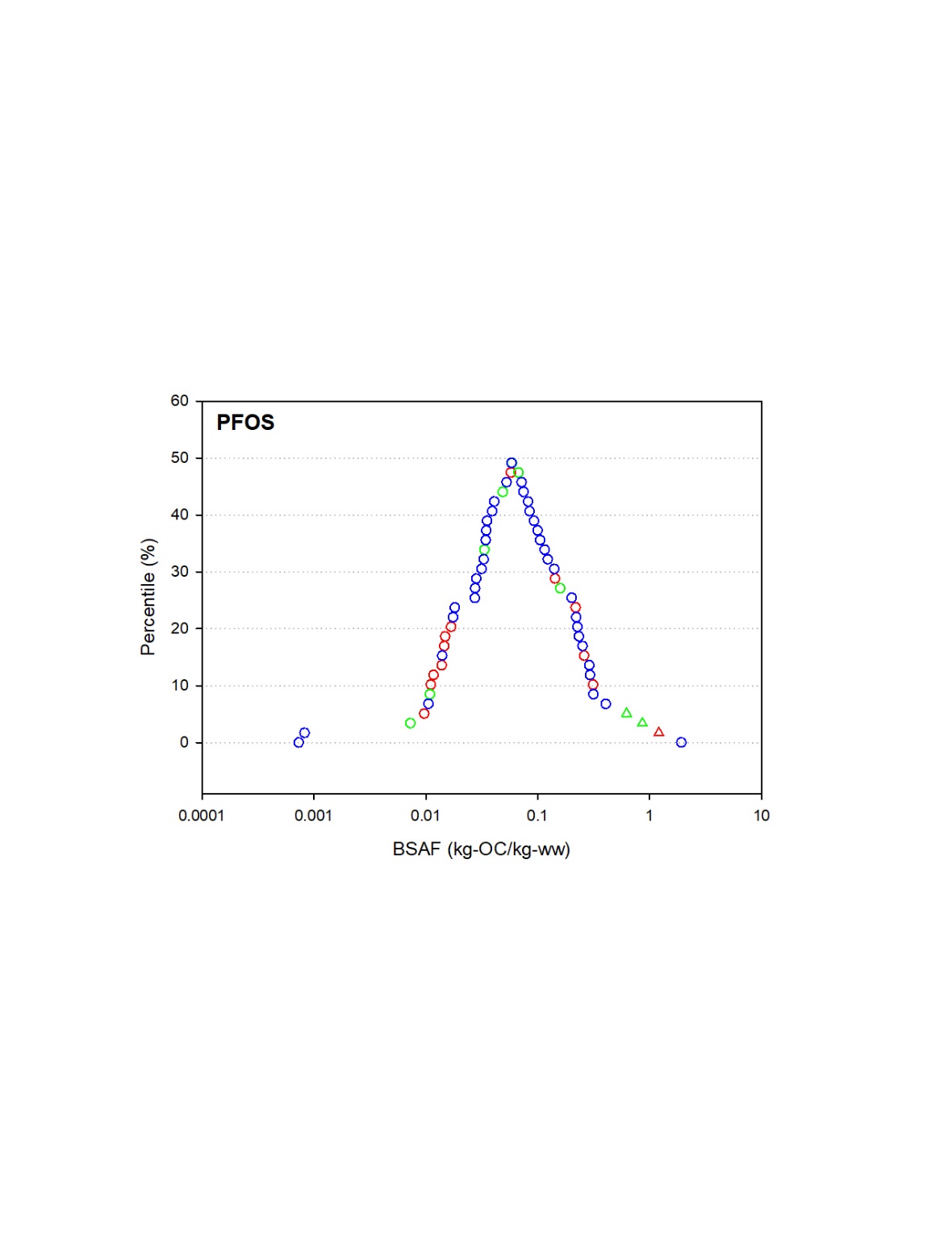


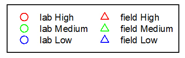


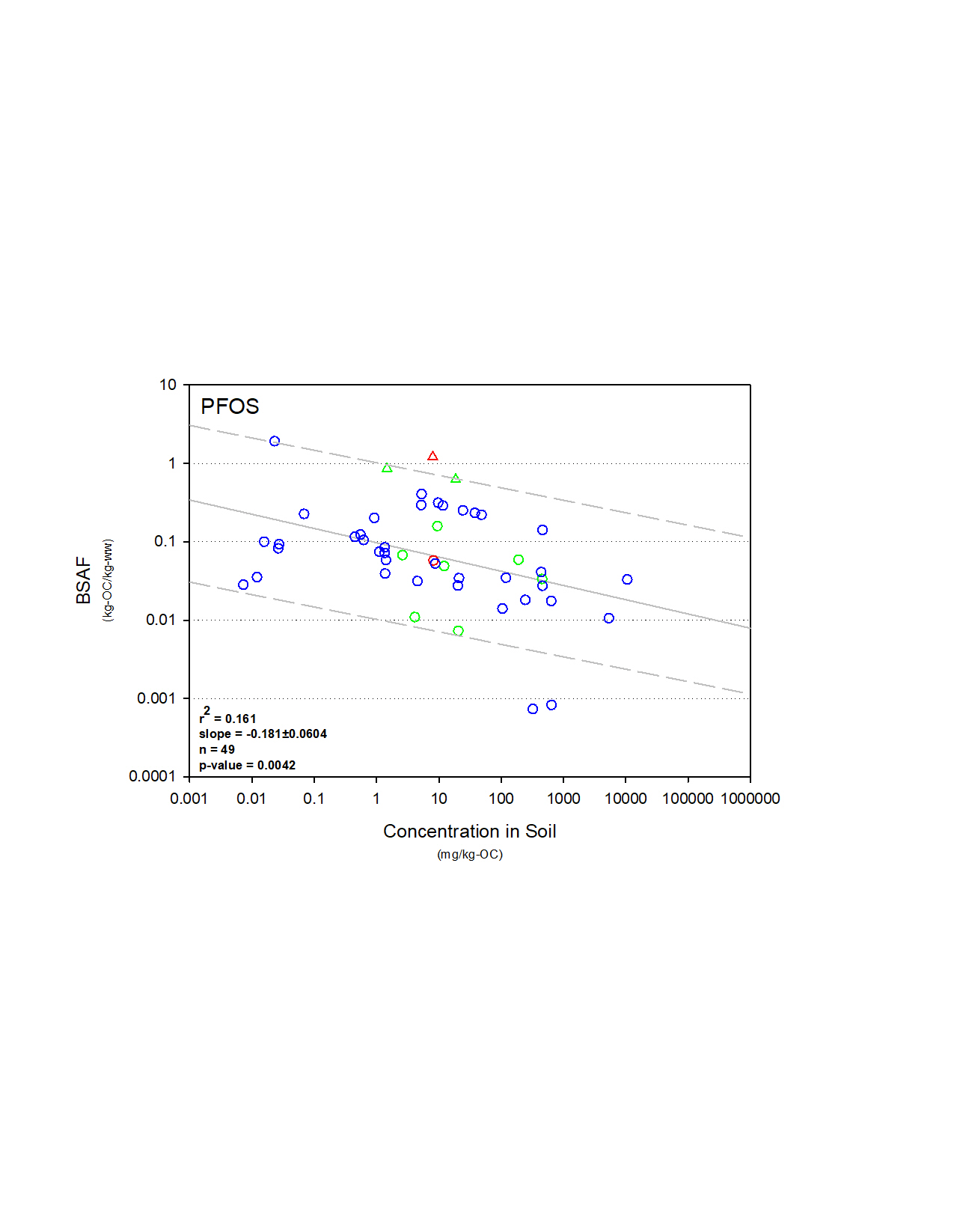


Figure S1L. For PFOS: Distribution plot of BSAFs and plot of BSAF (kg-OC/kg-ww) vs concentration in soil (mg/kg-OC). Measurement location: laboratory (circle) and field (triangle). Study quality ranking: high (red), medium (green) and low (blue). Regression line (solid) and statistics (slope ± standard error) along with lines 10-fold higher and lower are shown in BSAF vs concentration soil plot. When data points overlap, second point is drawn a bit larger.


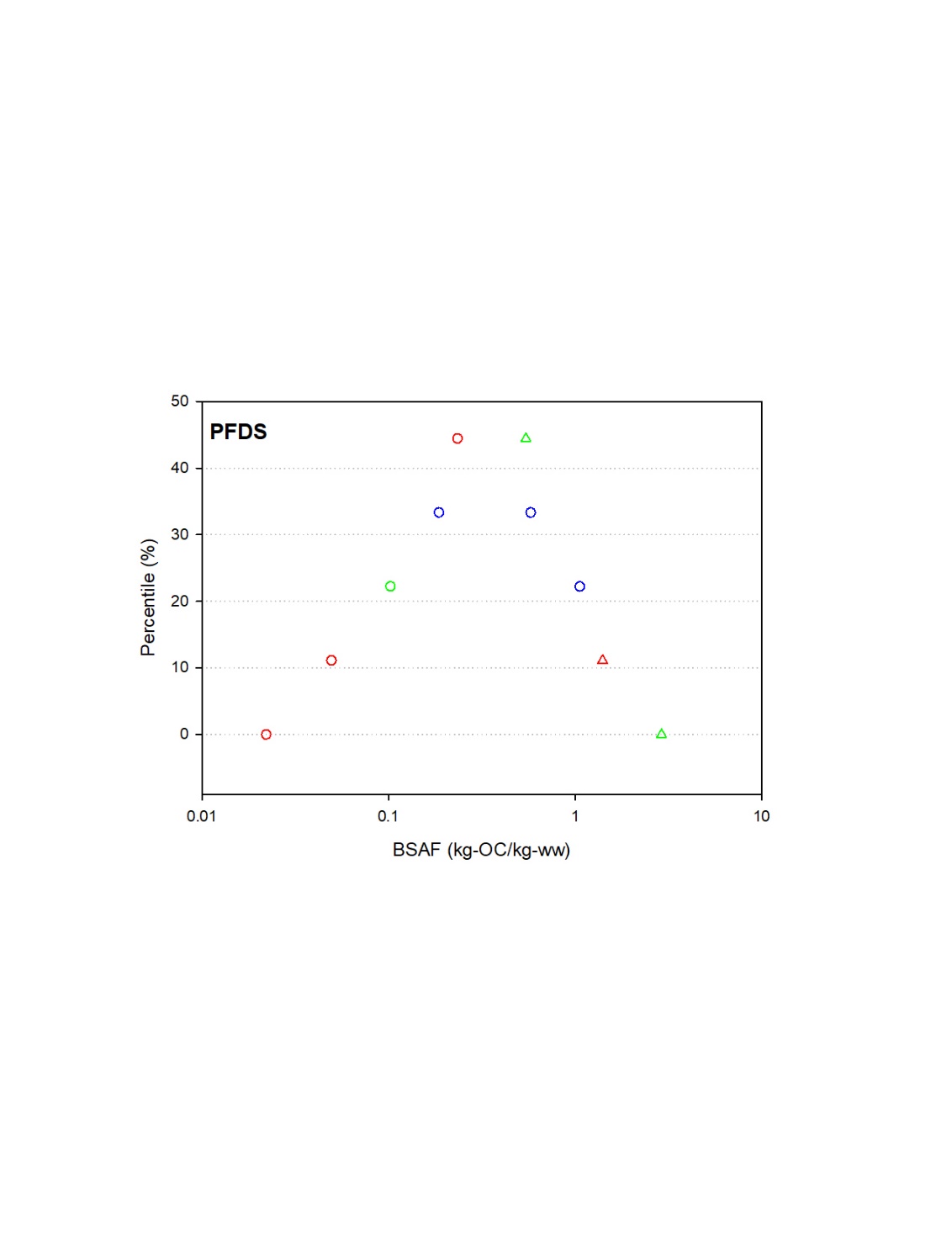


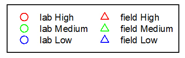


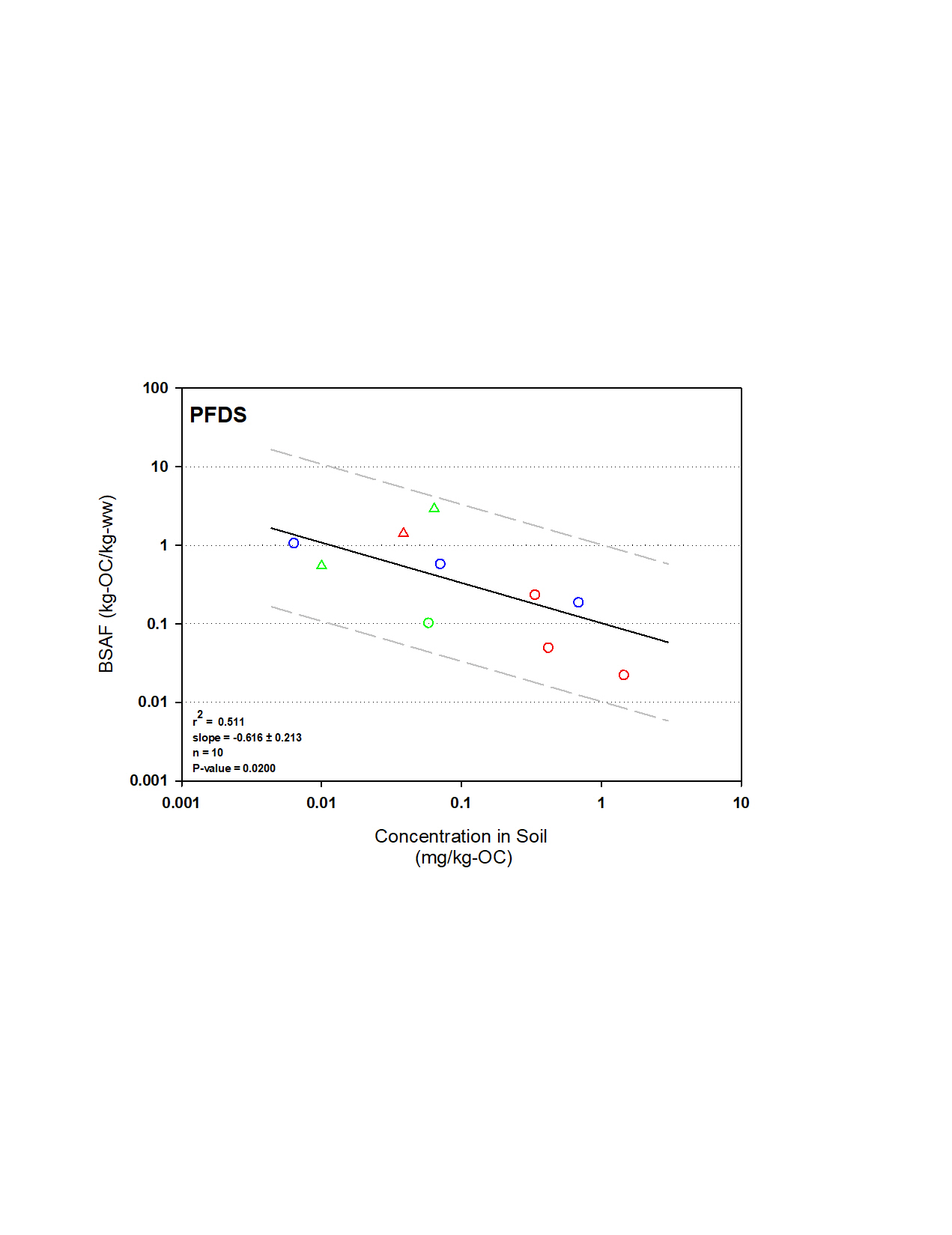


Figure S1M. For PFDS: Distribution plot of BSAFs and plot of BSAF (kg-OC/kg-ww) vs concentration in soil (mg/kg-OC). Measurement location: laboratory (circle) and field (triangle). Study quality ranking: high (red), medium (green) and low (blue). Regression line (solid) and statistics (slope ± standard error) along with lines 10-fold higher and lower are shown in BSAF vs concentration soil plot. When data points overlap, second point is drawn a bit larger.

**References**

Bräunig, Jennifer, Christine Baduel, Craig M Barnes, and Jochen F Mueller. 2019. 'Leaching and bioavailability of selected perfluoroalkyl acids (PFAAs) from soil contaminated by firefighting activities', *Science of The Total Environment*, 646: 471-79.

Chen, Meng, Qiang Wang, Yumin Zhu, Lingyan Zhu, Bowen Xiao, Menglin Liu, and Liping Yang. 2019. 'Species dependent accumulation and transformation of 8: 2 polyfluoroalkyl phosphate esters in sediment by three benthic organisms', *Environment international*, 133: 105171.

Das, Piw, Mallavarapu Megharaj, and Ravi Naidu. 2015. 'Perfluorooctane sulfonate release pattern from soils of fire training areas in Australia and its bioaccumulation potential in the earthworm *Eisenia fetida*', *Environmental Science and Pollution Research*, 22: 8902-10.

He, Wenxiang, Mallavarapu Megharaj, and Ravi Naidu. 2016. 'Toxicity of perfluorooctanoic acid towards earthworm and enzymatic activities in soil', *Environmental monitoring and assessment*, 188: 1-7.

Higgins, Christopher P, Pamela B McLeod, Laura A MacManus-Spencer, and Richard G Luthy. 2007. 'Bioaccumulation of perfluorochemicals in sediments by the aquatic oligochaete *Lumbriculus variegatus*', *Environmental Science & Technology*, 41: 4600-06.

Jarjour, Julie, Bei Yan, Gabriel Munoz, Mélanie Desrosiers, Sébastien Sauvé, and Jinxia Liu. 2022. 'Reduced bioaccumulation of fluorotelomer sulfonates and perfluoroalkyl acids in earthworms (*Eisenia fetida*) from soils amended with modified clays', *Journal of Hazardous Materials*, 423: 126999.

Jin, Bosen, Swetha Mallula, Svetlana A Golovko, Mikhail Y Golovko, and Feng Xiao. 2020. 'In vivo generation of PFOA, PFOS, and other compounds from cationic and zwitterionic per-and polyfluoroalkyl substances in a terrestrial invertebrate (Lumbricus terrestris)', *Environmental Science & Technology*, 54: 7378-87.

Karnjanapiboonwong, Adcharee, Sanjit K Deb, Seenivasan Subbiah, Degeng Wang, and Todd A Anderson. 2018. 'Perfluoroalkylsulfonic and carboxylic acids in earthworms (*Eisenia fetida*): Accumulation and effects results from spiked soils at PFAS concentrations bracketing environmental relevance', *Chemosphere*, 199: 168-73.

Rich, Courtney D, Andrea C Blaine, Lakhwinder Hundal, and Christopher P Higgins. 2015. 'Bioaccumulation of perfluoroalkyl acids by earthworms (*Eisenia fetida*) exposed to contaminated soils', *Environmental Science & Technology*, 49: 881-88.

Sobhani, Zahra, Cheng Fang, Ravi Naidu, and Mallavarapu Megharaj. 2021. 'Microplastics as a vector of toxic chemicals in soil: Enhanced uptake of perfluorooctane sulfonate and perfluorooctanoic acid by earthworms through sorption and reproductive toxicity', *Environmental Technology & Innovation*, 22: 101476.

Wang, Zhifeng, Fangjie Qi, Yanfeng Shi, Zhibin Zhang, Lei Liu, Chaona Li, and Lei Meng. 2022. 'Evaluation of single and joint toxicity of perfluorooctanoic acid and arsenite to earthworm (*Eisenia fetida*): A multi-biomarker approach', *Chemosphere*, 291: 132942.

Wen, Bei, Hongna Zhang, Longfei Li, Xiaoyu Hu, Yu Liu, Xiao-quan Shan, and Shuzhen Zhang. 2015. 'Bioavailability of perfluorooctane sulfonate (PFOS) and perfluorooctanoic acid (PFOA) in biosolids-amended soils to earthworms (*Eisenia fetida*)', *Chemosphere*, 118: 361-66.

Zhao, Shuyan, Shuhong Fang, Lingyan Zhu, Li Liu, Zhengtao Liu, and Yahui Zhang. 2014. 'Mutual impacts of wheat (*Triticum aestivum L.*) and earthworms (*Eisenia fetida*) on the bioavailability of perfluoroalkyl substances (PFASs) in soil', *Environmental Pollution*, 184: 495-501.

Zhao, Shuyan, Tianqi Liu, Lingyan Zhu, Liping Yang, Yvlu Zong, Huanting Zhao, Longhui Hu, and Jingjing Zhan. 2021. 'Formation of perfluorocarboxylic acids (PFCAs) during the exposure of earthworms to 6: 2 fluorotelomer sulfonic acid (6: 2 FTSA)', *Science of The Total Environment*, 760: 143356.

Zhao, Shuyan, Xinxin Ma, Shuhong Fang, and Lingyan Zhu. 2016. 'Behaviors of N-ethyl perfluorooctane sulfonamide ethanol (N-EtFOSE) in a soil-earthworm system: transformation and bioaccumulation', *Science of The Total Environment*, 554: 186-91.

Zhao, Shuyan, Qiao Yang, Bohui Wang, Yihong Peng, Jingjing Zhan, and Lifen Liu. 2018. 'Effects of combined exposure to perfluoroalkyl acids and heavy metals on bioaccumulation and subcellular distribution in earthworms (*Eisenia fetida*) from co-contaminated soil', *Environmental Science and Pollution Research*, 25: 29335-44.

Zhao, Shuyan, and Lingyan Zhu. 2017. 'Uptake and metabolism of 10: 2 fluorotelomer alcohol in soil-earthworm (Eisenia fetida) and soil-wheat (*Triticum aestivum* L.) systems', *Environmental Pollution*, 220: 124-31.

Zhao, Shuyan, Lingyan Zhu, Li Liu, Zhengtao Liu, and Yahui Zhang. 2013. 'Bioaccumulation of perfluoroalkyl carboxylates (PFCAs) and perfluoroalkane sulfonates (PFSAs) by earthworms (*Eisenia fetida*) in soil', *Environmental Pollution*, 179: 45-52.

Zhu, Yumin, Yibo Jia, Menglin Liu, Liping Yang, Shujun Yi, Xuemin Feng, and Lingyan Zhu. 2021. 'Mechanisms for tissue-specific accumulation and phase I/II transformation of 6: 2 fluorotelomer phosphate diester in earthworm (*M. guillelmi*)', *Environment international*, 151: 106451.
